# MedBot vs RealDoc: efficacy of large language modeling in physician-patient communication for rare diseases

**DOI:** 10.1093/jamia/ocaf034

**Published:** 2025-02-25

**Authors:** Magdalena T Weber, Richard Noll, Alexandra Marchl, Carlo Facchinello, Achim Grünewaldt, Christian Hügel, Khader Musleh, Thomas O F Wagner, Holger Storf, Jannik Schaaf

**Affiliations:** Institute of Medical Informatics, University Medicine Frankfurt, Goethe University Frankfurt, Frankfurt 60590, Germany; Institute of Medical Informatics, University Medicine Frankfurt, Goethe University Frankfurt, Frankfurt 60590, Germany; Institute of Medical Informatics, University Medicine Frankfurt, Goethe University Frankfurt, Frankfurt 60590, Germany; Santagostino Medical Center, Bologna 40138, Italy; Department of Respiratory Medicine and Allergology, University Medicine Frankfurt, Goethe University Frankfurt, Frankfurt 60590, Germany; HELIOS Dr Horst Schmidt Kliniken Wiesbaden, Klinik für Pneumologie, Wiesbaden 65199, Germany; Department of Respiratory Medicine and Allergology, University Medicine Frankfurt, Goethe University Frankfurt, Frankfurt 60590, Germany; European Reference Network for Rare Respiratory Diseases (ERN-LUNG), University Medicine Frankfurt, Frankfurt 60590, Germany; Institute of Medical Informatics, University Medicine Frankfurt, Goethe University Frankfurt, Frankfurt 60590, Germany; Institute of Medical Informatics, University Medicine Frankfurt, Goethe University Frankfurt, Frankfurt 60590, Germany

**Keywords:** artificial intelligence, medical informatics, natural language processing, health communication, rare diseases

## Abstract

**Objectives:**

This study assesses the abilities of 2 large language models (LLMs), GPT-4 and BioMistral 7B, in responding to patient queries, particularly concerning rare diseases, and compares their performance with that of physicians.

**Materials and Methods:**

A total of 103 patient queries and corresponding physician answers were extracted from EXABO, a question-answering forum dedicated to rare respiratory diseases. The responses provided by physicians and generated by LLMs were ranked on a Likert scale by a panel of 4 experts based on 4 key quality criteria for health communication: correctness, comprehensibility, relevance, and empathy.

**Results:**

The performance of generative pretrained transformer 4 (GPT-4) was significantly better than the performance of the physicians and BioMistral 7B. While the overall ranking considers GPT-4’s responses to be mostly correct, comprehensive, relevant, and emphatic, the responses provided by BioMistral 7B were only partially correct and empathetic. The responses given by physicians rank in between. The experts concur that an LLM could lighten the load for physicians, rigorous validation is considered essential to guarantee dependability and efficacy.

**Discussion:**

Open-source models such as BioMistral 7B offer the advantage of privacy by running locally in health-care settings. GPT-4, on the other hand, demonstrates proficiency in communication and knowledge depth. However, challenges persist, including the management of response variability, the balancing of comprehensibility with medical accuracy, and the assurance of consistent performance across different languages.

**Conclusion:**

The performance of GPT-4 underscores the potential of LLMs in facilitating physician-patient communication. However, it is imperative that these systems are handled with care, as erroneous responses have the potential to cause harm without the requisite validation procedures.

## Background and significance

The advent of large language models (LLMs) led to a public comeback of artificial intelligence (AI), with profound implications for a variety of sectors, including health care.^1^ Models such as OpenAI’s generative pretrained transformer 4 (GPT-4) are capable of understanding and generating human-like text and performing various tasks, such as summarization, translation, and even coding.^2^ Given their broad applicability, it seems feasible to advance automation in a variety of fields by employing LLMs, a prospect that is currently being widely explored.^3^ Until recently, chatbots were primarily utilized for customer service and straightforward enquiries. With the advent of LLMs as the underlying technology, they are now capable of performing more complex tasks, as they are able to comprehend the context and memorize prior conversations.^4^ One of the principal benefits of employing LLMs in the health-care sector is their capacity to facilitate improved access to medical information. These models can provide round-the-clock support, tending to patients with immediate responses to common medical questions and helping to alleviate disparities in health-care access, particularly in underserved regions.^5^ By augmenting traditional health-care services, LLMs can reduce the burden on health-care professionals.^6^ In addition to convenience, LLMs can deliver personalized advice, drawing on vast databases of medical knowledge to tailor responses to individual patient needs. This adaptability has the potential to improve patient outcomes by making health care more accessible, responsive, and efficient.^7^ Despite their promise, the integration of LLMs into health care has sparked debate, particularly regarding their accuracy, reliability, and empathy in patient interactions.^8^

Various studies were contrived to evaluate the effectiveness of LLMs in diverse health-care settings. They were utilized to draft informed consent forms at a level of comprehension deemed suitable for the majority of patients^9^ or to generate informative content that can be provided to patients.^10^ A number of studies have examined the capacity of LLMs to respond to patient queries, with comparisons drawn between the responses and those of physicians.^11–13^ The study by Ayers et al. evaluated ChatGPT-3.5 to respond to patient questions compared to physicians on public social media forum (Reddit’s r/AskDocs). In a sample of 195 questions, chatbot responses were rated significantly higher in both quality and empathy than physician responses.^11^ Bernstein et al. compared ChatGPT-3.5 responses to ophthalmologist-written answers on 200 eye care questions, finding that while chatbot responses were often identifiable as AI-generated, they were generally accurate and posed a comparable risk of harm to human responses.^12^ He et al. assessed ChatGPT-4 and ERNIE Bot’s effectiveness in answering 239 autism-related questions in Chinese, finding that while physicians’ responses scored highest in relevance, accuracy, and usefulness, ChatGPT showed greater empathy, suggesting LLMs’ potential to complement patient support in health-care settings.^13^

As LLM-based chatbots demonstrate potential for improving physician-patient communication by providing empathetic and accessible responses, this study examines their suitability for use in the context of rare diseases. In the field of rare diseases, there is a paucity of accurate information, which often results in patients lacking access to specialized guidance.^14^ Moreover, in rural regions, the accessibility of specialized services and competent medical professionals who are equipped to furnish suitable follow-up care for patients afflicted with rare diseases is constrained.^15^

## Objective

The objective of this comparative study was to evaluate the potential of GPT-4 and the open-source model BioMistral 7B in supporting patient question-answering for rare diseases. In addition to assessing the correctness, comprehensibility, relevance, and empathy of the LLMs’ responses, the study also included a direct comparison with answers provided by physicians. Evaluators, who were physicians themselves, assessed both the LLM-generated responses and those of their peers to identify strengths and weaknesses across the evaluation criteria. A further question posed in the study was whether participating physicians would consider utilizing LLMs to provide template responses to queries, and whether such a system could effectively reduce the time spent on this task. To the best of our knowledge, this study represents the first of its kind to evaluate the question-answering capabilities of LLMs within the context of rare diseases.

## Materials and methods

The patient queries and corresponding physician responses used in this study were derived from the archives of the online expert advisory board EXABO. These 142 cases were documented between March 2019 and February 2024, with 93 in German and 49 in English.^16^ EXABO (www.exabo.eu) was developed to provide support to patients with rare respiratory diseases and facilitate access to reliable information. It is maintained by the European reference network for rare respiratory diseases (ERN-LUNG).^17^^,^^18^ This platform is a valuable resource for patients, their families, and even physicians, who often turn to it for guidance on treatment options, diagnostic approaches, or recommendations for specialized care centers. All patient queries and physician responses were subjected to a review process by A.M. to extract the pertinent diseases, namely sarcoidosis, primary ciliary dyskinesia, idiopathic pulmonary fibrosis, hypersensitivity pneumonitis, bronchiectasis, and bronchopulmonary dysplasia. Patient queries are anonymized prior to their inclusion in the archives, in accordance with the requirements of patient privacy. The answers were screened by M.T.W. and A.M., 39 pairs unsuitable for the study were manually removed. A query was classified as unsuitable when the disease in question was referenced solely in the subject line and not within the body of the query. Additionally, queries that did not pertain to a medical condition, such as test questions or inquiries about the EXABO platform or ERN-LUNG, were also excluded.

Prior to this study, the physician responses were edited in accordance with the methodology outlined in Weber et al., whereby the following information was removed, including titles, names, quotes, and unrelated content such as apologies for delayed responses by the physician.^19^ This editing process ensured that the physician responses were less distinguishable from those generated by the LLMs, thus maintaining the evaluators‘ impartiality. Following editing, the answers were manually reviewed to guarantee no information was lost and that the meaning was not altered.

### LLM response generation

Two LLMs were selected for comparison, BioMistral 7B and GPT-4.^2^^,^^20^ Generative pretrained transformer 4 is one of the largest models currently available, with approximately more than 1.7 trillion parameters, trained by the company OpenAI to engage in conversation about various topics.^2^ The BioMistral 7B model is an open-source model comprising 7 billion parameters. It was trained on PubMed Central using Mistral as a foundation and benchmarked on medical question-answering tasks.^20^

The GPT-4 model was accessed through a customized version of ChatGPT, which permits uploading of documents. These documents are processed into embeddings, thereby enabling the model to retrieve relevant information through semantic search.^21^ The documents for customization were sourced from Orphanet,^22^ containing details on the diseases in question, including experts contact information. Orphanet is a comprehensive database dedicated to the provision of information on rare diseases and orphan drugs. The objective of Orphanet is to facilitate improvements in the diagnosis, care, and treatment of patients with rare conditions.^22^ The documents were extracted as text files in English.

The GPT-4 generated responses were obtained by prompting each patient query in a separate GPT-4 chat, with only the initial response included in the study. BioMistral 7B was adapted using low-rank adaption (LoRA)^23^ with the same disease-specific information from Orphanet used to customize GPT-4. Low-rank adaption is a method that enables the efficient fine-tuning of LLMs. This is achieved by freezing most model parameters and updating only small, low-rank matrices. The result is a significant reduction in computational cost and storage requirements.^23^ The model was trained for 10 epochs, with a rank value of 8, corresponding to the number of the parameters in the adaption layer, and an alpha value of 16, which is a scaling factor determining the influence of the adaption layers weights on the base model. The relatively low rank value and double alpha value were chosen, considering the amount of additional information, to effectively adapt the model within a limited training time. The responses generated by BioMistral 7B were obtained via TextGen WebUI, an openly accessible user interface for interaction with LLMs,^24^ with each query posed separately, and only the initial response retained. In order to ensure the integrity of the study, responses indicating that the answers were provided by an AI were excluded, as were the corresponding physician and GPT-4 responses.

### Study design

The study was conducted as a comparative blind study, where a group of evaluators was asked to rank the responses from GPT-4, BioMistral 7B, and physicians on a Likert scale across 4 categories: correctness, comprehensibility, relevance, and empathy. The group of evaluators, comprising physicians in internal medicine or pneumonology, excluding those involved with the EXABO platform, was selected by purposeful sampling. A total of 7 suitable experts were invited to participate in the study. An expert was defined as a person with comprehensive and authoritative knowledge in one of the relevant domains, acquired through professional practice, training, and experience.^25^^,^^26^ Furthermore, the experts gave evidence of comprehensive proficiency in both German and English. The study was conducted from June 14, 2024 to July 14, 2024 using the online survey platform SurveyMonkey,^27^ which randomized the order of responses and ensured that evaluators could not directly compare the different answers to the same query. All patient queries and responses can be found in the Supplementary Material S1. For each response, participants were required to assign a ranking on a Likert scale with 5 options, convertible to ranks. Likert scales are typically employed in the context of measuring perceptions that are not amenable to concrete or objective measurement. They facilitate the quantitative estimation of subjective traits through the utilization of ordinal scales, thereby providing an objective framework for analysis.^28^^,^^29^ Here, the Likert scale is used in an untraditional way to objectively assess the correctness of responses by providing detailed ranking instructions for different levels of correctness.^28^ The detailed ranking instructions for the 4 categories are provided in Table 1.

**Table 1.** Ranking instructions for the 4 categories, given to the group of evaluators in German, here translated to English.

		Rank
**Correctness**		
Absolutely incorrect	Grave mistakes, that could affect patient health, not adhering to medical standards	1
Incorrect	Mistakes or inaccuracies substantially compromising medical quality	2
Partly correct	Partly correct with several noncritical mistakes or omissions	3
Mostly correct	Mostly correct with minor noncritical inaccuracies	4
Absolutely correct	Fully correct, adhering to current medical standards and evidence-based practice	5
**Comprehensibility**		
Absolutely incomprehensible	Not comprehensible, complex technical language, confusing	1
Incomprehensible	Hardly comprehensible, includes technical terms, unclear wording	2
Partly comprehensible	Partly comprehensible, some technical terms or unclear wording	3
Mostly comprehensible	Mostly comprehensible, few technical terms or difficult wording	4
Absolutely comprehensible	Comprehensible even to laymen, without technical terms and complex wording	5
**Relevance**		
Absolutely irrelevant	Does not consider the question, given information is not relevant to the patient	1
Irrelevant	Barely considers the question, given information is mostly irrelevant	2
Partly relevant	Partly considers the question, contains irrelevant information	3
Mostly relevant	Considers the question, barely contains irrelevant information	4
Absolutely relevant	Concisely answers the question without irrelevant information	5
**Empathy**		
Absolutely unemphatic	Distant, unemphatic, does not take the patient’s emotions into account	1
Unemphatic	Mostly unemphatic and distanced, shows little consideration for patients’ emotion	2
Partly emphatic	Some consideration for patients’ concerns, still distanced and impersonal	3
Mostly emphatic	Friendly and supportive, taking the patients feelings into account	4
Absolutely emphatic	Very empathic, high level of consideration, showing active support for patient concerns	5

The assessment of correctness determined whether the medical information provided in the response adhered to the prevailing health-care standards. The evaluation of comprehensibility measured whether the answer was readily understandable from a patient perspective. Relevance evaluated whether the response remained pertinent to the topic and the examination of empathy determined whether the answer considered the emotional state of the patient.

The group of evaluators was not informed of the origin of the responses, the true objective of the study, or the involvement of AI. Once the evaluation was completed, the objective of the study, comparing AI-generated and physician responses to patient queries, was revealed. The evaluators were asked 7 questions for their opinion on the use of AI in health care and whether they felt able to distinguish LLM-generated responses from those produced by physicians.

### Data analysis

The rankings were analyzed using descriptive statistics, the intraclass correlation coefficient (ICC), the Kruskal-Wallis test, and the Dunn’s test.^30^ For each category, the mean, SD, and median rankings were calculated for each group, namely GPT-4, BioMistral 7B, and physicians. To assess interrater reliability, the ICC, more specifically the 2-way mixed effects model, ICC(3,1), was calculated. A high ICC value, close to 1, represents strong agreement between raters, whereas a low ICC value, close to zero, indicates differing ratings. In general ICC values above 0.75 are considered to indicate a high level of reliability.^31^ The Kruskal-Wallis test was employed to ascertain whether there were significant differences between the 3 independent groups—GPT-4, BioMistral 7B, and physicians—by calculating and comparing ranking values. A *P*-value that is less than the predefined significance threshold of 0.05 indicates that the observed difference is statistically significant. Pairwise group comparisons were conducted using the Dunn’s test, similar to a *t*-test but for nonparametric data and with Bonferroni correction. To ensure reproducibility of the results, the statistical tests were independently conducted by A.M. and M.T.W. with SPSS and R.N. with python (version 3.11.5), using scipy.stats and scikit-posthocs.^32–35^

## Results

**Figure 1.**
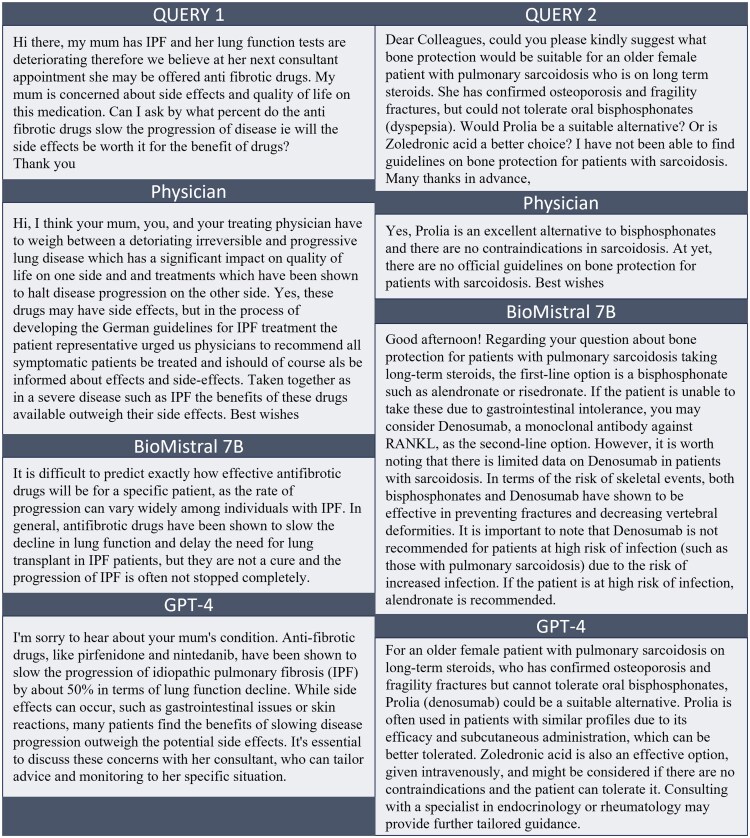
Example queries with the respective physician, BioMistral 7B, and GPT-4 responses.

Four of the 7 experts invited completed the survey including the additional questions, the 3 incomplete evaluations were not included for the analysis. In 4 instances, the BioMistral 7B model explicitly indicated in its response that the answer in question was produced by an AI. To avoid bias and to follow the blind study design, these responses were not included in the study’s further evaluation. Ultimately, 103 patient queries, 23 in English and 80 in German, with corresponding answers were included. Figure 1 contains 2 example queries with the respective answers.

**Figure 2.**
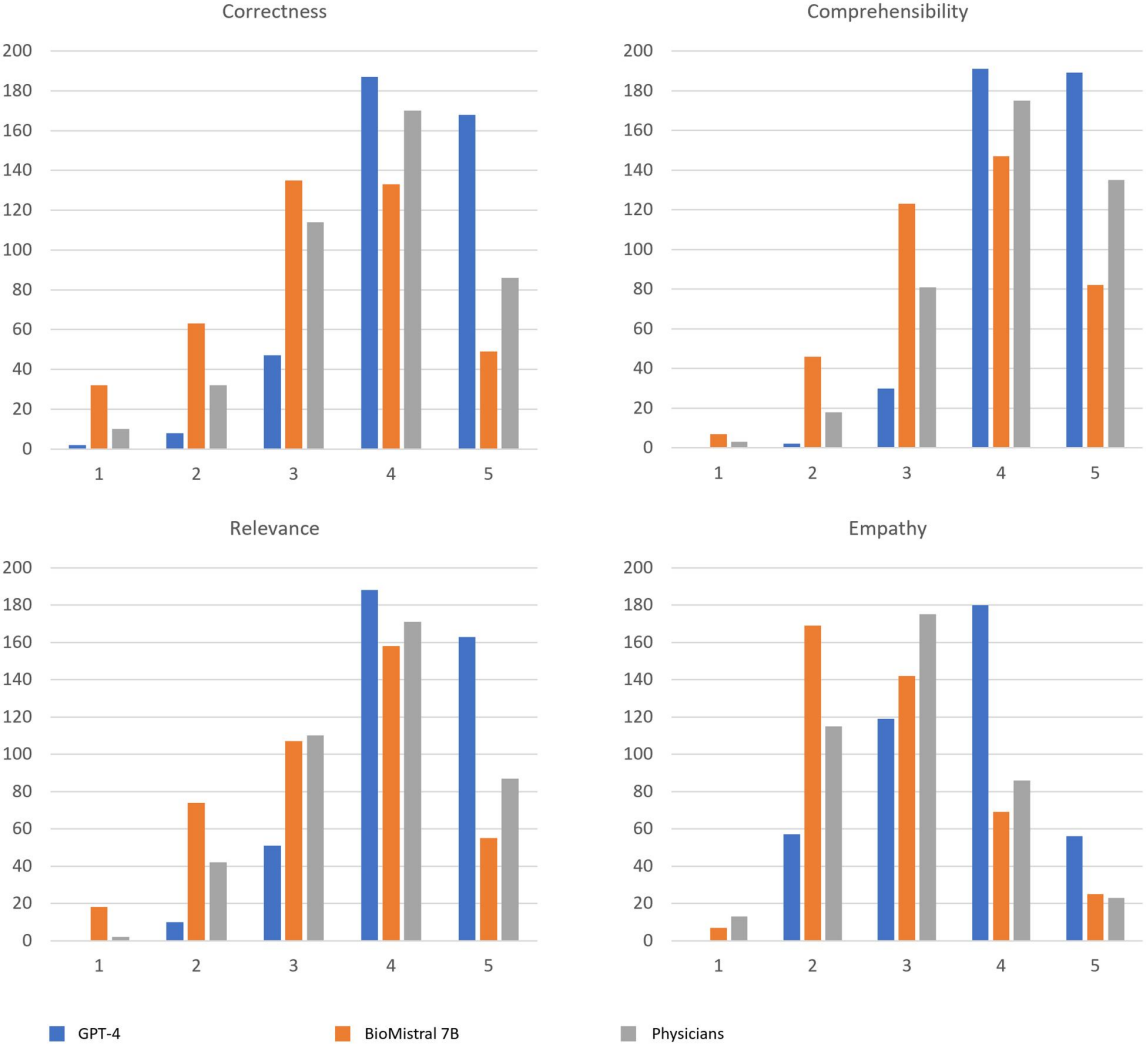
Distribution of responses across ranks for the 4 categories. The *y*-axis represents the total number of responses, and the *x*-axis indicates the respective Likert scale rank, as defined in Table 1.

**Figure 3.**
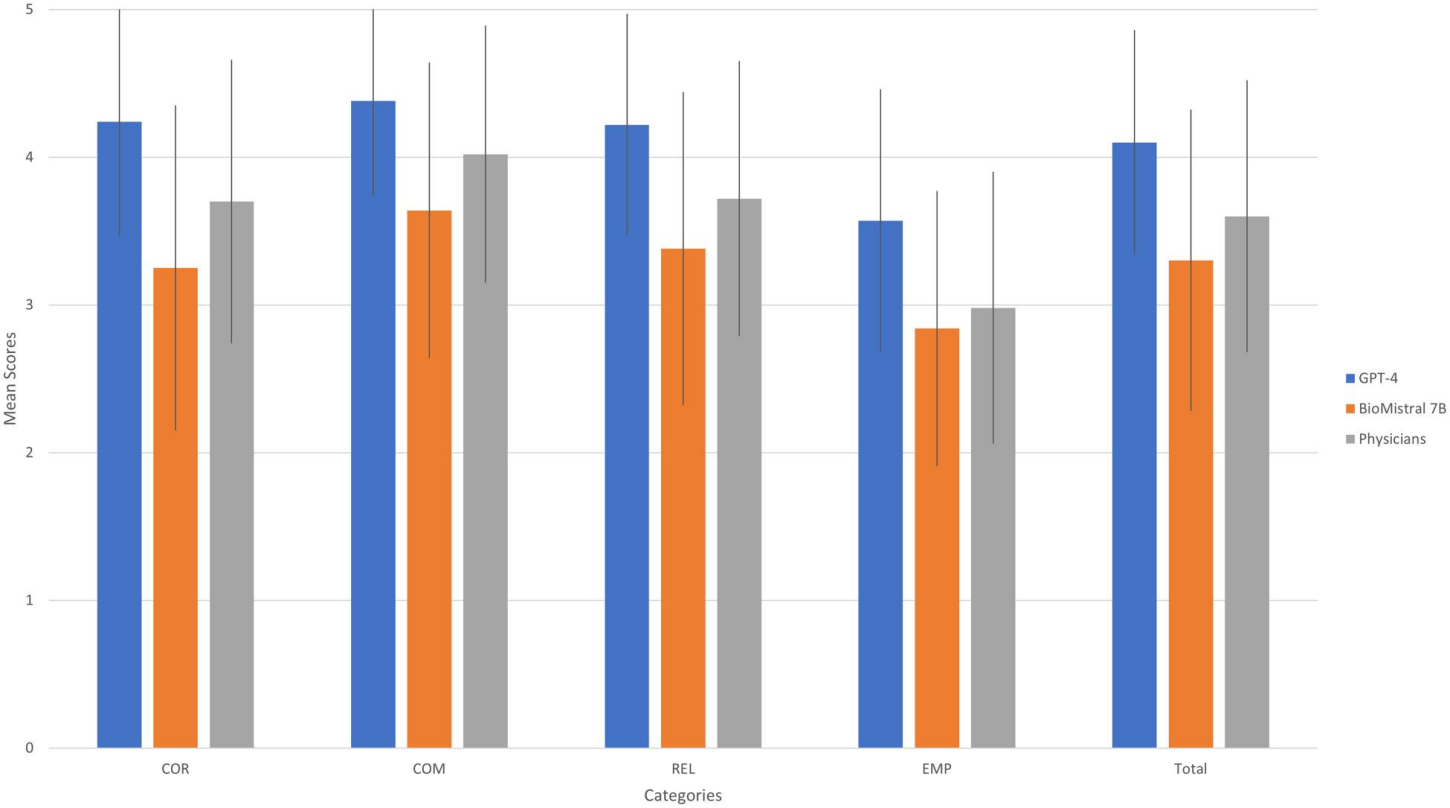
Comparison of mean evaluation scores of GPT-4, BioMistral 7B, and physicians across the categories of correctness (COR), comprehensibility (COM), relevance (REL), and empathy (EMP).

Generative pretrained transformer 4’s answers achieved the highest mean rankings (4.1, SD = 0.76) across all 4 categories, while those for BioMistral 7B (3.3, SD = 1.02) resulted consistently in the lowest mean. In terms of correctness, the median ranking was 4 for both GPT-4 and physician responses, and 3 for BioMistral 7B. In the empathy category, the median ranking was 3 for both BioMistral 7B and the physician, while GPT-4 achieved a 4. All groups had a median ranking of 4 in both relevance and comprehensibility. Detailed results are displayed in Table 2. The absolute number of the respective scores for all evaluated responses, which amount to 412 as 4 physicians evaluated each 103 responses, is displayed in Figure 2. Figure 3 shows the mean evaluation scores (with SD) for GPT-4, BioMistral 7B, and physicians across the categories.

**Table 2.** Median, mean and SD for the 4 categories—correctness, comprehensibility, relevance, and empathy.

	GPT-4		BioMistral 7B		Physicians	
	Median	Mean (SD)	Median	Mean (SD)	Median	Mean (SD)
Correctness	4	4.24 (0.77)	3	3.25 (1.10)	4	3.70 (0.96)
Comprehensibility	4	4.38 (0.64)	4	3.64 (1.00)	4	4.02 (0.87)
Relevance	4	4.22 (0.75)	4	3.38 (1.06)	4	3.72 (0.93)
Empathy	4	3.57 (0.89)	3	2.84 (0.93)	3	2.98 (0.92)
Total		4.1 (0.76)		3.3 (1.02)		3.6 (0.92)

The ICC values were 0.728 for correctness, 0.629 for comprehensibility, 0.701 for relevance, and 0.663 for empathy. All values are within the limits of moderate reliability, although closer to the upper limit of 0.75.

The Kruskal-Wallis test demonstrated that there were notable discrepancies in the rankings assigned to the groups, with *P*<.001 across all categories. The Dunn’s test demonstrated that the responses provided by GPT-4, BioMistral 7B, and physicians exhibited notable discrepancies across the categories of “correctness,” “comprehensibility,” and “relevance,” with *P*-values remaining below the significance threshold of .001 for all pairs of groups. However, within the category of “empathy” the discrepancy in ranking between BioMistral 7B and the physicians was not statistically significant, as indicated by a *P*=.058, exceeding the significance threshold of .05. Detailed results are displayed in the Supplementary Material S2.

A compendium of the answers given to the additional study questions is displayed in Table 3, the full answers can be found in the Supplementary Material S3. The physicians align in their expectation that an LLM could be helpful and relieve some of their workload, but also consider validation very important. They generally expect lower comprehensibility and higher generality in responses generated by LLMs.

**Table 3.** Compendium of the responses given by the physicians to the additional questions, the responses were translated from German to English and summarized for this representation.

Question	Summarized response
To what extent could chatbot responses help reduce the workload of doctors? Would you consider such an answer as a first draft?	All participants consider template answers from a chatbot helpful and would use them. They’d expect it to provide basic information gathered from literature
What potential risks do you see with the use of chatbots, especially in terms of misinformation or overlooking critical information?	The participants mostly agree that these risks are avoidable if the tool adheres to literature and guidelines and by thorough validation
What considerations would you make before you consider using a chatbot in practice?	The participants would check the risk of hallucination, conduct tests, and consider privacy, usability, and cost
How do you assess the ethical aspects of using a chatbot, particularly regarding patient confidentiality?	The participants agree that protecting patient privacy is of utmost importance but expect the protection to be possible. One evaluator sees no ethical concern when used as a support system
Are there any retrospective characteristics or clues in the answers that make you suspect whether they come from a doctor or a chatbot? If so, which ones?	The participants state formal correctness but illogicality, incomprehensibility, incomplete sentences, inflexibility, generality, and study citations as clues for chatbot answers
Did you notice any major differences in the answers? If so, what differences did you notice?	The participants noticed differences in the quality of the answers, but rather a spectrum of variability than distinct categories
What legal challenges do you see when implementing an AI-based chatbot in physician-patient communication?	The participants’ opinions differ on that topic. Some refer to privacy hurdles, whereas others are optimistic that it could be implemented as a support system

## Discussion

This study examined the question-answering capabilities of LLMs in comparison to those of physicians, with a particular focus on patient queries pertaining to rare diseases. EXABO furnished an optimal source of authentic patient inquiries and physician responses for this study, enabling the reproduction of real-world scenarios without comprising privacy, ensuring this study’s validity and significance. The evaluation was conducted across 4 key dimensions: correctness, comprehensibility, relevance, and empathy. Generative pretrained transformer 4 demonstrated significantly superior performance in all categories when compared to physicians (*P* < .001). Conversely, BioMistral 7B exhibited a significantly inferior performance in the categories of correctness and comprehensibility (*P* < .001). However, in the empathy category, the ranking for BioMistral 7B did not differ significantly from that of physicians (*P* = .058). The interrater reliability was moderate with ICC values between 0.728 and 0.629 across all categories. The highest interrater reliability was achieved for the category of correctness.

Following the completion of the ranking phase, medical experts were invited to share their perspectives on the potential applications of AI in the context of health care. It is noteworthy that while some experts expressed reservations about the accuracy and comprehensibility of AI, all were able to envisage scenarios where LLMs could be used to support their daily work. For information to be beneficial, it must be comprehensive yet straightforward, avoiding complex terminology or assumptions about patients’ familiarity with health-care concepts that may be new to them.^36–38^ Empathetic communication further builds trust, encouraging adherence to medical advice and enhancing satisfaction for both patients and providers.^39^

Generative pretrained transformer 4 demonstrated consistent superiority, which is likely attributable to its advanced communication skills and broad knowledge base.^2^ As previously stated, physicians are frequently confronted with demanding work schedules. This may have constrained the extent of effort that could be invested in the responses, contributing to the observed differences, particularly in the comprehensibility category. The inferior performance of BioMistral 7B in comparison to GPT-4 can be attributed to its lower parameter count and apparent linguistic deficits in German responses compared to GPT-4, which was developed to excel in human communication and is highly proficient in English and German.^2^ BioMistral 7B was chosen as an open-source alternative finetuned for the medical field.^20^ In the context of medical applications, where data privacy is of paramount importance, open-source solutions are essential. This is because they can be run locally, thereby ensuring that no data leaves the hospital infrastructure and that there is no need to transfer sensitive information to a cloud.^40^

While GPT-4 has attained the highest mean ranking for all categories, including correctness, there have been instances where its responses have been rated as absolutely incorrect or incorrect. Erroneous responses have the potential to compromise patient well-being. As LLMs generate text based on probabilistic models rather than true understanding, errors remain an inherent challenge.^41^ Furthermore, for nonopen-source models such as GPT-4 the quality of the training data cannot be verified, meaning that while the answer may sound plausible it may contain harmful advice.^42^ On a positive note, the utilization of LLMs as a support system has the potential to mitigate errors by virtue of the fact that their errors differ in some instances from those of humans and thus may be more readily identified by medical professionals.^43^ Conversely, errors made by physicians due to time constraints or a paucity of recent knowledge may decrease.^44^

The representativeness of the study is limited by the fact that only 4 physicians participated in the ranking of the responses. However, comparable studies faced the same limitations,^11^^,^^13^ and the ICC values were in the higher moderate range, indicating conformity between the evaluator’s rankings. It is conceivable that individual attitudes, whether favorable or unfavorable, could impact the rankings and consequently may compromise the objectivity of the study. To minimize bias, it was essential that the evaluators were unaware of the involvement of LLMs in the study. It was important to ensure that the evaluators provide an accurate assessment of the responses. To this end, only experts in pneumonology or internal medicine with experience in the treatment of rare respiratory diseases and proficiency in English and German were invited to participate.

Furthermore, the responses generated by GPT-4 and BioMistral were frequently more extensive than those provided by physicians, which could have affected the assessment. It has been observed that response length can impact patient satisfaction and correlate with ranking in quality and empathy.^11^^,^^45^ In future studies, limiting the length of responses could enhance comparability between physician and LLM-generated answers. However, when LLMs are used as support tools, the additional length might enhance the quality of the response and overall patient satisfaction.

In order to enhance the relevance and accuracy of the model’s responses, additional disease-specific data from Orphanet were incorporated.^22^ This modification proved particularly beneficial, as a considerable proportion of EXABO users seek recommendations for specialized physicians or treatment centers—a need that aligns well with the specialized information provided by Orphanet. However, a limitation of this approach was that only English texts from Orphanet were utilized, while the dataset also included numerous German questions. This discrepancy in language could potentially introduce variability in the accuracy and depth of the responses, particularly in the German-language answers. It is recommended that future research endeavors systematically investigate the performance of large LLMs in different languages, depending on the language of the dataset provided.

In view of the capacity of GPT-4 and subsequent models to retrieve the latest information directly from the internet, it is imperative to assess the necessity of fine-tuning and customization. Fine-tuning is defined as the process of altering the fundamental parameters of an LLM by employing retraining with novel data, as illustrated by LoRA.^23^ In contrast, customization employs instructions and external documents to guide the LLM without effecting alterations to its core.^21^ On the one hand, fine-tuning and customization with high-quality, curated data, such as that provided by Orphanet in the form of disease profiles, ensures that responses adhere to a consistent and reliable knowledge base, thereby reducing the variability that might otherwise result from sourcing live web content of fluctuating quality. Conversely, internet-enabled models have the capacity to incorporate the latest medical knowledge and may adapt to evolving guidelines or emerging treatments more readily than a fine-tuned model that is limited to static datasets. Nevertheless, it cannot be assumed that data retrieved from the internet is from relevant sources like Orphanet, nor that it has been filtered to remove potential misinformation. In this context, retrieval-augmented generation (RAG) represents an intriguing potential avenue for exploration.^46^^,^^47^ Retrieval-augmented generation enables models to achieve a balance between the benefits of fine-tuning and dynamic retrieval by drawing on a curated set of trusted sources, such as Orphanet or other vetted medical repositories, rather than relying on unverified training data or the open internet. In the context of rare diseases, where guidelines and insights are constantly evolving, RAG allows for a hybrid solution, ensuring that responses are both current and trustworthy.

## Conclusion

The findings of this study indicate the potential of LLMs in assisting physicians in responding to patient queries on an online expert advisory system for rare diseases, with GPT-4 delivering promising results in terms of correctness, comprehensibility, and relevance. Further research is required to ascertain the extent to which LLM-generated responses can be relied upon to be of a consistently high quality across a wider range of patient queries, particularly in the context of rare diseases. Furthermore, it will be imperative to assess the extent to which physicians will still need to make adjustments to ensure that responses meet clinical standards.

## Supplementary Material

ocaf034_Supplementary_Data

## Data Availability

The data used for this study are available at www.exabo.eu and published within the Supplementary Material.

## References

[1] Thirunavukarasu AJ , Ting DSJ, Elangovan K, et al Large language models in medicine. Nat Med. 2023;29:1930-1940. 10.1038/s41591-023-02448-8 37460753

[2] Achiam J , Adler S, Agarwal S, et al; OpenAI. GPT-4 Technical Report. 2024. 10.48550/arXiv.2303.08774

[3] Iqbal J , Cortés Jaimes DC, Makineni P, et al Reimagining healthcare: unleashing the power of artificial intelligence in medicine. Cureus. 2023;15:e44658. 10.7759/cureus.44658 37799217 PMC10549955

[4] Dwivedi YK , Kshetri N, Hughes L, et al Opinion paper: “So what if ChatGPT wrote it?” Multidisciplinary perspectives on opportunities, challenges and implications of generative conversational AI for research, practice and policy. Int J Inf Manag. 2023;71:102642. 10.1016/j.ijinfomgt.2023.102642

[5] Meng X , Yan X, Zhang K, et al The application of large language models in medicine: a scoping review. iScience. 2024;27:109713. 10.1016/j.isci.2024.109713 38746668 PMC11091685

[6] Gandhi TK , Classen D, Sinsky CA, et al How can artificial intelligence decrease cognitive and work burden for front line practitioners? JAMIA Open. 2023;6:ooad079. 10.1093/jamiaopen/ooad079 37655124 PMC10466077

[7] Wen B , Norel R, Liu J, et al Leveraging large language models for patient engagement: the power of conversational AI in digital health. arXiv, 2024. https://arxiv.org/html/2406.13659v1

[8] Ullah E , Parwani A, Baig MM, et al Challenges and barriers of using large language models (LLM) such as ChatGPT for diagnostic medicine with a focus on digital pathology—a recent scoping review. Diagn Pathol. 2024;19:43. 10.1186/s13000-024-01464-7 38414074 PMC10898121

[9] Decker H , Trang K, Ramirez J, et al Large language model-based chatbot vs surgeon-generated informed consent documentation for common procedures. JAMA Netw Open. 2023;6:e2336997. 10.1001/jamanetworkopen.2023.36997 37812419 PMC10562939

[10] Lim ZW , Pushpanathan K, Yew SME, et al Benchmarking large language models’ performances for myopia care: a comparative analysis of ChatGPT-3.5, ChatGPT-4.0, and Google Bard. eBioMedicine. 2023;95:104770. 10.1016/j.ebiom.2023.104770 37625267 PMC10470220

[11] Ayers JW , Poliak A, Dredze M, et al Comparing physician and artificial intelligence chatbot responses to patient questions posted to a public social media forum. JAMA Intern Med. 2023;183:589-596. 10.1001/jamainternmed.2023.1838 37115527 PMC10148230

[12] Bernstein IA , Zhang Y, Govil D, et al Comparison of ophthalmologist and large language model chatbot responses to online patient eye care questions. JAMA Netw Open. 2023;6:e2330320. 10.1001/jamanetworkopen.2023.30320 37606922 PMC10445188

[13] He W , Zhang W, Jin Y, et al Physician versus large language model chatbot responses to web-based questions from autistic patients in Chinese: cross-sectional comparative analysis. J Med Internet Res. 2024;26:e54706. 10.2196/54706 38687566 PMC11094593

[14] Walter A-L , Baty F, Rassouli F, et al Diagnostic precision and identification of rare diseases is dependent on distance of residence relative to tertiary medical facilities. Orphanet J Rare Dis. 2021;16:131. 10.1186/s13023-021-01769-6 33745447 PMC7983389

[15] Adachi T , El-Hattab AW, Jain R, et al Enhancing equitable access to rare disease diagnosis and treatment around the world: a review of evidence, policies, and challenges. Int J Environ Res Public Health. 2023;20:4732. 10.3390/ijerph20064732 36981643 PMC10049067

[16] Exabo. Accessed September 27, 2024. https://exabo-lung.mig-frankfurt.de/

[17] Walther D , Steinmann O, Schaefer J, et al Conception of an expert advisory board for the European Reference Network for rare respiratory diseases. Stud Health Technol Inform. 2018;247:236-240. 29677958

[18] HOME. ERN-LUNG rare respiratory disease. Accessed October 18, 2024. https://ern-lung.eu/

[19] Weber MT , Schaaf J, Storf H, et al Editing physicians’ responses using GPT-4 for academic research. In: dHealth 2024. IOS Press; 2024:101-6. 10.3233/SHTI240019 38682512

[20] Labrak Y , Bazoge A, Morin E, et al BioMistral: a collection of open-source pretrained large language models for medical domains. 2024. 10.48550/arXiv.2402.10373

[21] Creating a GPT. OpenAI Help Center. 2024. Accessed October 16, 2024. https://help.openai.com/en/articles/8554397-creating-a-gpt

[22] Orphanet. Knowledge on rare diseases and orphan drugs. Accessed September 27, 2024. https://www.orpha.net/

[23] Hu EJ , Shen Y, Wallis P, et al 2021. LoRA: low-rank adaptation of large language models. arXiv, 2021. 10.48550/arXiv.2106.09685

[24] oobabooga. oobabooga/text-generation-webui. 2024.

[25] Mays N , Pope C. Rigour and qualitative research. BMJ. 1995;311:109-112. 10.1136/bmj.311.6997.109 7613363 PMC2550154

[26] Caley MJ , O’Leary RA, Fisher R, et al What is an expert? A systems perspective on expertise. Ecol Evol. 2014;4:231-242. 10.1002/ece3.926 24558579 PMC3925425

[27] SurveyMonkey: the world’s most popular survey platform. SurveyMonkey. Accessed October 16, 2024. https://www.surveymonkey.com/

[28] Joshi A , Kale S, Chandel S, et al Likert scale: explored and explained. Br J Appl Sci Technol. 2015;7:396-403. 10.9734/BJAST/2015/14975

[29] South L , Saffo D, Vitek O, et al Effective use of Likert scales in visualization evaluations: a systematic review. Comput Graph Forum. 2022;41:43-55. 10.1111/cgf.14521

[30] Kruskal WH , Wallis WA. Use of ranks in one-criterion variance analysis. J Am Stat Assoc. 1952;47:583-621. 10.1080/01621459.1952.10483441

[31] Koo TK , Li MY. A guideline of selecting and reporting intraclass correlation coefficients for reliability research. J Chiropr Med. 2016;15:155-163. 10.1016/j.jcm.2016.02.012 27330520 PMC4913118

[32] IBM SPSS Statistics. Accessed October 30, 2024. https://www.ibm.com/products/spss-statistics

[33] Welcome to Python.org. Python.org. Accessed October 30, 2024.https://www.python.org/

[34] Statistical functions (scipy.stats)—SciPy v1.14.1 manual. Accessed October 30, 2024. https://docs.scipy.org/doc/scipy/reference/stats.html

[35] scikit-posthocs–scikit-posthocs 0.7.0 documentation. Accessed October 30, 2024. https://scikit-posthocs.readthedocs.io/en/latest/

[36] Sharkiya SH. Quality communication can improve patient-centred health outcomes among older patients: a rapid review. BMC Health Serv Res. 2023;23:886. 10.1186/s12913-023-09869-8 37608376 PMC10464255

[37] Auschra C , Möller J, Berthod O, et al Communicating test results in a comprehensible manner: a randomized controlled trial of word usage in doctor-patient communication. Z Evid Fortbild Qual Gesundhwes. 2020;156-157:40-49. 10.1016/j.zefq.2020.07.007 32900672

[38] King A , Hoppe RB. “Best practice” for patient-centered communication: a narrative review. J Grad Med Educ. 2013;5:385-393. 10.4300/JGME-D-13-00072.1 24404300 PMC3771166

[39] Moudatsou M , Stavropoulou A, Philalithis A, et al The role of empathy in health and social care professionals. Healthcare (Basel). 2020;8:26. 10.3390/healthcare8010026 32019104 PMC7151200

[40] Paul M , Maglaras L, Ferrag MA, et al Digitization of healthcare sector: a study on privacy and security concerns. ICT Express. 2023;9:571-588. 10.1016/j.icte.2023.02.007

[41] Minaee S , Mikolov T, Nikzad N, et al Large language models: a survey. 2024. 10.48550/arXiv.2402.06196

[42] The open-source advantage in large language models (LLMs). Accessed January 30, 2025. https://arxiv.org/html/2412.12004

[43] Williams S , Huckle J. Easy problems that LLMs get wrong. 2024. 10.48550/arXiv.2405.19616

[44] Bélisle-Pipon J-C. Why we need to be careful with LLMs in medicine. Front Med (Lausanne). 2024;11:1495582. 10.3389/fmed.2024.1495582 39697212 PMC11652181

[45] Wu DC , Zhao X, Wu J. Online physician–patient interaction and patient satisfaction: empirical study of the internet hospital service. J Med Internet Res. 2023;25:e39089. 10.2196/39089 37616031 PMC10485723

[46] Lewis P , Perez E, Piktus A, et al Retrieval-augmented generation for knowledge-intensive NLP tasks. 2021. 10.48550/arXiv.2005.11401

[47] Gao Y , Xiong Y, Gao X, et al Retrieval-augmented generation for large language models: a survey. 2024. 10.48550/arXiv.2312.10997

