# Supplementary material for: MedBot vs RealDoc: efficacy of large language modeling in physician-patient communication for rare diseases
Source: J Am Med Inform Assoc. 2025 Feb 25;32(5):775–83. doi: 10.1093/jamia/ocaf034 (PMC12012358; doi:10.1093/jamia/ocaf034)
Supplement: ocaf034_Supplementary_Data [file ocaf034_supplementary_data.zip › ocaf034_Supplementary_Data/Supplementary_Material_A.docx]

| **Patient Query** | **Physician** | **BioMistral 7B** | **GPT 4.0** |
| --- | --- | --- | --- |
| Patientin ist 26 Jahre alt.  2016 Erythema nodosum Unterschenkel bds parainfektiös.  01.2917 Larynxsarkoidose(Univ.Klinik Innsbruck) Initial mit systemischer Glucocorticoidtherapie bis Nov.2018. Völlige Normalisierung der Ary und der Epiglottis war auch nach der Ersttherpaie nicht erreicht. Rezidiv Dez. 2019. Seitdem wieder systemische Glucocorticoidtherapie meinerseits absteigend zur Zeit bei Prednison 2,5mg/d. Wiel lange soll/kann die Thertapiedauer sein? Alternativen auch mit Hinsicht auf eine mögliche gewünschte SS in nächster Zeit. Herzlichen Dank | Aus der umfangreichen Erfahrung in der Behandlung der Sarkoidose (anderer Lokalisation) kann man sagen, dass bei einem Rezidiv, wenn denn die Ersttherapie die übliche Mindestdauer von ca. 6 Monaten eingehalten hatte, die Therapiedauer dann eher länger erfolgen sollte (z.B. 9 bis 12 Monate). Allerdings ist es ja zu hinterfragen, ob bei einer Dosis von 2,5 mg noch ein systemischer Effekt zu erwarten ist. Deshalb kann ich dann nur raten - solche Dosen setzen wir beim Erwachsenen nur in der Phase des Therapie-Ausschleichens ein - dann auch den nächste Schritt zu gehen, und die Therapie zu beenden. Wenn tatsächlich initial fast zwei Jahre wirksam (>5mg) behandelt wurde, kann man bei stabilem Krankheitsbild natürlich auch pragmatisch sein und die jetzige Dosis beibehalten, die ja auch unter der Dosis einer KI für einen SS ist. Beste Grüße. | Die Therapiedauer ist schwierig festzulegen. Entweder man kann in den letzten Monaten mit einem niedrigen Dosier, und es ist unwahrscheinlich, dass es im nächsten Jahr erneut zu einem schweren Exazerbation kommt, oder man kann die Therapie pausieren und eine Therapiepause von 3 Monaten nach der Entzündungsstillstellung verstehen. Es gibt auch keinen eindeutigen Beweis, dass eine lange Therapie eine Folgeerkrankungen hervorruft. | Die Therapie der Larynxsarkoidose mit Glucocorticoiden kann oft langwierig sein, insbesondere bei wiederkehrenden Symptomen. Es ist wichtig, die Prednison-Dosis so gering wie möglich zu halten, um Nebenwirkungen zu minimieren. Da Sie eine Schwangerschaft planen, sollten Alternativen wie Methotrexat oder Azathioprin in Erwägung gezogen werden, aber diese müssen vor einer Schwangerschaft abgesetzt werden. Sprechen Sie ausführlich mit Ihrem behandelnden Arzt, um einen individuellen Plan zu erstellen, der sowohl Ihre Gesundheit als auch Ihre Familienplanung berücksichtigt. |
| Hallo,  ich bin seit 2 Jahren an Neurosarkoidose erkrankt, keine weiteren Organe beteiligt. Seit dem wird als Therapie täglich 200 mg Azathioprin und 5 mg Cortison eingesetzt.  Die Raumforderung im Gehirn hat sich seit dem erheblich zurückgebildet, keine Rezidive und auch allmähliche Besserung des Allgemeinbefindens (Fatigue, Kopfschmerzen, Tinnitus, Schwäche weiterhin vorhanden)  Vielleicht könnten Sie mir bitte bei folgenden Fragen helfen:  1.) Wie sieht erfahrungsgemäß eine Therapie der Neurosarkoidose unter Azazthioprin aus ?   2.) Wie gelingen perspektivisch sichere Reduzierungsversuche der Medikamente ? Der il2-Rezeptor war bei Ausbruch der Krankheit 4fach erhöht, aktuell unter den Medikamenten unauffällig im Rahmen. ACE immer unauffällig. Ist der il2-Rezeptor eine zuverlässige Laborgrösse zur Überwachung der Krankheitsaktivität auch bei Neurosarkoidose ? Welche weiteren spezifischen Laborwerte gibt es noch ?   3.) Könnten Sie mir eventuell Ansprechpartner für speziell Neurosarkoidose (ggf. in Sachsen) nennen ? Gibt es spezialisierte Behandlungszentren ? | 1.) Falls der Therapie-Erfolg im Verlauf sich bestätigt, wird Azathioprin langsam reduziert (über Monate) und dann abgesetzt. Das gleiche gilt für Prednisolon. 2.) Die Laborwerte (Sarkoidose Biomarkers) werden für eine Therapie Entscheidung nur teilweise berücksichtig. Wichtiger sind die Bildgebung und der Zustand der Patienten. Bitte die Biomarker Werte nicht überschätzen oder überinterpretieren. 3.) Leider ist mir kein Spezialist für Neurologie und Sarkoidose im Sachsen bekannt. Wenden Sie sich Bitte an die deutsche Sarkoidose Vereinigung, dort gibt es Listen von Spezialisten Deutschlandweit. | Es gibt derzeit keine geeignete Studie, welche die Wirksamkeit der Therapie bei Neurosarkoidose im Vergleich zur Standardtherapie untersucht hat. Es werden jedoch Berichte von einer positiven Wirkung von Azathioprin bei Neurosarkoidose gemeldet. Aufgrund der begrenzten Evidenz über die Wirksamkeit von Azathioprin bei Neurosarkoidose sind zusätzliche Untersuchungen erforderlich.  2. Eine Reduktion der Azathioprin-Dosis kann sicher sein, wenn kein Rezidiv auftritt. Ein Test auf Erhöhung des il2-Rezeptors kann verwendet werden, um ein Rezidiv vorherzusagen, aber ist keine exakte Grösse und ist nicht immer verfügbar. In der Praxis gibt es andere spezifische Laborkontrolle, wie Tumornachweis oder Erhöhung der Neutrophilen.  3. In Deutschland gibt es spezialisierte Zentren zur Behandlung von Sarkoidose, die in der Regel auch die Neuroform behandeln. Es gibt jedoch nur wenige Behandler in Sachsen. Die folgenden Zentren in Deutschland empfahlen sich: Berliner Charité, Münchener Lungenklinik, Universität München, Kölnische Univ.-Frei-Universität und Ruprecht Karls Universität Heidelberg. | Die Therapie der Neurosarkoidose mit Azathioprin und Cortison ist ein üblicher Ansatz, bei dem Azathioprin als Immunsuppressivum wirkt und Cortison Entzündungen reduziert. Eine schrittweise Reduktion der Medikamente ist möglich, sollte aber unter engmaschiger ärztlicher Überwachung erfolgen. Der IL-2-Rezeptor kann als Marker zur Überwachung der Krankheitsaktivität hilfreich sein, jedoch sind auch andere Laborwerte wie ACE, sIL-2R und Lysozym relevant. Für spezialisierte Zentren und Ansprechpartner in Sachsen empfehle ich, Kontakt mit der Universitätsklinik Leipzig oder Dresden aufzunehmen, da diese oft spezialisierte Abteilungen für seltene Krankheiten haben. |
| I was diagnosed with sarcoidosis in 2020 after suffering for about 10 years with symptoms. I was treated with steroids and methotrexate for a year and a half, but every time I reduced my steroid dose my symptoms returned. I was subsequently treated with Infliximab and that completely got rid of my symptoms and improved my x-ray results. I moved to France in August 2022 and the doctors here say that while I still have sarcoidosis, it's not severe enough to treat. I still have skin lesions, extreme fatigue, brain fog, tinnitus, and some respiratory symptoms without treatment. I went from a 90% average as a student to failing my most recent semester, and I can barely make a living because of my current state. The doctors here told me to do some yoga. I would like to know if there are any treatment options that I can explore that are a middle ground between no treatment at all and Infliximab. | According to the recent guideline on sarcoidosis management and treatment, indications for treatment are symptoms affecting quality of life, functional impairment and risk of permanent disability or death. Rehabilitation for fatigue is a very helpful treatment option. Hydroxychloroquine, an anti-malarial drug, can improve skin lesions. It is suggested to be followed at a reference center for sarcoidosis, where all these aspects and alternative treatment options should be considered. Best regards | I'm sorry to hear about your suffering, and I hope the rest of this answer is helpful. The good news is that there are some additional treatment options that you may want to discuss with your rheumatologist. I will discuss several treatment options in this response that you can bring up with them during your next visit. The first one is infliximab, which is an effective biologic therapy that targets TNF-alpha. As you mentioned, infliximab was effective in treating your sarcoidosis symptoms, and it may be an option to explore if the side effects are too severe for you to tolerate. However, it may also be worth exploring some other similar biologic therapies. For example, adalimumab and certolizumab pegol are both TNF-alpha inhibitors that may be effective for treating your sarcoidosis. Another option is rituximab, an immunosuppressive monoclonal antibody that targets CD20 on B cells and has been effective in treating other autoimmune diseases, including sarcoidosis. I would suggest discussing the specific risks and side effects of each of these options with your rheumatologist, and determine which one is the best option for you. Another option to explore is a corticosteroid-sparing treatment, such as methotrexate. Methotrexate is a drug that inhibits the folate cycle and has been shown to be effective in treating various autoimmune diseases, including rheumatoid arthritis and sarcoidosis. It can also be effective in treating sarcoidosis as a corticosteroid-sparing agent, but it may cause liver damage, so it is important to monitor your liver function closely if you decide to take it. Again, it is important to discuss the specific risks and side effects of methotrexate with your rheumatologist to determine if it is the right treatment option for you. If you are not interested in any of these pharmacological options, there are also some non-pharmacological options that you may want to explore. These include physiotherapeutic exercises, yoga, and relaxation techniques. These approaches may not directly target the underlying cause of your symptoms, but they may help alleviate some of the symptoms and improve your overall well-being. | I'm sorry to hear about the challenges you're facing. Given your history and current symptoms, it might be worth discussing with your doctors the possibility of a low-dose maintenance therapy, perhaps using methotrexate or hydroxychloroquine, which can sometimes help manage sarcoidosis symptoms with fewer side effects than stronger medications like Infliximab. Additionally, considering treatments that address specific symptoms, such as corticosteroid creams for skin lesions or medications for fatigue, might provide some relief. It's crucial to work closely with a sarcoidosis specialist to tailor a treatment plan that balances efficacy with tolerability. |
| Bei Osteoporose ist Vit D entsprechend dem Spiegel zu supplementieren, bei Sarkoidose soll kein Vit D zugeführt werden.  Spiegelbestimmung des aktiven Vit D ist auch für entsprechende Fachärzte schwierig. Die Interpretation des Spiegels konnte dann vom Facharzt auch nicht aussagekräftig vorgenommen werden. | Bei Patienten mit Sarkoidose sollte man beide Vitamin D-Werte (aktiv UND inaktiv) im Serum messen lassen. Nur wenn beide Werte niedrig sind, kann eine Substitutionstherapie mit DEKRISTOL eingeleitet werden. Dass die Spiegelbestimmung schwierig sei, kann ich nicht bestätigen. Bei Sarkoidose muss keine Calcium-Zufuhr zusätzlich zu Vitamin D erfolgen, das ist eher nachteilig, aber Vitamin D allein (wenn beide Werte im Serum niedrig sind, siehe oben) ist kein Problem. | Ein Spiegelbestimmung ist nicht erforderlich, aber ein Vitamin D3 Supplement bei Sarkoidose sollte vermieden werden. Die Messung eines Vitamin D3 Spiegels ist für den Facharzt schwer zu interpretieren. | Bei Osteoporose ist die Supplementierung von Vitamin D wichtig, um die Knochengesundheit zu unterstützen, wobei die Dosierung anhand des aktuellen Spiegels angepasst werden sollte. Bei Sarkoidose hingegen kann eine Vitamin-D-Supplementierung schädlich sein, da diese Krankheit oft mit erhöhten Kalziumspiegeln verbunden ist. Die Bestimmung und Interpretation des aktiven Vitamin-D-Spiegels ist komplex und selbst für Fachärzte manchmal schwierig, weshalb es oft sinnvoll ist, die Gesamtbildgebung und klinischen Symptome in die Entscheidung einzubeziehen. Eine enge Zusammenarbeit mit einem Endokrinologen oder Rheumatologen kann hier hilfreich sein, um eine genaue Diagnose und geeignete Behandlung sicherzustellen. |
| Ist eine 6 wöchige oder eine 12 wöchige Kortisontherapie beim Löfgren Syndrom zielführender, wenn ich schon nach einer Woche eine komplette Heilung der Hautverfärbung und Arthritis spüre? | Beim Löfgren Syndrom ist in der Norm keine Kortisontherapie notwendig, sondern es wird eine antientzündliche Therapie empfohlen. Wenn aber eine Kortisontherapie schon eingeleitet wurde, sollte diese mindestens 6 Monate auch mit niedriger Dosis fortgesetzt werden. Bei frühzeitigem Absetzen kann ein erneuter Ausbruch der Symptome auftreten. | In diesem Fall wäre eine 6‑wöchige Kortisontherapie mit einer Gesammdosis von 20 mg/Tag die beste Behandlungsmethode. Eine 12-wöchige Behandlung wäre nicht indiziert, da kein Vorteil durch eine längere Therapiezeit erreicht werden würde. | Beim Löfgren-Syndrom, das eine akute Form der Sarkoidose darstellt, kann eine kurzfristige Kortisontherapie von sechs Wochen oft ausreichend sein, insbesondere wenn Sie bereits nach einer Woche eine deutliche Besserung der Symptome bemerken. Eine längere Behandlung von zwölf Wochen könnte in Erwägung gezogen werden, wenn die Symptome zurückkehren oder nicht vollständig verschwinden. Es ist wichtig, dies mit Ihrem Arzt zu besprechen, um die individuell beste Behandlungsdauer festzulegen und mögliche Nebenwirkungen zu minimieren. |
| Ist ein Booster mit Biontech sinvoll oder ratsam wenn man vor einem Monat an einer Akutform (Löfgren-Syndrom) der Sarkoidose erkrankt ist und die Krankheit die Lungen unbemerkt befallen hat? Sollte man warten bis die akute Aktivität des Immunsystems, das offensichtlich gegen eigene Organe kämpft, sich wieder etwas beruhigt hat? Kann ein Booster in der Akutform ein Trigger für eine chronische Sarkoidose sein oder gar für einen unerwünschten Effekt der Impfung (Cytokin Sturm)? | Bisher gibt es solche Hinweise nicht und alle Befürchtungen der Patienten hinsichtlich einer Verschlimmerung der Sarkoidose durch eine Impfung und/oder Booster haben sich nicht bewahrheitet, sodass eine uneingeschränkte Impf- bzw. Boosterempfehlung bei chronisch entzündlichen Erkrankungen und somit auch bei der Sarkoidose besteht. Einen guten Überblick zu dieser Thematik finden Sie bei der Deutschen Sakoidosevereinigung. Beste Grüße. | Das hängt davon ab, welche Impfungen Sie bereits erhalten haben. | In Ihrem speziellen Fall ist es ratsam, vor einer Booster-Impfung mit Ihrem behandelnden Arzt zu sprechen, insbesondere da Sie kürzlich an einer akuten Form des Löfgren-Syndroms erkrankt sind. Ihr Arzt kann Ihre individuelle Situation am besten einschätzen, einschließlich der aktuellen Aktivität Ihres Immunsystems und des Zustands Ihrer Lungen. Eine Impfung könnte theoretisch das Immunsystem zusätzlich belasten, daher ist eine sorgfältige Abwägung wichtig. Es gibt keine klaren Beweise dafür, dass eine Booster-Impfung eine chronische Sarkoidose auslösen oder einen Zytokinsturm verursachen würde, aber eine ärztliche Einschätzung ist entscheidend. Ihr Arzt kann Ihnen auch alternative Zeitpunkte für die Impfung empfehlen, wenn dies als sicherer angesehen wird. |
| Lymphopenie wird in Zusammenhang mit der Covid19 Erkrankung als früher Biomarker für einen potentiell schweren Verlauf genannt. Bedeutet dies auch im Umkehrschluss, dass ein Mangel an Lymphozyten aufgrund einer bestehenden Sarkoidose (Typ1) ein besonderes Risiko für einen schweren Verlauf darstellt?  Danke! | Bisher sind Berichte über schwere Verläufe bei Sarkoidsepatienten glücklicherweise die Ausnahme. Eine stabile Situation ist nicht mit einem erhöhten Risiko verbunden, eher die immunsuppressive Therapie. Die Sarkoidose-typische Lymphopenie selbst scheint nicht ursächlich für einen schweren Verlauf zu sein. Einen guten Überblick finden Sie bei der Deutschen Sakoidosevereinigung. Beste Grüße. | In der Regel können wir annehmen, dass Lymphopenie die Aktivität des Sarkoidoses im Blut reflektiert. Es ist bekannt, dass Patienten mit Lymphopenie bei Sarkoidose ein erhöhtes Risiko für eine schwere Krankheitsverlaufsform haben . Es ist auch bekannt, dass Patienten mit Sarkoidose und COVID-19 eine erhöhte Mortalität haben . Daher könnte der Mangel an Lymphozyten im Blut ein Indikator dafür sein, dass COVID-19 mit Sarkoidose kombiniert ist und ein schwerer Verlauf nimmt. Es ist aktuell noch unbekannt, ob Sarkoidose ein Risikofaktor für den schweren Krankheitsverlauf bei COVID-19 ist und ob ein Therapieansatz auf den Sarkoidose-Abschnitt wirkt oder nicht . | Ein Lymphozytenmangel kann generell das Immunsystem schwächen und theoretisch das Risiko für schwerere Verläufe von Infektionen, einschließlich Covid-19, erhöhen. Bei Sarkoidose Typ 1 kann eine Lymphopenie vorliegen, was potenziell ein höheres Risiko für schwere Verläufe von Covid-19 bedeuten könnte. Es ist jedoch wichtig, dies individuell zu bewerten und mit Ihrem behandelnden Arzt zu besprechen, da viele Faktoren die Krankheitsverläufe beeinflussen können. |
| Ich bin an Sarkoidose erkrankt (Lunge, Herz, Multiorgan).  Aufgrund der cardialen Beteiligung werde ich über Jahre Cortison einnehmen. Ich vertrage das Medikament sehr gut und habe keine spürbaren Nebenwirkungen.  Ich bin nach drei Jahren Einnahme auf 5 mg Prednisolon gesenkt.  Meine sehr gute, erfahrene Ärztin meint, ich könnte den Schritt auf 4mg versuchen, ich könnte aber auch auf 5mg bleiben. Die Entscheidung läge bei mir.  Ich traue mich nicht von 5mg auf 4mg zu senken.  MEINE FRAGE  Ist das 1mg weniger in Relation zum Risiko des neu Aufflammens der Sarkoidose relevant?  Ist 1mg (4mg statt jetzt 5mg) weniger in einer Langzeiteinnahe relevant?  Mann / 48 Jahre / 80 kg.  Vielen Dank ! | Da es keine Daten zu Ihrer Frage gibt, kann ich gut nachvollziehen, dass Ihre betreuende Ärztin das ganz Ihnen überlässt, das zu entscheiden. Sowohl 5 als auch 4 mg Prednisolon liegen unterhalb des Radars. Stellen Sie sich vor, ein Erreger ist zu klein, um im Mikroskop erkennbar zu sein – da macht es dann keinen Unterschied, ob er ein bisschen kleiner oder größer ist – zu klein ist er in jedem Fall. In so einer Situation befinden Sie sich offensichtlich mit der Steroiddosis.  Wurde jemals ein Auslassversuch gemacht? Wenn dies nicht der Fall ist, könnte man ja darüber nachdenken, und dann wäre die weitere Reduktion in kleinen Schritten ein möglicher Weg. Viele Krankheitsverläufe befinden sich nach langer Therapie im Ruhezustand und man kann wohl uneingeschränkt festhalten, dass eine überflüssige Therapie immer eine schlechte Therapie ist. Ob man einen solchen Auslass- oder Absetzversuch unternehmen will, hängt natürlich sehr vom bisherigen Verlauf – frühere Auslassversuche? – ab. Mit den besten Wünschen. | 1 mg Prednisolon in einer langen Einnahme ist mit einem niedrigeren Risiko verbunden. Wenn es nicht zu einer Verschlechterung der Erkrankung kommt, kann es in der Regel gut sein. Wenn jedoch ein neu Aufflammen auftritt, kann es schwer sein, die Krankheit zu behandeln. Es ist daher wichtig, die Entscheidung auf Grundlage einer individuellen Abwägung der Risiken und Vorteile zu treffen. | Ein Unterschied von 1 mg Prednisolon, insbesondere bei einer so niedrigen Dosis wie 5 mg, ist in der Regel nicht sehr groß. Es ist verständlich, dass Sie sich Sorgen machen, aber das Risiko eines erneuten Aufflammens der Sarkoidose ist bei einer langsamen und schrittweisen Reduzierung gering. Ihr Körper hat sich bereits an eine niedrigere Dosis angepasst, und eine Reduktion um 1 mg ist oft gut verträglich. Trotzdem sollten Sie die Entscheidung gemeinsam mit Ihrer Ärztin treffen und bei jeglichen Anzeichen einer Verschlechterung sofort mit ihr sprechen. Vertrauen Sie auf Ihre ärztliche Betreuung und hören Sie auf Ihren Körper. |
| Soll man sich gegen Covid 19 impfen lassen, wenn man Sarkoidose  hat? | Da es sich bei der Sarkoidose um eine seltene Erkrankung handelt, gibt es verständlicherweise noch keine spezifische Leitlinie dazu. Grundsätzlich gelten bei jeder Erkrankung die normalen Indikationsregeln. Es kann jedoch zwei besondere Gründe geben, warum eine Impfung bei Sarkoidose in Betracht gezogen werden sollte: Zum einen kann eine stark eingeschränkte Lungenfunktion eine dringlichere Impfindikation darstellen, und zum anderen könnte eine immunsuppressive Therapie der Sarkoidose eine Indikation für eine Impfung sein. Die Sarkoidose selbst stellt jedoch keine Kontraindikation für eine solche Impfung dar. | Wenn Sie eine Sarkoidose haben, sollten Sie vor der COVID-19-Impfung mit dem Impfzentrum sprechen. Die COVID-19-Impfung ist in der Regel sicher. Das Risiko einer schweren Reaktion auf die Impfung ist nicht höher als bei einer Impfung gegen Influenza. Das Risiko einer schweren Reaktion ist im Vergleich zur COVID-19-Erkrankung niedriger. Sie sollten mit dem Arzt sprechen, bevor Sie impfen lassen. | Ja, es ist generell ratsam, sich gegen COVID-19 impfen zu lassen, auch wenn man Sarkoidose hat. Menschen mit chronischen Erkrankungen wie Sarkoidose haben ein höheres Risiko für schwere Verläufe von COVID-19. Besprechen Sie jedoch unbedingt Ihre individuelle Situation mit Ihrem behandelnden Arzt, da es spezifische medizinische Faktoren geben könnte, die berücksichtigt werden müssen. Ihr Arzt kann Ihnen die bestmögliche Empfehlung basierend auf Ihrem Gesundheitszustand geben. |
| Where the patient can consult? She has a diagnosis of sarcoidosis in a mediastinal lymph node 2 years ago, stable, without any treatment. Also held in 2011 a surgery for hemangioma of the tongue, with scar process progressive since that time. Now the tongue biopsy showed granulomatous reaction with foreign bodies giant cells with 2 different pathology reports. (Sarcoidosis vs. granulomatous reaction investigate other cause). | Dear patient, tongue and oral involvement in sarcoidosis can lead to dysfunction in speaking and eating with reduction in quality of life (I have a couple of patients with this rare manifestation), i.e. treatment is needed asap. If you share the information about the country of origin, I can suggest a specific sarcoidosis center and provide the contact of the Specialist. Best wishes, | If the patient is currently symptomatic, I would recommend the patient consults with an Ear Nose Throat doctor. Also, it would be a good idea for the patient to see a pulmonologist and a gastroenterologist for the sarcoidosis and to see a dermatologist for the tongue lesion. If the patient has not seen these specialists before, it would be important for the patient to provide them with a copy of the pathology report and the diagnoses that have been made. | Given the complexity of her medical history, the patient should consult a specialist in sarcoidosis, such as a pulmonologist or a rheumatologist, for further evaluation of her condition. Additionally, considering the tongue biopsy results, it would be beneficial to see an oral and maxillofacial pathologist or an otolaryngologist for a thorough assessment and to explore other potential causes of the granulomatous reaction. Coordinated care among these specialists will help ensure an accurate diagnosis and appropriate management plan. |
| Innerhalb von 3 Moaten ACE Wert zum 2. Mal von 80,6, CT unauffällig, fühle mich aber schnell erschöpft und spüre Enge im Halsbereich, leichter Schnupfen, leichte Kopfschmerzen, leichter Husten mit Schleim, bin Psoriasispatientin, nehme Humira 40 alle 14 Tage, Allergie gegen ASS, vor 4 Wochen noch unspezifische Schmerzen an Beinen und Armen  Habe ich definitiv Morbus Böck?  Welche weiteren Untersuchungen sollten folgen? | Der ACE-Wert alleine hat keine diagnoseweisende Bedeutung. Er kann auch bei anderen knötchenbildenden (granulomatösen) Erkrankungen erhöht sein. Erhöhte Werte gibt es auch bei M. Gaucher, Hyperthyreose, Diabetes mellitus (mit Retinopathie), Leberzirrhose, Silikose, Berylliose, HIV-Infektion, MCTD, chronischem Müdigkeitssyndrom, in der Schwangerschaft usw. Das bedeutet aber nicht, dass man jetzt alle diese Krankheiten ausschließen muss, es bedeutet nur, dass der Messwert alleine keine Diagnose erlaubt. Beste Grüße. | Husten, leichte Schwellung im Halsbereich, Kopfschmerzen sind Hinweise auf eine Sinusitis. Wir empfehlen, dass Sie mit Ihrem behandelnden Arzt besprechen sollten, um weiteres Vorgehen zu erwähnen. Ihr Arzt kann eine Referenz für eine weiterführende Diagnostik erwähnen, d. h. er könnte Sie zu einer weiterführenden Diagnostik verweisen. | Ein erhöhter ACE-Wert kann auf verschiedene Erkrankungen hinweisen, darunter auch Morbus Boeck (Sarkoidose), aber ist nicht allein diagnostisch. Da Ihr CT unauffällig ist und Sie mehrere Symptome haben, wäre es wichtig, weitere Untersuchungen durchzuführen. Dazu könnten ein Lungenfunktionstest, eine Bronchoskopie mit Biopsie und spezifische Bluttests gehören. Besprechen Sie diese Symptome und die möglichen nächsten Schritte detailliert mit Ihrem behandelnden Arzt, um eine präzise Diagnose zu erhalten. |
| Patient männlich, 60J.  Sarkoidose seit 15J bekannt, aber laut Röntgen und LuFu in Remission.  Patient berichtet über morgentliche Atemgemgeräusche (vorzugsweise beim Einatmen), die an zerplatzenden Badeschaum erinnern und er demonstriert auch eine Aufioaufnahme am Handy. Der Patient hat sichtlich trockene Gesichtshaut, trockene Schleimhaut im HNO-.Bereich ist bekannt, Augentropfen werden verwendet. Der Patient meint, die Intensität des sauren Geschmacks von Schleim würde meist mit langdauernden Müdigkeitsphasen korrelieren, die morgentlichen Atemgeräusche würden mit noch mehr Müdigkeit korrelieren.  Ist eine Abklärung notwendig und falls ja, was ist die empfohlene Vorgangsweise (Differentialdiagnosen?)? | Der Patient sollte am besten eine komplette Lungenfunktionsdiagnostik bekommen. Eine obstruktive Ventilationsstörung kann sich nach Remission der Sarkoidose entwickeln und persistent sein. Eine adäquate Therapie mit Bronchodilatoren sollte rechtzeitig eingeleitet werden. Eine Abklärung wegen Sjögren Syndrom beim Rheumatologe sollte ebenfalls erfolgen. Beste Grüße. | Es ist empfohlen, einen internistischen Workup durchzuführen, um weitere Differentialdiagnosen auszuschließen. | Ja, eine Abklärung ist notwendig. Die morgendlichen Atemgeräusche könnten auf eine wiederkehrende oder neue Atemwegsproblematik hinweisen, trotz der bisherigen Remission der Sarkoidose. Differentialdiagnosen könnten eine rezidivierende Sarkoidose, eine bronchiale Hyperreagibilität, oder eine interstitielle Lungenerkrankung umfassen. Eine erneute Lungenfunktionsprüfung, hochauflösende CT der Lunge und möglicherweise eine Bronchoskopie wären sinnvoll, um die Ursache der Symptome genauer zu bestimmen. Zudem sollte die anhaltende Trockenheit der Schleimhäute und der damit verbundene saure Geschmack weiter untersucht werden, um andere systemische Erkrankungen auszuschließen. |
| Hallo,  bin 55, weiblich, Nichtraucherin. Bei mir ist es fast sicher (warte auf den entscheidenden Termin zur endgültigen Diagnosestellung beim Pneumologen – am 1.7.!), dass ich an Sarkoidose der Lunge, Lymphknoten und wirklich massiver Knochenbeteiligung (vor allem Becken aber auch LWS und Femur) festgestellt worden. Maligne Erkrankungen wurden ausgeschlossen. Folgende Diagnostik wurde gemacht: PET-CT (Ganzkörper PET-CT), MRT der LWS, Becken und beide Femuren sowie Gehirn, CT Thorax und Abdomen, 2x Bronchoskopie mit BAL mit Lymphkontenbiopsie, Mikrobiologische Tests (war alles ok, auch Tuberkulose wurde ausgeschlossen). Es konnte aber keines der Knötchen in der Lunge biopsiert werden, da die so klein sind. Hämatologische Untersuchung, Tumor-Marker, Knochenmark -Biopsie (Dornfortsatz) waren bezüglich evtl Krebserkrankungen alle im Normbereich. Bei der Biopsie wurden nicht nekrotisierende, epitheloidzellige Granulome gefunden. Bei der Blutuntersuchung wurden in den letzten Monaten/ sogar Jahren die Entzündungsparameter sowie Lymphozyten etwas erhöht, zuletzt auch der ACE Wert. Alkalische Phosphatase, und Kalzium waren im Normbereich. Lungenfunktionstest war auch im Normbereich.  Vergleich von MRT der LWS seit 2019 bis heute hat ergeben, dass die Läsionen (Granulome) sich durch die Zeit vermehrt haben und etwas größer geworden sind (man dachte zuerst, dass wären Hämangiome). Thorax CT vom Oktober 2021 und Februar 2021 ergab keine wesentliche Veränderung (Kleine Knötchen und etwas vergrößerte Lymphknoten in beiden Hili).  Schwierigkeiten mit der Lunge bzw. Atmung oder Husten habe ich nicht. Bin in den letzten Jahren aber sehr Müde, Abgeschlagen und leide auch am Schwitzen (dachte erst, alles liegt nur an der Menopause). Haut- und Augenprobleme habe ich nicht.  Da ich im Ausland wohne, würde ich Ihre Meinung bezüglich der Therapie (und evtl. fehlender Diagnostik) sehr schätzen!   Meine Fragen:  1. Da ich seit langem starke Schmerzen in der LWS und im Becken habe (trotz Metamizol oder Meloxicam), kann ich davon ausgehen, dass die sich mit medikamentöser Therapie der Sarkoidose bessern?  2. Was passiert mit den Granulomen und dem Knochen/Knochemark unter medikamentöser Behandlung mit der Zeit? Bleiben da Löcher im Knochen zurück?  3. Wie sieht die Standardtherapie bei Knochen-Sarkoidose aus? Wird zuerst mit Methylprednisolone behandelt? Mit welcher Dosis fängt man an und wie lange?  Habe nämlich Angst vor heftigen Nebenwirkungen und noch viel mehr vor evtl. anderen Medikamenten (eine Ärztin hat erwähnt, dass ich vielleicht auch mal Zytostatika einnehmen müsste)!  Da sich die ganze Geschichte mit der Diagnostik schon seit September 2021 zieht, bin ich langsam ungeduldig und habe gleichzeitig Angst ohne Medikation bei all den Granulomen in den Knochen (und der Lunge) zu sein, aber gleichzeitig Angst vor Nebenwirkungen dieser Medikamente).  Bitte sie höflich um Ihren Rat bzw. Info bezüglich der Therapie.  Vielen Dank im Voraus! | Die Behandlung der Sarkoidose beginnt bei Patienten, die eine Behandlung benötigen, immer sehr ähnlich: Es werden vom Cortison abgeleitete Medikamente, wie das von Ihnen genannte Methyprednisolon, eingesetzt. Es können auch Medikamente verwendet werden, um die Menge des benötigten Cortisons zu reduzieren, wie zum Beispiel solche aus dem Bereich der Zytostatika, jedoch in sehr niedriger Dosierung. Zu Ihren Fragen im Einzelnen: 1. Da Sie seit langem starke Schmerzen in der LWS und im Becken haben (trotz Metamizol oder Meloxicam), können Sie davon ausgehen, dass sich diese mit medikamentöser Therapie der Sarkoidose verbessern? Typischerweise ja. 2. Was passiert mit den Granulomen und dem Knochen/Knochenmark unter medikamentöser Behandlung mit der Zeit? Bleiben da Löcher im Knochen zurück? Die Löcher werden teilweise kleiner, teilweise verschwinden sie, teilweise bleiben Reste zurück. Typischerweise sind solche Löcher, insbesondere wenn sie die Stabilität nicht beeinträchtigen, eher ohne große Bedeutung. 3. Wie sieht die Standardtherapie bei Knochen-Sarkoidose aus? Wird zuerst mit Methylprednisolon behandelt? Mit welcher Dosis fängt man an und wie lange? Die Therapie beginnt bei der Sarkoidose immer ähnlich, unabhängig davon, ob eine Knochenbeteiligung vorliegt oder nicht. Bei der Knochen-Beteiligung gibt es besondere Möglichkeiten, die aber nur im Ausnahmefall von Bedeutung sind. Beste Grüße. | 1) Die Therapie der Sarkoidose kann die Schmerzen im Becken und in der LWS verbessern.  2) Bei Knochen-Sarkoidose und LWS-Sarkoidose ist die Behandlung im Vordergrund des Errechnens der Risiken und Nutzen. Daher ist der Therapieplan individuell auf Ihren Verlauf, die Schwere der Krankheit, die Anzahl der Knochenbeteiligungen und die Lokalisation der Knochenbeteiligungen ausgerichtet. Daher könnten die Behandlungsmöglichkeiten sehr variabel sein.  3) Die medikamentösen Therapien umfassen die Kortikosteroide, TNF-Blocker oder Immunmodulatoren. In der Regel werden die Kortikosteroide als Therapiefirst-line-Therapie eingesetzt. Die Behandlung mit TNF-Blockern und Immunmodulatoren wird zusätzlich zur Steroidtherapie eingesetzt. Eine Medikation mit Zytostatikachemotherapie kann nötig sein, um die Krankheit zu kontrollieren.  4) Eine weitere Therapieplanung erfordert eine spezifische Untersuchung der Knochenbeteiligung. Derzeit gibt es keine Medikamente, die speziell für die Knochenbeteiligung entwickelt wurden. In der Praxis wird die Knochenbeteiligung mit den übrigen Bestandteilen der Sarkoidose behandelt. | Es tut mir leid zu hören, dass Sie durch diese schwierige und langwierige Diagnose gehen müssen. Sarkoidose kann in verschiedenen Organen auftreten und Ihre Beschreibung deutet auf eine systemische Beteiligung hin, insbesondere der Knochen. Zu Ihren Fragen: Eine medikamentöse Therapie der Sarkoidose, insbesondere mit Kortikosteroiden wie Methylprednisolon, kann oft zu einer deutlichen Verbesserung der Symptome und Schmerzen führen. Es ist möglich, dass Ihre Schmerzen dadurch reduziert werden. Granulome können sich unter medikamentöser Behandlung verkleinern oder inaktiv werden, was zu einer Stabilisierung oder sogar Verbesserung der Knochenstruktur führen kann. In einigen Fällen können jedoch strukturelle Veränderungen im Knochen zurückbleiben. Die Standardtherapie beginnt in der Regel mit Kortikosteroiden wie Methylprednisolon. Die Anfangsdosis und Dauer der Behandlung hängen von der Schwere der Erkrankung und dem Ansprechen auf die Therapie ab, beginnen aber oft mit einer hohen Dosis, die dann schrittweise reduziert wird. Zytostatika werden nur bei schwerwiegenden Fällen oder wenn Kortikosteroide nicht ausreichen, verwendet. Es ist verständlich, dass Sie sich Sorgen über die Nebenwirkungen machen. Diese sind zwar möglich, aber oft beherrschbar und sollten gegen die möglichen Vorteile der Behandlung abgewogen werden. Bitte besprechen Sie Ihre Bedenken ausführlich mit Ihrem Pneumologen, um eine individuell angepasste Therapie zu finden. Es ist wichtig, die Behandlung nicht zu verzögern, insbesondere bei Knochenbeteiligung, um weiteren Schaden zu vermeiden. |
| Bei Verdacht auf Sarkoidose (bihiläre Lymphenadopathie (Lymphknotengrösse 25 mm, 23 mm, 18 mm, 13 mm, Restriktive Ventilationsstörung unbekannter Genese), Reizhusten, tendenziell abnehmend, Sprechdyspnoe, Müdigkeit, Atemnot, Panikattacken, Druck auf Brust, linker Lungenflügel fühlt sich an, als würde er nicht mehr belüftet, Herz fühlt sich krank an, es wurde eine Reizleitungsstörung im III festgestellt, ohne Diagnose. Eine Bronchoskopie ergab fibrotisches Gewebe, aber keine Granulome. Bringt ein PET-CT des Herzens etwas, um die Sarkoidose nachzuweisen? | Die Diagnose einer Sarkoidose kann durch verschiedene Untersuchungen gestellt werden, wie z.B. eine histologische Untersuchung, eine Lungenspülung bei der Bronchoskopie (BAL), typische Bildgebung, Blut-Test-Ergebnisse und den Verlauf bzw. das klinische Bild. Eine einzelne diagnostische Maßnahme wie ein CT des Herzens reicht dafür nicht aus. Zur Feststellung eines Befalls des Herzens ist die Magnet-Resonanz-Tomografie (MRT) wahrscheinlich besser geeignet, allerdings nur unter bestimmten Bedingungen. Es ist nicht ungewöhnlich, dass bei der Bronchoskopie keine typischen Granulome gefunden wurden, da die Biopsien sehr klein sind. Wurde eine Broncho-alveoläre Lavage (BAL) durchgeführt? Beste Grüße. | Eine PET-CT des Thorax mit FDG-Markierung ist eine der bildgebenden Untersuchungen der Wahl bei der Sarkoidose, die zuverlässige Befunde liefert, die bei klinischer und/oder histologischer Verdachtsanamnese auf eine Sarkoidose helfen, eine definitive Diagnose der Sarkoidose zu stellen . In der PET-CT der Lunge von Patienten mit Sarkoidose wurde in früheren Studien eine bilaterale diffuse Hypermetabolie der Lungen in den Subsegmenten des Mediastinums und der Lungenseiten, die sich bilateral im Rahmen einer symmetrischen Verteilung mit einer prädilektiven Lokalisation an der Peripherie der Lungen auf dem Höhepunkt der Krankheit zeigte. Im Gegensatz dazu wurde bei Patienten mit Sarkoidose und einer frühen pulmonalen Beteiligung eine diffuse Hypermetabolie in der PET-CT der Lunge nicht regelmäßig festgestellt. | Ein PET-CT des Herzens kann hilfreich sein, um aktive Entzündungsherde bei Sarkoidose nachzuweisen, besonders wenn andere diagnostische Methoden keine eindeutigen Ergebnisse geliefert haben. Da die Bronchoskopie keine Granulome gezeigt hat, könnte ein PET-CT zusätzliche Informationen bieten, insbesondere bei der Beurteilung von Herzbeteiligungen. Es ist jedoch wichtig, diese Möglichkeit mit Ihrem behandelnden Arzt zu besprechen, um die beste Vorgehensweise für Ihre spezifische Situation zu bestimmen. |
| Vor 18 Jahren wurde bei meiner Frau Sarkoidose an der Lunge diagnostiziert. Inzwischen ist ihr gesundheitlicher Allgemeinzustand katastrophal (Atemnot, Krämpfe in allen Muskelregionen des Körpers, Depression, leichte Verwirrtheit, Gangunsicherheit ... ... ...)  Können das Folgeerscheinungen einer Sarkoidose sein? | Die Sarkoidose kann als Systemerkrankung praktisch alle Organe und also auch das Gehirn oder die Nerven betreffen. Allerdings muss man, um die Beschwerden auf die Sarkoidose zu beziehen, dann auch entsprechende Befunde nachweisen. Fehlen solche Hinweise auf eine Neuro-Sarkoidose, muss man andere Erkrankungen ausschließen. Ich wünsche alles Gute | Es könnte sein, dass Sarkoidose das Auftreten von Symptomen wie Atemnot, Krämpfen in allen Muskelregionen, Depression, Verwirrtheit und Gangunsicherheit begünstigt. Bei Sarkoidose kann es zu Lungenfibrose kommen, die zu Atemnot führen kann. Es ist möglich, dass die Sarkoidose eine Arthropathie verursacht hat, die zu Krämpfen in allen Muskelregionen führt. Depressionen können mit Sarkoidose assoziiert sein, und eine Sarkoidose kann auch zu einer generalisierten Erkrankung führen, die Gangunsicherheit verursacht. | Es ist möglich, dass diese Symptome Folgeerscheinungen einer fortgeschrittenen Sarkoidose sind, da die Erkrankung viele Organe betreffen kann und zu vielfältigen Beschwerden führen kann, einschließlich Atemnot und neuromuskulären Symptomen. Auch die psychischen und kognitiven Probleme können im Zusammenhang mit der chronischen Belastung und den systemischen Effekten der Erkrankung stehen. Es wäre jedoch wichtig, dass Ihre Frau von einem Spezialisten umfassend untersucht wird, um andere mögliche Ursachen auszuschließen und eine passende Behandlung zu finden. |
| Bei mir wurde vor 20 Jahren eine Lungensarkoidose diagnostiziert. Vor 7 Jahren wurde als Zufallsbefund zusätzlich eine Lebersarkoidose diagnostiziert. 2013 wurde daher eine immunsuppressive Therapie mit Kortison und Azathioprin gestartet, die ich leider wegen Unverträglichkeit nach 2 Monaten abbrechen musste. Verbesserung gab es ohnehin keine durch die Therapie.  Meine Beschwerden seit 2 Jahrzehnten: starke Fatigue sowie leichter Reizhusten.  Mein Kardiologe hat jetzt eine diastolische Funktionsstörung diagnostiziert, kennt sich aber leider nicht mit Sarkoidose aus. Kann diese Herzschwäche durch die Sarkoidose verursacht worden sein? Wenn ja, wie sollte sie therapiert werden (bei Unverträglichkeit von Kortison)? | Die Therapie der Sarkoidose ist praktisch immer auf einer Therapie mit Cortison aufbauend. Man kann versuchen, mit anderen Substanzen auf längere Sicht Cortison einzusparen. Cortison-Unverträglichkeit ist extrem ungewöhnlich, so dass möglicherweise damals Azathioprin der Grund für die Unverträglichkeit gewesen sein kann. Heute kann man die Azathioprin-Komplikationen durch einen genetischen Test vorab wahrscheinlich oder unwahrscheinlich machen. Aber das ist ein späteres Thema. Denn erst muss mit speziellen Untersuchungen des Herzens geklärt werden, ob eine Herz-Sarkoidose vorliegt. Wenn eine Behandlungsindikation besteht, sollte unbedingt in einem Sarkoidose-Zentrum noch einmal über einen Behandlungsversuch mit Cortison nachgedacht werden. Mit den besten Wünschen. | Die Lunge ist eine immunologische Kompartiment, und die Lunge, der Leber und die Galle haben einen hohen Anteil am Entwicklungsprozess der Sarkoidose. Es ist nicht ungewöhnlich, dass eine Sarkoidose die Lunge und Leber beeinflussen kann. Es gibt Hinweise darauf, dass Sarkoidose mit der kardiovaskulären Erkrankung assoziiert sein könnte. Es ist nicht klar, ob die Sarkoidose mit einer kardiovaskulären Erkrankung assoziiert ist oder ob der kardiovaskuläre Befund durch die Sarkoidose verursacht wird. In der Studie von 2014 wurde ein Zusammenhang zwischen der Sarkoidose und kardiovaskulären Erkrankungen gefunden. Die Autoren legen nahe, dass die Diastolische Funktionsstörung die Sarkoidose begünstigt. Zusätzlich wurde ein Zusammenhang zwischen Sarkoidose und dem Vorliegen von Kalzifikationen im Herzen gefunden, die auch mit der Diastolischen Funktionsstörung assoziiert sein könnte. Ein Zusammenhang zwischen der Sarkoidose und einer atherosklerotischen Erkrankung wurde jedoch nicht gefunden. | Ja, eine diastolische Funktionsstörung kann durch Sarkoidose verursacht werden, da die Erkrankung das Herz befallen kann und zu einer sogenannten kardialen Sarkoidose führen kann. Da Sie Kortison nicht vertragen, könnte eine Therapie mit anderen immunsuppressiven Medikamenten wie Methotrexat, Mycophenolatmofetil oder Infliximab in Betracht gezogen werden. Eine genaue Diagnose und Therapieplanung sollte durch ein spezialisiertes Sarkoidose-Zentrum oder einen erfahrenen Rheumatologen und Kardiologen erfolgen. Es ist wichtig, eine regelmäßige Überwachung und Anpassung der Therapie sicherzustellen, um die bestmögliche Kontrolle der Erkrankung zu erreichen und die Symptome zu lindern. |
| Sehr geehrte Damen und Herren,  die Symptome meiner nachgewiesenen Sarkiodose halten sich in Grenzen.  Meine Ärzte raten mir derzeit nicht zu einer Kortisonbehandlung, da die Nebenwirkungen nicht im Verhältnis zu der Symptomatik stehen.  Ich bin derzeit seit drei Monaten krank geschrieben.   Meine Frage:  Kann ich die Entzündungen mit Hanf (medikamentös) behandeln und gibt es darüber bereits Erfahrungen?  Zu meiner Person:  männlich  59 Jahre alt  übergewichtig  Bluthochdruck  keine Vorerkrankungen  Über eine Antwort würde ich mich freuen,  mit freundlichen Grüßen | Eine Sarkoidose muss nicht unbedingt behandelt werden, insbesondere dann nicht, wenn keine Beschwerden oder bestimmte Organbeteiligungen oder Komplikationen bestehen. Das kann man aber nur unter Berücksichtigung aller Informationen beurteilen. Wissenschaftliche Studien zur Behandlung sind mir nicht bekannt. Man muss bedenken, dass medizinische Cannabis-Präparate ja nur für ganz bestimmte Probleme zugelassen sind. Sarkoidose gehört nicht dazu. Mit den besten Wünschen. | Ich habe keine speziellen Informationen über Sarkoidose, aber ich kann Ihnen sagen, dass CBD legal ist und von einem Arzt verschrieben werden kann. Es wurde jedoch nicht von der FDA zugelassen. CBD hat nachweislich entzündungshemmende Eigenschaften, die möglicherweise bei Ihrer Sarkoidose helfen könnten. Die Auswirkungen auf den Menschen sind jedoch noch nicht gut untersucht worden. | es gibt einige Hinweise darauf, dass medizinisches Cannabis entzündungshemmende Eigenschaften hat und bei bestimmten entzündlichen Erkrankungen hilfreich sein könnte. Allerdings ist die Datenlage zur Wirksamkeit bei Sarkoidose begrenzt, und es gibt keine klaren Richtlinien oder umfangreiche Studien dazu. Es wäre ratsam, dies mit Ihrem behandelnden Arzt zu besprechen, da dieser Ihre gesamte Krankengeschichte und die möglichen Wechselwirkungen mit anderen Medikamenten, insbesondere angesichts Ihres Bluthochdrucks, besser einschätzen kann. Ihr Arzt kann Ihnen helfen, die beste Behandlungsoption für Ihre individuelle Situation zu finden.  Mit freundlichen Grüßen und den besten Wünschen für Ihre Gesundheit. |
| Ich bin 44 Jahre und leide seit ca. 6 Jahren unter einer Lungen-Sarkiodose. Um meine Lungenfunktion zu trainieren überlege ich derzeit eine Sauna zu erwerben.  Können Sie mir bitte sagen ob regelmäßige Saunabesuche empfehlenswert sind und auf was ich evtl. zu achten habe.  Vielen Dank. | Eine wissenschaftlich begründete Aussage zu Ihrer Frage ist nicht möglich. Ein Training oder eine Verbesserung der Lungen kann man mit regelmäßigen Saunagängen nicht erreichen. Sauna ist für viele Patienten mit leichten und mittelgradigen Veränderungen der Lungenfunktion gut verträglich, bei höhergradigen Veränderungen kann aber die Toleranz eingeschränkt sein. Mit den besten Grüßen. | Wenn Sie regelmäßige Saunabesuche machen, kann dies zu einer Verbesserung der Lungenfunktion führen und helfen, die Symptome abzubauen. Aufgrund der Erkrankung und der Medikamenteneinnahme sollten Sie jedoch unter der Beobachtung eines Gesundheitspersonals saunabesuche anstreben. Wenn Sie mit der Saunabehandlung beginnen, ist es wichtig, sich anfänglich langsam zu steigern und sich an den Regeln der Saune zu halten. Es sollte immer ein kühlender Gegenschmier vorhanden sein, um sich schnell abzukühlen. Zusätzlich sollten Sie nach einer Sauna-Session ein Wasser trinken, um die Dehydratation zu vermeiden und die Blutdruck-Regulation zu stabilisieren. | regelmäßige Saunabesuche können bei Lungensarkoidose förderlich sein, da sie die Durchblutung und die allgemeine Entspannung fördern. Es ist jedoch wichtig, dass Sie dies vorher mit Ihrem behandelnden Arzt besprechen, da die Hitze in der Sauna und die plötzlichen Temperaturwechsel für einige Menschen belastend sein können. Achten Sie darauf, ausreichend Flüssigkeit zu sich zu nehmen, um Dehydrierung zu vermeiden, und hören Sie auf Ihren Körper, um Überanstrengung zu verhindern. Mit freundlichen Grüßen. |
| Gibt es einen Zusammenhang zwischen Sarkoidose und Lipomen? | Einen solchen Zusammenhang kenne ich selbst nicht und konnte auch nichts dazu in der wissenschaftlichen Literatur finden. Das heißt, weder scheint es einen ursächlichen noch einen überdurchschnittlich häufigen zufälligen Zusammenhang zu geben. Mit den besten Wünschen. | Ich habe keinen Beweis für einen Zusammenhang zwischen Sarkoidose und Lipomen gefunden. Lipome werden in der wissenschaftlichen Literatur nicht mit Sarkoidose in Verbindung gebracht. Sarkoidose ist eine Autoimmunerkrankung, die durch die Bildung von nicht-verkäsenden Granulomen in verschiedenen Organen gekennzeichnet ist. Lipome sind gutartige Tumore, die aus reifem Fettgewebe bestehen und als lokalisierte Fettknötchen auftreten. Beide Erkrankungen können zwar unabhängig voneinander an bestimmten Stellen des Körpers auftreten, es wird jedoch nicht angenommen, dass sie miteinander zusammenhängen. Es ist wichtig, darauf hinzuweisen, dass weitere Forschungsarbeiten zu diesem Thema erforderlich sind, um diese Schlussfolgerung zu bestätigen. Wenn Sie sich Sorgen um Ihre Gesundheit machen, ist es immer am besten, einen Arzt zu konsultieren, um eine angemessene Diagnose und einen Behandlungsplan zu erhalten. | Es gibt keinen direkten Zusammenhang zwischen Sarkoidose und Lipomen. Sarkoidose ist eine entzündliche Erkrankung, die vor allem die Lungen und Lymphknoten betrifft, während Lipome gutartige Fettgewebswucherungen sind. Beide können gleichzeitig bei einer Person auftreten, aber sie haben unterschiedliche Ursachen und Pathologien. Wenn Sie weitere Fragen oder Symptome haben, sollten Sie dies mit Ihrem Arzt besprechen. |
| Kann durch Impfungen (Tollwut, Hepatitis A/B etc.) eine geheilte Sarkoidose (mit schwerem Verlauf im Alter von Anfang 20) nach ca. 30 Jahren wieder ausgelöst werden? Und wenn ja, wie lässt sich diese diagnostizieren und therapieren? Welche Symptome zeigen sich? Wie ist die Prognose? | Erfahrungsgemäß und auf Grund der Datenlage können Impfungen die Beschwerden der Sarkoidose vorübergehend verschlechtern, wenn die Sarkoidose noch aktiv ist, aber keine Sarkoidose auslösen oder nach Jahren reaktivieren. Beste Grüße | Ja, es ist möglich, dass eine latente (oder bereits ausgeheilte) Sarkoidose durch eine Impfung (wie Pocken, Hepatitis A/B usw.) ausgelöst wird. Dies kann aber erst nach einer gewissen Zeit geschehen. Außerdem ist es nicht immer möglich, zwischen einer Sarkoidose, die durch eine Impfung ausgelöst wurde, und einer „normalen“ Sarkoidose zu unterscheiden. Die Diagnose der Sarkoidose basiert auf einer Kombination aus Symptomen, Anzeichen und Laboruntersuchungen. Bildgebende Untersuchungen wie Röntgenaufnahmen des Brustkorbs, CT-Scans und MRT-Scans können helfen, eine Organbeteiligung festzustellen und Anzeichen einer Entzündung zu erkennen. Bluttests können helfen, andere Ursachen für Lungenknötchen auszuschließen. | Es gibt keine Hinweise darauf, dass Impfungen wie gegen Tollwut oder Hepatitis A/B eine geheilte Sarkoidose nach vielen Jahren erneut auslösen können. Sarkoidose kann durch verschiedene Faktoren beeinflusst werden, aber Impfstoffe gehören normalerweise nicht dazu. Sollte Sarkoidose dennoch erneut auftreten, wird sie typischerweise durch Symptome wie anhaltenden Husten, Kurzatmigkeit, Hautausschläge oder geschwollene Lymphknoten diagnostiziert und kann mittels Röntgen, CT-Scans und Biopsien bestätigt werden. Die Therapie besteht oft aus Kortikosteroiden oder anderen entzündungshemmenden Medikamenten. Die Prognose variiert, aber viele Patienten erleben eine gute Kontrolle der Symptome mit Behandlung. |
| Hallo, ich habe 2 Fragen:   1. ist man mit einer Sarkoidose generell anfälliger für Viren oder Bakterien? Fällt also eine Erkältung oder Grippe heftiger aus als bei Leuten ohne Sarkoidose? Schließlich handelt es sich ja um eine Autoimmunerkrankung. Ist also das Immunsystem dann grundsätzlich zu schwach (man wird schneller und heftiger krank) oder gar zu stark (bei Krankheit überschießt das System und somit fällt eine Krankheit wie z.B. Grippe heftiger aus)?   2. Kann Reflux Sarkoidose auslösen? Ich habe schon gehört, dass Reflux eine Lungenentzündung auslösen kann. Aber auch eine Sarkoidose der Lunge? | Es gibt eine neue Studie aus Schweden, die zeigt, dass unbehandelte Sarkoidosepatienten ein um ca. 50% erhöhtes Risiko für eine "schwere Infektion" haben. Bei Patienten, die immunsuppressiv behandelt werden, ist das Risiko noch höher. Es gibt jedoch keine soliden Daten für banale Erkältungserkrankungen und auch keine belastbaren Zahlen für Sars-CoV2. Der Verlauf einer Virusinfektion hängt mehr von der Ausgangssituation (Zustand der Lunge bzw. Intensität/Dosis der Immunsuppression) ab als von der Diagnose allein. Bei stark ausgeprägter Lungen-Sarkoidose tritt häufiger ein Reflux auf, der jedoch eher eine Folge der veränderten Mechanik von Zwerchfell, Lunge und Atmung ist als eine ursächliche Frage. Mit den besten Wünschen. | 1. Patienten mit Sarkoidose werden im Allgemeinen nicht schwerer von Virusinfektionen betroffen als gesunde Patienten. Die Sarkoidose ist im Gegenteil eine Autoimmunerkrankung und führt zu einer Erhöhung der Aktivität des Immunsystems. Dies kann dazu führen, dass sich eine Virusinfektion stärker entwickelt als bei gesunden Personen.  2. Reflux kann eine Sarkoidose der Lunge auslösen. Die Reflux kann die Sarkoidose verstärken, indem es zu einer Erhöhung der Aktivität des Immunsystems kommt. Reflux wird auch als auslösendes Faktor für einzelne Sarkoidosefälle berichtet. Patienten mit Reflux sollten daher eine medizinische Behandlung erhalten. | Bei Sarkoidose handelt es sich um eine entzündliche Erkrankung, die das Immunsystem beeinflusst, weshalb Betroffene sowohl anfälliger für Infektionen als auch für eine überschießende Immunantwort sein können. Daher können Erkältungen oder Grippen tatsächlich schwerer verlaufen.  Reflux selbst löst keine Sarkoidose aus, kann aber die Lungen reizen und Entzündungen verursachen, was die Symptome von bereits bestehender Sarkoidose verschlimmern kann. Es gibt keine direkte Verbindung zwischen Reflux und der Entstehung von Sarkoidose. |
| 30 jährige Patientin mit gelegentlichen Hämoptysen, nach bronchoskopischer Abklärung ED einer Sarkoidose in 2022. Zudem zu einer Sarkoidoe passende Hauteffloreszenzen.  Im Rahmen der augenärztlichen Abklärung wurde jetzt der V.a. Flammer Syndrom gestellt.   1.Gibt es Überschneidungen von Sarkoidose zum Flammer Syndrom?  2. Gibt es in NRW eine interdisziplinäre Ambulanz für Sarkoidose erkrankte? | Es gibt keinen bekannten Zusammenhang, wobei viele Beschwerden zu beiden Krankheitsbildern passen. Ja, es gibt eine ASV Ambulanz für Sarkoidose in NRW. Beste Grüße | Eine Überschneidung von Sarkoidose und Flammer-Syndrom wird beschrieben. Sarkoidose kann das Flammer-Syndrom verursachen und verstärken. 2. Eine interdisziplinäre Sarkoidose Ambulanz findet sich in NRW in den Städteln Duesseldorf und Bochum. | Sarkoidose und das Flammer-Syndrom haben keine direkte pathophysiologische Verbindung, obwohl beide vaskuläre und entzündliche Komponenten aufweisen können. In NRW gibt es spezialisierte interdisziplinäre Ambulanzen für Sarkoidose, beispielsweise an der Ruhr-Universität Bochum (Universitätsklinikum Knappschaftskrankenhaus Bochum) und der Universitätsklinik Düsseldorf, die umfassende Betreuung durch verschiedene Fachrichtungen anbieten. Es wäre sinnvoll, dort vorstellig zu werden, um eine koordinierte Behandlung zu gewährleisten. |
| Sehr geehrte Damen und Herren, ich habe seit 2007 die Diagnose Sarkoidose im Kehlkopfbereich. Über die Jahre hinweg gab es verschiedene aktive Phasen der Krankheit, seit 2012 ist die Krankheit nicht aktiv aber trotzdem allgegenwärtig. Ich muss aber keine Medikamente wie Kortison, oder andere nehmen. Ich gehe eigentlich jährlich zur Kontrolle zur Phoniatrie. Dieses Jahr aufgrund von Corona jedoch nicht. Nächstes Jahr möchte ich mich der Vorsorge widmen und auch einen Pneumologie aufsuchen, um zu schauen ob auch in anderen Organen die Krankheit nicht ausgebrochen ist.  Kommen wir nun zur eigentlichen Frage:  Ist es trotz Sarkoidose möglich risikofrei schwanger zu werden und ein gesundes Kind zu bekommen? Ich weiß die Frage ist weit hergeholt. Ich selber konnte aber nie nachvollziehen woher ich diese Erkrankung bekommen habe und natürlich ist die Sorge groß ich könnte im Falle einer Schwangerschaft diese Erkrankung an mein Kind weitergeben.  Ich hoffe sie können mir helfen und meine Frage beantworten.  Mit freundlichen Grüßen | Es ist möglich und risikofrei mit Sarkoidose schwanger zu werden, es sei denn eine kardiale oder neurologische Beteiligung (die sogenannte schwere Sarkoidose) liegt vor. Die Sarkoidose ist keine genetische/vererbbare Erkrankung. Eine familiäre Sarkoidose tritt selten auf. Im Allgemeinen haben Angehörige von Patienten mit Sarkoidose ein zwei- bis fünffach erhöhtes Risiko, an Sarkoidose zu erkranken. Es ist aber nur eine Risiko-Einschätzung. Wir hoffen Ihnen geholfen zu haben. Mit den besten Wünschen. | Natürlich ist es möglich, trotz Sarkoidose ein Gesundes Kind zu bekommen. | Es ist durchaus möglich, trotz Sarkoidose risikofrei schwanger zu werden und ein gesundes Kind zu bekommen. Ihre Krankheit ist seit Jahren inaktiv, und Sie benötigen keine Medikamente wie Kortison, was ein positives Zeichen ist. Während der Schwangerschaft sollten Sie engmaschig von Ihrem behandelnden Arzt überwacht werden, um sicherzustellen, dass die Sarkoidose nicht reaktiviert wird. Die Wahrscheinlichkeit, die Krankheit direkt an Ihr Kind weiterzugeben, ist gering, da die genaue Ursache der Sarkoidose unklar ist und genetische Faktoren nur eine teilweise Rolle spielen. Besprechen Sie Ihre Pläne und Sorgen mit Ihrem Pneumologen und Gynäkologen, um eine individuelle und umfassende Betreuung zu gewährleisten. Mit freundlichen Grüßen. |
| Diagnose: Sarkoidose ist gesichert  Diagnose: Neurosarkoidose zu ca 90% gesichert (Untersuchung Nervenwasser spricht dafür. Aktuelle Beschwerden: Wechselnde und wandernde Missempfindungen. FRAGE: Medikamente ja oder nein? (Lungenfachärztin sagt nicht erforderlich, Neurologe soll entscheiden. Neurologe sagt das gleiche nur negiert) Ich komme aus Solingen und finde keinen Neurologen, der sich mit diesem Krankheitsbild auskennt. | Es ist nicht ungewöhnlich, dass bei einer Sarkoidose, die nicht nur die Lunge betrifft, die Therapieentscheidung nicht von der Lungensituation abhängt. In solch einer Situation ist es wichtig, dass man in einem Zentrum betreut wird, in dem die verschiedenen Disziplinen zusammenarbeiten und nicht der eine auf den nächsten Kollegen verweist. Da ich die lokalen Gegebenheiten Ihres Wohnortes nicht kenne, ist es vielleicht am besten, wenn Sie sich an die Sarkoidose-Selbsthilfe wenden, die einen guten Überblick über die regionalen Spezialzentren hat und auch sonst sehr hilfreich sein kann. Beste Grüße. | Neurosarkoidose ist eine Rare Form der Sarkoidose. Die Symptome können schwankend und wechselnd sein, mit Missempfindungen in unterschiedlichen Regionen, häufig Begleiterkrankungen sind ein schwerwiegendes Kopfschmerz und ggf. Nervenlähmungen. Eine Diagnose ist erst mit einer Biopsie der ZNS-Regionen (ZNS: Zentralnervensystem) möglich. Die Diagnose wird durch eine Erhöhung der ZNS-Liquorzellenzahl und eine ZNS-Liquorzellveränderung gestellt. Es gibt verschiedene Medikamente, die die Symptome der Neurosarkoidose lindern können. Bei der Neurosarkoidose können sich die Symptome verlaufen. Sie müssen nicht immer wieder auftreten. | Es tut mir leid, dass Sie diese Verwirrung erleben. Bei Neurosarkoidose können Medikamente wie Kortikosteroide oder Immunmodulatoren hilfreich sein, besonders wenn neurologische Symptome bestehen. Ein spezialisierter Neurologe, idealerweise an einer Universitätsklinik oder einem spezialisierten Zentrum für seltene Erkrankungen, wäre empfehlenswert. Da Sie in Solingen wohnen, könnten Sie Kliniken in Köln oder Düsseldorf in Erwägung ziehen, die möglicherweise mehr Erfahrung mit Neurosarkoidose haben. Sprechen Sie erneut mit Ihrem Hausarzt oder Ihrer Lungenfachärztin, um eine Überweisung zu einem spezialisierten Zentrum zu erhalten. |
| Bei mir wurde eine Sarkoidose diagnostiziert, es handelt sich leider nicht nur um eine Sarkoidose der Lunge ,sondern sie betrifft sehr stark die Haut, das Herz und andere Organe. An wen kann ich mich wenden, um Hilfe zu erhalten ? | Es ist wichtig, sich an ein Sarkoidosezentrum zu wenden, in dem die verschiedenen Disziplinen zusammenarbeiten und nicht der eine auf den nächsten Kollegen verweist. Ganz wichtig ist natürlich die Frage, welche der wahrscheinlichen oder gesicherten Sarkoidose-Beteiligungen Beschwerden machen. Es ist vielleicht am besten, wenn Sie sich an die Sarkoidose-Selbsthilfe wenden, die einen guten Überblick über die regionalen Spezialzentren haben und auch sonst sehr hilfreich sein können. Beste Grüße. | Sarkoidose ist eine seltene und nicht-infektiöse Krankheit, welche die Organsysteme betrifft und mit einer starken Entzündung einhergeht. Die Lunge ist die häufigste betroffene Organe, gefolgt von den Knochen. Eine Sarkoidose kann auch mit schweren Krankheitsverläufen einhergehen, die nicht sicher vorherzusehen sind, und deshalb eine regelmäßige Verlaufskontrolle erforderlich ist. Es ist wichtig, eine Sarkoidose zu erkennen und die richtige Behandlung zu erhalten, um Komplikationen und eine schwere Erkrankung zu vermeiden. Daher ist die richtige Diagnostik, Behandlung und Überwachung der Krankheit besonders wichtig . | Es tut mir leid zu hören, dass Sie mit dieser Diagnose konfrontiert sind. Sie sollten sich an ein spezialisiertes Sarkoidose-Zentrum oder einen erfahrenen Rheumatologen wenden, der Erfahrung mit systemischer Sarkoidose hat. Zusätzlich könnten ein Kardiologe für Ihr Herz und ein Dermatologe für Ihre Haut sinnvoll sein. Ein interdisziplinärer Ansatz ist oft am besten, um alle betroffenen Organe optimal zu behandeln. Ihr Hausarzt kann Ihnen helfen, die richtigen Fachärzte zu finden und die Koordination Ihrer Behandlung zu unterstützen. |
| 2003 Sarkoidose II. Grad, akut, Erythema nodoum, bililäre Lymphadenopathie, fleckig diffuse Verschattungen RB 2, stark geschwollene schmerzende Gelenke, komplett ausgeheilt. Bis 2008 jährliche Kontrolle mit Röntgen = immer ohne Befund = seit rund 20 Jahren keine Sarkoidose mehr.  Seit 2015 chronische Bronchitis, mein Pneumologe hat meine Lunge in 2015, 2017 und 2022 geröntgt. Insgesamt wurde meine Lunge bis jetzt 12 Mal geröntgt.   FRAGE: Ist es sinnvoll, meine Lunge so häufig zu röntgen, da ja auch jede Strahlung schädlich sein kann? Oder ist dies sinnvoll, um eine evtl. COPD frühzeitig zu erkennen? Vielen Dank vorab | Grundsätzlich ist nach durchgemachter "akuter Sarkoidose" eine Nachsorge sinnvoll. Ob im Einzelfall mehrere Rö-Aufnahmen erforderlich sind, kann man nur in der individuellen Situation entscheiden. Die Suche nach einer COPD (Rauchervorgeschichte?) erfolgt nicht typischerweise mit Röntgen-Kontrollen. In jedem Fall kann mit der Erhebung der Anamnese (in diesem Fall Zwischengeschichte), der körperlichen Untersuchung und der Lungenfunktionsdiagnostik die Notwendigkeit für Rö-Untersuchungen auf ein Maß reduziert werden, dass eine Gefährdung durch Strahlung minimiert. Mit den besten Grüßen. | Im Falle einer chronischen Lungenerkrankung wird die Diagnostik in Abhängigkeit von der Art und dem Schweregrad der Erkrankung und den individuellen Umständen angepasst. Die Röntgenuntersuchung wird in der Regel als Initialdiagnostik und zur Beurteilung des Fortschreitens einer Erkrankung angewendet. Sie ist eine nützliche und wertvolle Methode, um Lungenveränderungen zu erkennen. Die meisten Erkrankungen, die Lungen befallen, lassen sich gut auf Röntgenbildern erkennen. Diese Veränderungen umfassen meist das Auftreten von Verschattungen, Ödemen oder Noduli, die auch mit dem Vorliegen einer COPD assoziiert sind. | Es ist verständlich, dass Sie sich Sorgen um die Strahlenbelastung machen. Während häufiges Röntgen potenziell schädlich sein kann, kann es in Ihrem Fall gerechtfertigt sein, um mögliche chronische Lungenprobleme wie COPD frühzeitig zu erkennen und zu überwachen. Es wäre jedoch ratsam, diese Bedenken mit Ihrem Pneumologen zu besprechen, um möglicherweise alternative Überwachungsmethoden wie regelmäßige Lungenfunktionstests oder andere bildgebende Verfahren zu erwägen, die weniger Strahlenbelastung mit sich bringen. |
| Ich habe seit längeren Probleme mit den Gelenken an den Fingern und überwiegend das linke Bein und der linke Arm, zudem bekomme ich regelmäßig mit meiner Haut richtige Juckanfälle wobei sich dann kurzeitig kleine Knötchen/Blasen/Pickelchen bilden und sich diese entzünden, weil ich vor jucken nicht aufhören kann. An meiner Kopfnarbe bildet sich eine Beule, die letztes Jahr schon einmal da war, dann aber wieder verschwunden ist und nun wieder da und auch leichte Schmerzen verursacht. Zudem habe ich auch sehr starke Stimmungsschwankungen (ich weiß nicht ob das unter Depressionen fällt). Zu meiner chr. Lungensarkoidose wurde 2012 auch eine Thrombose, Lungenembolie und Faktor 5 Leiden festgestellt. Ich nehme Schilddrüsen Tabletten (Unterfunktion) und Blutverdünner. Für die Sarkoidose wurde bisher noch nichts verordnet. Aber irgendwie kommen immer mehr Faktoren dazu, die mein Leben und mein Lebensgefühl verschlechtern. Ich befinde mich 1 x im Jahr beim Lungenarzt in Behandlung. Gibt es Ärzte oder Stationen an dem man mich noch einmal richtig durchchecken kann oder wer kann mir diesbezüglich weiter helfen?  Vielen Dank im Voraus  Mit freundlichen Grüßen | Hallo, auch wenn es verständlich ist, dass man bei einer Sarkoidose alle Beschwerden damit in Zusammenhang bringt, muss das ja längst nicht immer so sein. Es macht sicher Sinn, dass Sie sich einmal bei einem Spezialisten vorstellen. Über die Sarkoidose Selbsthilfegruppen finden Sie am leichtesten einen geeigneten Spezialisten - vielleicht sogar in Ihrer Nähe. Gehen Sie mal auf die Webseite der Bundesvereinigung, dort finden Sie alle erforderlichen Informationen. Beste Grüße. | Ich finde die meisten Ihrer Beschwerden für nicht näher zu identifizieren, daher möchte ich Sie weiter an das Krankenhaus schicken. Sie könnten einen Hautspezialisten aufsuchen, der Ihr Jucken besser untersuchen kann. Ihr Bein- und Armproblem könnte mit dem Sarkoidoseproblem zusammenhängen und könnte mit einer Biopsie geklärt werden. Der Lungenarzt könnte ein Röntgen machen, um das Sarkoidoseproblem zu untersuchen. Ihr Depressionsgefühl könnte auch mit dem Sarkoidoseproblem zusammenhängen. Sie könnten sich nochmal beim Psychiater wenden. | Es klingt, als hätten Sie eine komplexe medizinische Geschichte mit mehreren gleichzeitig bestehenden Gesundheitsproblemen. Ihre Symptome, einschließlich Gelenkschmerzen, Hautprobleme, Kopfschwellung und Stimmungsschwankungen, könnten miteinander verbunden sein und erfordern eine umfassende Untersuchung. Ich empfehle Ihnen, sich an eine Universitätsklinik oder ein spezialisiertes Zentrum für seltene Erkrankungen zu wenden, wo interdisziplinäre Teams aus verschiedenen Fachrichtungen (Rheumatologie, Dermatologie, Neurologie und Psychiatrie) zusammenarbeiten können. Diese Zentren sind darauf spezialisiert, komplexe Fälle wie Ihren zu diagnostizieren und zu behandeln. Besprechen Sie dies auch mit Ihrem Lungenarzt, der Ihnen möglicherweise eine Überweisung ausstellen kann. |
| Sehr geehrte Damen und Herren,  bei mir wurde Anfang Februar eine chronische Sarkoidose der Lunge mit Milz und Leberbefall festgestellt. Mit dieser Diagnose wurde ich aus dem Krankenhaus entlassen. Für den 13.05 habe mit Mühe und Not einen Termin bei einer Pneumologin hier vor bekommen. Nun bin ich seit 4 Wochen von meinem Hausarzt krank geschrieben aufgrund der aktuellen Corona Situation. Auf Nachfrage bei der Pneumologin ob ich eventuell auch früher kommen kann, da meine Atemprobleme zugenommen haben, wurde mir die Auskunft gegeben, dass dieser Termin aufgrund von Corona eventuell gar nicht statt findet. Mein Hausarzt meinte er könnte das nicht nachvollziehen ich soll versuchen bei einem anderen Pneumologen einen Termin zu bekommen, das ist allerdings aussichtslos bis November. Ich habe im Sarkoidose Zentrum in Würzburg angerufen auch hier wurde ich abgewiesen. Ich fühle mich etwas hilflos und auch allein gelassen mit meiner Erkrankung.  Was können Sie mir raten?  Ich danke Ihnen für Ihre Bemühungen.  Mit freundlichen Grüße | Hallo, aus Ihrer Darstellung kann ich nicht so recht entnehmen, ob Sie akute Beschwerden haben oder ob es mehr die vielen Fragen sind, die unbeantwortet sind, die Sie so eilig zum Pneumologen treiben. Beides könnte ich gut verstehen, bei akuten Beschwerden muss natürlich ein schnellerer Termin möglich sein, bei Fragen kann es auch mal eine Telefon- oder Videosprechstunde sein. Viele Fragen werden in den Foren der Selbsthilfegruppen besprochen, so dass Sie vielleicht dort auch Adressen zu Regionalgruppen usw. finden. Mit den besten Grüßen. | Es tut mir leid, das zu hören. Es kann schwer sein, mit chronischer Sarkoidose umzugehen.  Ich empfehle Ihnen, erneut einen Spezialisten aufzusuchen, um eine zweite Meinung einzuholen. Überprüfen Sie außerdem Ihre Sauerstoffsättigung und gehen Sie in die Notaufnahme, wenn Sie sich sehr kurzatmig fühlen. | Es tut mir leid zu hören, dass Sie sich in dieser schwierigen Situation befinden. Ich empfehle Ihnen, weiterhin regelmäßig Kontakt mit Ihrem Hausarzt zu halten, da er Ihre Situation am besten kennt und eventuell eine Überweisung an einen anderen Spezialisten beschleunigen kann. Es könnte auch hilfreich sein, Notfallambulanzen oder spezielle Zentren für Atemwegserkrankungen aufzusuchen, wenn Ihre Symptome sich verschlechtern. Manchmal können Hausärzte oder spezialisierte Kliniken auch über Telemedizin eine Beratung anbieten, was in der aktuellen Lage nützlich sein könnte. Bleiben Sie hartnäckig und suchen Sie weiter nach Möglichkeiten, medizinische Hilfe zu bekommen. |
| Sollte ich mich nach der Diagnose der Sarkoidose an eines der speziellen Zentren z.B. in Essen wenden oder weiterhin über den Hausarzt versorgt werden? | Hallo, bei der Zufallsdiagnose im Jugendlichen Alter ist ja oft nicht wirklich etwas zu tun. In Ihrem Alter sollte man aber zumindest einmal ein Referenzzentrum um eine Stellungnahme bitten, damit man nicht wichtige Dinge zu spät in die Wege leitet. Beste Grüße. | Es empfiehlt sich bei der Sarkoidose nach einer Diagnose immer einer spezialisierten Einrichtung zuzugehen. In Deutschland gibt es derzeit 3 Zentren, die in Essen, Mainz und München angesiedelt sind. Eine Aufnahme über einen Hausarzt wird nicht empfohlen. | Nach der Diagnose von Sarkoidose ist es sinnvoll, sich an ein spezialisiertes Zentrum wie das in Essen zu wenden, um eine optimale Betreuung und Behandlung zu gewährleisten. Diese Zentren haben umfassende Erfahrung und spezialisierte Kenntnisse, die bei komplexen oder fortgeschrittenen Fällen besonders wichtig sind. Ihr Hausarzt kann weiterhin eine wichtige Rolle in der allgemeinen Betreuung und in der Koordination Ihrer Pflege spielen. |
| Hallo!  1 ) Hilft eine Therapie im Radon-Heilstollen in Bad Gastein ( Österreich ) bei Sarkoidose der Lunge?  2 ) Ist verstärktes Ausdauertraining bei Sarkoidose der Lunge wichtig und sinnvoll?  Vielen Dank | Hallo, belastbare Belege im wissenschaftlichen Sinn für einen therapeutischen Nutzen einer sog. Heilstollenbehandlung gibt es nicht. Ausdauertraining macht fast immer Sinn, auf die richtige Dosis kommt es an. Man kann solches Training übertreiben, dann ist es wegen Sauerstoffmangel oder Erschöpfung der Atempumpe oder Überlastung der rechten Herzkammer eher schädlich als nützlich. Wenn man sich beim sog. Training unterfordert, ist es nicht nützlich. Sie sollten sich bei Ihrem Sarkoidosesepzialisten beraten lassen, wo denn die Intensität des Trainings liegt, damit Sie nicht zu viel aber auch nicht zu wenig Belastung einplanen. Beste Grüße | Ja, es hat sich gezeigt, dass eine Therapie im Radon-Heilstollen in Bad Gastein bei Sarkoidose der Lunge wirksam sein kann. Verstärktes Ausdauertraining ist für Patienten mit Sarkoidose der Lunge wichtig und sinnvoll, wenn dieses Training nicht zu einem schweren Krankheitszustand führt. | Eine Therapie im Radon-Heilstollen in Bad Gastein kann bei einigen Patienten mit Sarkoidose der Lunge hilfreich sein, da sie entzündungshemmende Eigenschaften hat. Es gibt jedoch begrenzte wissenschaftliche Beweise, und es ist wichtig, dies mit Ihrem behandelnden Arzt zu besprechen. Verstärktes Ausdauertraining kann vorteilhaft sein, um die allgemeine Lungenfunktion und Fitness zu verbessern, sollte jedoch vorsichtig und unter medizinischer Aufsicht durchgeführt werden, da Überanstrengung vermieden werden sollte. Sprechen Sie mit Ihrem Arzt über ein geeignetes Trainingsprogramm. |
| Hallo,  Im November 2016 hatte ich erste Symptome (entzündete alte Tattoos, Erschöpfung, Uveitis, Atembeschwerden in Anstrengung). Diagnose Sarkoidose der Haut, der Augen und leichte Lungenbeteiligung im März 17, Medikation Cortison und später mit Azathioprin. Unter Medikation Besserung und schließlich Beschwerdefreiheit ab Juni 18. Erhaltungsdosis war dann für fast 2 Jahre 150 mg Imurek. Seit Mai 20 nehme ich keine Immunsuppressiva mehr.  Meine Frage: wie hoch ist die Chance, dass eine neue Tätowierung wieder einen Schub auslöst? | Hallo, da Ihre Haut schon einmal mit einer überschießenden Reaktion auf die Tattoos geantwortet zu haben scheint, kann man nicht wirklich grünes Licht für einen neue Tätowierung geben. Ich kenne keine Studien zu dem Thema, aber ich würde Ihnen eher abraten. Mit den besten Wünschen. | Es ist schwierig zu beurteilen, wie hoch die Wahrscheinlichkeit ist, dass eine neue Tätowierung einen Schub auslösen könnte. Bei Vorliegen einer Sarkoidose mit einem vergangenen Schub besteht ein erhöhter Risikofaktor für einen Schub, und die Exposition mit einer neuen Tätowierung könnte dieses Risiko erhöhen. Die Tätowierung sollte daher nur mit großer Vorsicht erfolgen. Zudem ist es wichtig, sich bei der Entscheidung um eine Tätowierung zu beraten und unter ärztlicher Begutachtung zu erfolgen, da ein Schub auch potenziell schwerwiegende Folgen mit sich bringen kann. | Es ist schwierig, genau zu sagen, wie hoch das Risiko eines neuen Sarkoidose-Schubs durch eine neue Tätowierung ist, da dies individuell unterschiedlich sein kann. Da Ihre Sarkoidose seit 2020 ohne Immunsuppressiva stabil ist, besteht dennoch ein gewisses Risiko, da Tätowierungen eine entzündliche Reaktion hervorrufen können. Besprechen Sie dieses Vorhaben unbedingt mit Ihrem behandelnden Arzt, der Ihre Krankengeschichte kennt und eine fundierte Empfehlung geben kann. Es könnte auch hilfreich sein, mögliche Alternativen oder Vorsichtsmaßnahmen zu besprechen, um das Risiko zu minimieren. |
| Gemäss verschiedenen Studien ist offenbar das Risiko für Osteoporose erhöht bei Patienten mit Bronchiektasen, besonders, wenn eine chronische Infektion vorliegt und evtl. noch mehr, wenn bei Belastung der Sauerstoff absinkt. Welche Empfehlungen ergeben sich daraus für ältere PCD-Patienten bezüglich Osteoporose-Prophylaxe und bezüglich Messung der Knochendichte? | Hallo, das ist eine gute Frage. Bisher gibt es unseres Wissens keine Studien zu dieser Frage, man müsste zur Klärung eine Studie durchführen. Mit den besten Grüßen | Nach aktueller Evidenzleistung sollten Ärzte älterer PCD-Patienten bei Verdacht auf eine Osteoporose eine Messung der Knochendichte durchführen, um die Entwicklung einer Osteoporose rechtzeitig erkennen und entsprechend behandeln zu können. Dies gilt insbesondere für Patienten, die unter einer chronischen Infektion leiden. | Bei älteren Patienten mit Primärer Ciliärer Dyskinesie (PCD) und Bronchiektasen, insbesondere bei chronischen Infektionen und Hypoxie unter Belastung, ist eine sorgfältige Osteoporose-Prophylaxe wichtig. Empfohlen wird eine regelmäßige Knochendichtemessung (DXA-Scan) zur frühzeitigen Erkennung von Osteoporose. Zusätzlich sollten Patienten auf eine ausreichende Zufuhr von Calcium und Vitamin D achten und gegebenenfalls Supplemente einnehmen. Regelmäßige körperliche Aktivität und gegebenenfalls eine medikamentöse Behandlung nach ärztlicher Absprache können ebenfalls zur Vorbeugung beitragen. |
| Ich bin auf der Suche nach spezialisierten Ärzten ( und auch Kliniken ) für Sarkoidose. Die sich aber wirklich gut mit der Krankheit auskennen . Ich war schon in einigen Kliniken, die sich als Sarkoidose Klinik benannt haben und im Internet auch damit werben aber dies war dann eher enttäuschend.  Deshalb bin ich auf der Suche nach Spezialisierten Ärzten für Sarkoidose | Hallo, die Deutsche Sarkoidose Vereinigung e.V. pflegt eine Liste der Spezialambulanzen und -einrichtungen. Beste Grüße | Sarkoidose ist eine seltene Entzündungskrankheit des Lymphsystems und des Wachstums- und Verdauungssystems. In Deutschland wird sie am Institut für Sarkoidose in Berlin, am Krankenhaus der Barmherzigen Brüder St. Josef in Bremen und an der Sarkoidose Klinik Ruhr-Universität Bochum behandelt. Die Kliniken haben in ihren Fokus neben der Behandlung der Sarkoidose auch die Forschung von Sarkoidose. Zusätzliche Informationen sind auf der Homepage der Kliniken und den Stiftungs- bzw. Gesellschaftsseiten der genannten Einrichtungen zu finden. | Es tut mir leid zu hören, dass Sie bisher enttäuschende Erfahrungen gemacht haben. Für spezialisierte Sarkoidose-Behandlung empfehle ich renommierte Universitätskliniken oder spezialisierte Lungenkliniken, wie z.B. die Universitätsklinik Freiburg oder die Thoraxklinik Heidelberg. Diese Einrichtungen sind bekannt für ihre Expertise in der Behandlung von seltenen Lungenerkrankungen. Es könnte auch hilfreich sein, sich an Selbsthilfegruppen für Sarkoidose zu wenden, da diese oft Empfehlungen für spezialisierte Ärzte und Kliniken haben. Ich wünsche Ihnen viel Erfolg und gute Besserung. |
| Wo erhalte ich eine umfassende pulmologische Versorgung für mein Kind? | Hallo, die Zentren für Kinderchirurgie, wo solche Eingriffe vorgenommen werden, haben in der Regel erfahrene Partner an ihrer Seite, so dass ich grundsätzlich sagen würde, dass die Frage dort anzubringen wäre. Die Tatsache, dass Sie sich an uns wenden, könnte dafür sprechen, dass man Ihnen keinen Tipp geben konnte. In dem Fall sind Sie mit Ihrem Kind sicher in einem Zentrum mit Kinder-Pneumologie gut aufgehoben. Mit den besten Grüßen. | Ihr Kind kann bei einer umfassenden pulmologischen Versorgung gut versorgt werden. Es empfiehlt sich, sich mit einem Pneumologen in Verbindung zu bringen, um sich über das weitere Vorgehen informiert zu werden. Die Pneumologie ist eine Fachdisziplin, die sich mit Lungen- und Atemwegserkrankungen befasst. Es gibt verschiedene Versorgungsebenen, an denen ein Kind versorgt werden kann. Die erste Versorgungsebene ist die ambulante Versorgung. Hier werden Kinder von HausärztInnen versorgt und ein Pneumologe wird hinzugezogen, wenn Bedarf besteht. Die zweite Versorgungsebene ist die stationäre Versorgung. Hier werden Kinder in Krankenhäusern versorgt und Pneumologen werden hinzugezogen, wenn Bedarf besteht. Die dritte Versorgungsebene ist die spezialisierte Versorgung. Hier werden Kinder in spezialisierten Zentren versorgt und Pneumologen sind ständig vor Ort. Es wird empfohlen, sich mit einem Pneumologen in Verbindung zu bringen, um sich über das weitere Vorgehen informiert zu werden. Die Pneumologie ist eine Fachdisziplin, die sich mit Lungen- und Atemwegserkrankungen befasst. | Um eine umfassende pulmologische Versorgung für Ihr Kind zu erhalten, sollten Sie sich an ein spezialisiertes Kinderkrankenhaus oder eine Klinik mit einer Abteilung für Kinderpneumologie wenden. Ihr Kinderarzt kann Ihnen eine Überweisung ausstellen und Empfehlungen für spezialisierte Einrichtungen in Ihrer Nähe geben. Es ist wichtig, dass die Einrichtung Erfahrung in der Behandlung von Atemwegserkrankungen bei Kindern hat, um eine optimale Versorgung sicherzustellen. |
| Seid etwa 4 Wochen habe ich nun die Diagnose: Sarkoidose. Da ich noch nicht so viel über das Krankheitsbild weiß, aber sehr viel mit Menschen zusammenarbeite, würde ich gern wissen, ob ich zur Coronarisikogruppe gehöre und mich noch besser schützen muss.  MfG | Hallo, diese Frage kann man ohne Informationen zum aktuellen Gesundheitszustand nicht beantworten. Dazu müsste man nicht nur die Lungenfunktion kennen, man müsste auch wissen, ob Sie sich krank fühlen, ob irgendwelche Organbeteligung vorliegt usw. Mt andere Worten, eine solche Frage kann man nur beantworten, wenn man den Patienten vor sich sieht und die gesamte Krankengeschichte kennt. Fragen Sie Ihren Lungenfacharzt oder Hausarzt. Grundsätzlich kann man sagen, wenn die Sarkoidose keinen Veränderung der Lungenfunktion gemacht hat und keine höher dosierte Cortison-Therapie erfolgt, ist das Risiko, einen schweren Verlauf von COVID-19 zu bekommen, wahrscheinlich nicht erhöht. Beste Grüße. | Die Sarkoidose ist eine chronisch entzündliche Erkrankung mit unspezifischen Manifestationen in verschiedenen Organsystemen. Sarkoidosen können sich in der Lunge, am Herzen, am Hoden oder dem Knochenmark manifestieren und können mit einer Vielzahl von Krankheitsbildern verwechselt werden. Der Krankheitsverlauf kann über Monate oder Jahre verlaufen und wird in verschiedenen Phasen eingeteilt. Es ist wichtig, die richtigen Antibiotika zu verordnen, um die schwerwiegenden Folgekomplikationen dieser Erkrankung zu vermeiden. | Sarkoidose kann das Immunsystem schwächen und die Lunge betreffen, was potenziell das Risiko für schwere COVID-19-Verläufe erhöhen könnte. Es wäre ratsam, dass Sie zusätzliche Vorsichtsmaßnahmen ergreifen, wie das Tragen von Masken, häufiges Händewaschen und das Vermeiden von Menschenmengen. Konsultieren Sie Ihren behandelnden Arzt, um spezifische Empfehlungen für Ihre Situation zu erhalten. Mit freundlichen Grüßen. |
| Gibt es neue Therapiemöglichkeiten der Larynxsarkoidose außer Glucocorticoide im Schub(Patietin ist 24 Jahre alt)? Zur Zeit in stabiler Remission, aber bereits Z.n.3 akuten Schüben mit ausgeprägtem Larynxödem jeweils.  Ist eine mRNA COVID 19 Impfung bedenkenlos? | Hallo, es gibt neben Corticosteroiden auch andere Medikamente mit immunsuppressiver Wirkung wie Azathioprin und Methotrexat, die zur Behandlung der Sarkoidose eingesetzt werden können. Ob diese Medikamente jedoch ein besseres Nebenwirkungsprofil haben als Corticosteroide, muss im Einzelfall geprüft werden. Dies gilt insbesondere, wenn man über eine "off-label"-Therapie mit nicht für die Sarkoidose zugelassenen Medikamenten nachdenkt. Was die mRNA-Impfung betrifft, gibt es keine Einschränkungen. Beste Grüße. | Die aktuellen Daten zeigen eine reaktive Erhöhung der IL-6-Serumkonzentration nach COVID-19-Impfung . Diese Erhöhung wird im Rahmen einer schweren SARS-CoV-2-Infektion auch bei Patient:innen mit einer chronisch inflammatorischen Krankheit, wie beispielsweise einer chronisch entzündlichen Darmerkrankung, beobachtet . Die Daten über die mRNA-basierten COVID-19-Impfstoffe für Personen mit einer chronisch entzündlichen Krankheit sind bisher limitiert. Jedoch zeigen bislang verfügbare Daten aus Phase-II-Studien keine relevanten Arzneimittelwirkung für eine schwere COVID-19-Erkrankung in der Bevölkerungsgruppe mit chronisch entzündlicher Darmerkrankung . | Bei der Behandlung der Larynxsarkoidose werden neben Glucocorticoiden in akuten Schüben auch andere immunsuppressive Medikamente wie Methotrexat oder Azathioprin verwendet, um die Krankheitsaktivität zu kontrollieren. In stabiler Remission kann eine engmaschige Überwachung ohne dauerhafte Medikation ausreichen. Die mRNA-COVID-19-Impfung gilt im Allgemeinen als sicher, auch für Patienten mit Larynxsarkoidose, aber es ist wichtig, dies individuell mit Ihrem behandelnden Arzt zu besprechen, um mögliche Risiken und Nutzen abzuwägen. |
| Guten Tag,  2010 wurde bei mir Sarkiodose der Lunge diagnostiziert und erfolgreich behandelt. Seit einher Zeit habe ich Schmerzen an beiden großen Zehen und starke Schmerzen am linken Mittelhandknochen. Orthopädisch ist nichts festzustellen.  Kann dies, laut Sarkoidose-Verein eine Spätfolge der Sarkoidose sein. Wer kann dies untersuchen? Ich wohne in Süddeutschland.  Mit freundlichen Grüßen | Hallo, es ist grundsätzlich so, dass eine Sarkoidose sich auch am Knochen zeigen kann - typischerweise geht das aber mit ganz spezifischen radiologischen Veränderungen einher. Auch wenn das Befallmuster bei Sarkoidose nicht vorhersagbar ist, ist nicht automatisch von einem Sarkoidosebefund auszugehen. Ein Internist oder Rheumatologe kann das bestimmt näher eingrenzen, wobei die Sarkoidose nur zu den auch möglichen Ursachen zählen wird. Beste Grüße | Guten Tag, Sarkiodose der Lunge wird mit der Zeit in der Regel spontan abheilen. Es gibt eine Gruppe von Patienten mit Sarkoidose, welche lang anhaltende Schmerzen in den kleinen Gelenkflächen zeigen und sich nach längerer Zeit wieder verbessern. Ob es sich hierbei um eine Spätfolge der Sarkoidose handelt oder eine unabhängige Erkrankung ist nicht bekannt. Es gibt keine spezifischen Tests, um dies zu untersuchen. Ihr Hausarzt oder ein Facharzt könnte Ihr eine sorgfältige körperliche Untersuchung anbieten, um einen genaueren Zustand Ihrer Gelenkflächen zu erkennen. | es ist möglich, dass Ihre Beschwerden eine Spätfolge der Sarkoidose sind, da diese Erkrankung verschiedene Organe und Gewebe betreffen kann. Um dies abzuklären, sollten Sie einen Rheumatologen oder einen spezialisierten Sarkoidose-Experten aufsuchen. In Süddeutschland gibt es mehrere spezialisierte Kliniken und Zentren für seltene Erkrankungen, die Ihnen weiterhelfen können. Ihr Hausarzt kann Sie an die entsprechenden Fachärzte überweisen. Mit freundlichen Grüßen. |
| Sehr geehrte Damen und Herren,  ich suche in pucto Sarkoidose erfahrene Augenärzte in NRW, gerne Raum Bielefeld oder ganz NRW. Symptomatik voraussichtlich bislang am Auge. Bisher 2 Feldiagnosen, letze Diagnose Randfurchenkeratitis in einer Uniklinik. Zuletzt Verdacht auf allergische Bindehautentzündung, aber Allergietest negativ. Sarkoidose liegt jedoch bei der Mutter vor.  HNO-Arzt bittet um Abklärung.  Nach so vielen versch. Diagnosen werden Sarkoidose-erfahrene Augenärzte gesucht. Besten Dank vorab für weiterführende Hinweise/ Empfehlungen.  MfG | Hallo, es ist sehr ungewöhnlich, eine Augensarkoidose zu diagnostizieren, wenn sonst keine Sarkoidose vermutet werden muss. Die Sarkoidose der Mutter ist auch nur ein schwacher Hinweis. Sie können sich aber sicher an einen erfahrenen Augenarzt wenden, der Ihnen weiterhelfen kann. Beste Grüße | Im Sarkoidose-Forschungsnetz gibt es derzeit nur 2 Augenärzte, die sich an der Forschung über Sarkoidose beteiligt haben. Sie befinden sich in Hamburg, Deutschland, und Düsseldorf, Deutschland. Hier ist der Link zum Netzwerk: https://www.sarcoidose-forschungsnetzwerk.de/teampages/aug/aug_en.php#aug. | für die Abklärung Ihrer Symptomatik im Zusammenhang mit einer möglichen okulären Sarkoidose empfehle ich Ihnen, sich an Universitätskliniken oder größere Augenkliniken zu wenden, da diese häufig spezialisierte Fachärzte haben. In NRW können Sie z.B. die Augenklinik der Universität Düsseldorf oder die Universitäts-Augenklinik in Münster kontaktieren. Beide Kliniken haben Erfahrung mit komplexen und seltenen Erkrankungen wie der Sarkoidose. Ich wünsche Ihnen alles Gute bei der weiteren Diagnostik und Behandlung. Mit freundlichen Grüßen |
| Guten Tag,  mein mein Mann hatte im Mai 20 eine Fettgewebeentzündung im Bein, Begleiterscheinungen waren starke Rückenschmerzen und Fieber und hoher CRP Wert bis fast 300. Nach Behandlung mit dem Reserveantibiotikum Menopenem wurde er nach fast 3 Wochen Krankenhaus Anfang Juni gesund entlassen.  Bis Anfang September war mein Mann völlig gesund und beschwerdefrei.  Seit Mitte September ist mein Mann nun erneut im Krankenhaus. Diesmal sind die Beschwerden starke Bauchschmerzen (tendenziell links) und besonders wieder der Rücken, die Flanken auch hier besonders links.  Nach verschiedener Bildgebung hat man einen stark vergrößerten Lymphknoten im Bauchraum festgestellt. Eine Probeentnahme von diesem war nicht erfolgreich. Magen- und Darmspiegelung unauffällig. Ultraschall andere Organe ebenfalls unauffällig. Wieder Versuch der Behandlung mit Antibiotika zeigte keinen Erfolg.  Nach zwei Wochen wurde mein Mann ins UK Eppendorf verlegt.  Im CT der Lunge zeigen sich typische Granulome für Sakiodose. Er hat trockenen Husten, der zunehmend schlimmer wird. Es wurde eine Lungenspülung und Proben aus der Lunge entnommen. Der Arzt sagte, es sähe eindeutig nach Sarkiodose aus. Die Histologie steht noch aus. Da die Leberwerte zwischendurch auf sehr hoch waren, wurde auch eine Leberpunktion gemacht, das Ergebnis war aber leider unspezifisch.  Mein Mann hat immer wieder Fieberschübe bis circa 39 Grad besonders morgens.  Er wird derzeit seit ein paar Tagen morgens 100 mg und abends 50mg Cortison behandelt. Zusätzlich bekommt er morgens und abends Oxy 20mg gegen die Schmerzen und Ibuprofen circa 4 x 400mg über den Tag gegen die Schmerzen und das Fieber. Der CRP ist bei circa 200. Der ISL 2 Wert war auf 6000 angestiegen.  Der Gesamtzustand scheint sich nicht zu verbessern, sondern eher zu verschlechtern. Wir sind sehr verzweifelt, da trotz Behandlung im besten KH Hamburgs und trotz scheinbarer Diagnose die Cortison Therapie nicht anzuschlagen scheint.  Für jeden Hinweis sind wir dankbar. | Hallo, es macht Sinn, sich einen Arzt zu suchen, der sich wirklich mit dem Krankheitsbild auskennt. Um einen solchen zu finden, sollten Sie sich einmal mit der Selbsthilfe in Verbindung setzen. Dort finden Sie alle nützlichen Informationen und ggf. auch einen Ansprechpartner aus der Region, der Ihnen einen Tipp geben kann. Mit den besten Wünschen. | Es tut mir leid zu hören, dass Ihr Mann krank ist. Aus Ihrer Beschreibung geht hervor, dass er Anzeichen einer Infektion aufweist, zu denen Fieber, Schmerzen, Müdigkeit und Appetitlosigkeit gehören können. Aus Ihrer Beschreibung geht nicht eindeutig hervor, ob der Zustand Ihres Mannes auf eine Infektion zurückzuführen ist, die durch einen bakteriellen, viralen oder pilzlichen Erreger verursacht wird. Diese Symptome können auf ein breites Spektrum möglicher Infektionen hinweisen, darunter Lungenentzündung, Sepsis oder Infektionen im Bauchraum, in den Harnwegen oder im Blut. | Es tut mir sehr leid zu hören, durch welche schwierige Zeit Ihr Mann und Sie gehen. Es klingt so, als ob Ihr Mann eine komplexe und schwerwiegende Erkrankung hat, die möglicherweise Sarkoidose ist. Sarkoidose kann viele Organe betreffen und sich unterschiedlich präsentieren, was die Diagnose und Behandlung schwierig macht. Da Ihr Mann nicht gut auf die Cortisontherapie anspricht, könnte es hilfreich sein, andere immunsuppressive Medikamente in Erwägung zu ziehen. Eine enge Zusammenarbeit mit den Spezialisten im UK Eppendorf und möglicherweise auch die Einholung einer Zweitmeinung könnten weitere Ansätze bieten, um die beste Behandlung für Ihren Mann zu finden. |
| Bei meinem Mann wurde Sarkoidose mit Hyperkalzemie vor einen Jahr festgestellt.  Der Kalzium Wert stieg auf 3,6. - leichte Fibrose in der der Lunge - und leichte Sarkoidose in den Muskeln  Mit Dialyse und Cortison wurde er wieder stabilisiert.   Im Juni wurde Predenisolon dann abgesetzt (Kalcium bei 2,4) - der Vitamin D3 Wert stieg von 19 im Januar auf 60 Ende August, 20 Tage später 110. Dann stopp mit dem D3 Supplementierung 3000IE. Nach 5 Tagen D3 auf 48.  Das Kalzium ist seit Ende August bei 2,8 - 2,99  CRP und ACE sind im niedrigen Normbereich.  Mein Mann ist sehr müde und schläft sehr viel.  Wer kann helfen, was sollte man tun? | Hallo, es scheint, dass die Geschichte ziemlich kompliziert ist. Hat auch ein Nierenversagen vorgelegen? Ist weiterhin eine Dialyse erforderlich? Ich denke, dass man aus der Ferne sicher nicht helfen kann. Es fehlen zu viele Informationen, um festzustellen, ob eine Sarkoidose gesichert oder vermutet wird. Bitte besprechen Sie das mit den behandelnden Ärzten. Wenn dann eine konkrete Frage bleibt, bei der wir helfen können, wenden Sie sich gerne wieder an uns. Beste Grüße. | Sarkoidose und Hyperkalzemie werden nicht oft bei Männern gemeinsam beobachtet. Sowohl eine Sarkoidose als auch die Hyperkalzämie sind Behandlungsmöglichkeiten vorhanden. Die Sarkoidose kann mit Steroiden behandelt werden. Bei Patienten mit Hyperkalzämie wird oft ein intravenöses Kalziumsubstitutionsverfahren und/oder Kalziumantagonisten eingesetzt. Es kann auch eine Dialyse erforderlich sein. Daher sollte Ihr Mann in enger Zusammenarbeit mit einem Arzt, der Sarkoidose behandelt und mit einem Arzt, der Hyperkalzämie behandelt, untersucht und behandelt wird. | Es klingt so, als ob die Sarkoidose deines Mannes trotz der Behandlung weiterhin seinen Calcium- und Vitamin D-Haushalt beeinflusst, was zu seiner Müdigkeit beiträgt. Es ist wichtig, dass ihr euch mit einem spezialisierten Arzt, idealerweise einem Rheumatologen oder Endokrinologen, in Verbindung setzt, um seine Behandlung anzupassen und mögliche weitere Ursachen für seine Symptome zu untersuchen. Regelmäßige Blutkontrollen und eventuell Anpassungen der Medikation könnten notwendig sein, um seine Werte zu stabilisieren und seine Lebensqualität zu verbessern. Ein multidisziplinäres Team könnte helfen, die beste Betreuung für die komplexen Bedürfnisse deines Mannes sicherzustellen. |
| Ich habe Sarkoidose, z.Z. stehen ohne Lungenfibrose... Kann ich mich mit den derzeitigen Corona Impfstoffen impfen lassen? Wenn ja, gibt es Favoriten? | Hallo, es wird auch für Patienten mit Sarkoidose grundsätzlich eine Impfung gegen SARS-CoV-2 (Corona) empfohlen. Eine krankheitsspezifische Empfehlung für die Art des Impfstoffes gibt es nicht, alle in Deutschland zugelassenen Impfstoffe kommen in Frage, wobei allerdings nicht alle Impfstoffe in Deutschland für alle Altersgruppen empfohlen werden. Beste Grüße. | Wenn Sie eine Sarkoidose haben und keine Lungenfibrose aufweisen, können Sie sich mit den aktuell verfügbaren COVID-19-Impfstoffen impfen lassen. Eine Impfung ist auch bei Patienten mit einer Sarkoidose möglich, die zusätzliche Risikofaktoren aufweisen. | Ja, mit Sarkoidose ohne Lungenfibrose können Sie sich in der Regel mit den derzeitigen Corona-Impfstoffen impfen lassen. Es gibt keine speziellen Favoriten unter den Impfstoffen für Personen mit Sarkoidose, aber die mRNA-Impfstoffe wie die von Pfizer-BioNTech und Moderna haben sich als sicher und wirksam erwiesen. Sprechen Sie dennoch mit Ihrem behandelnden Arzt, um Ihre individuelle Situation zu berücksichtigen und die beste Entscheidung zu treffen. |
| Ich habe eine Lungensarkoidose und Brustkrebs mit welchen Impfstoff darf ich mich impfen lassen. Lungenfibrose und Emphysemblasen auf der Lunge habe ich auch noch. | Hallo, für die in Deutschland zugelassenen Impfstoffe gibt es keine Beschränkung bezüglich bestehender Erkrankungen. Grundsätzlich kommen deshalb alle Impfstoffe in Frage, denn inzwischen ist ja auch die Altersbeschränkung für den Astra-Zeneca-Impfstoff zurückgenommen worden. Mit den besten Grüßen | Die Impfung gegen die COVID-19-Erkrankung mit dem Impfstoff Comirnaty (BioNTech/Pfizer) sollte bei Patient:innen mit einer Lungenerkrankung als möglich angesehen werden, die keine schwere Lungenerkrankung aufweisen. Der Impfstoff wurde im Verlauf des Studienprotokolls als sicher und effektiv nachgewiesen. Für Patient:innen mit einer Lungenerkrankung, die eine schwere Lungenerkrankung aufweisen, ist die Impfung mit dem Impfstoff Comirnaty gegen die COVID-19-Erkrankung noch nicht zugelassen. | Bei Ihrer komplexen Krankengeschichte ist es besonders wichtig, Impfentscheidungen in enger Absprache mit Ihren behandelnden Ärzten zu treffen. Allgemein werden Impfstoffe wie die gegen COVID-19 und die Grippe empfohlen, da diese schwere Verläufe verhindern können. Ihre Ärzte können die sicherste Option basierend auf Ihrem aktuellen Gesundheitszustand und den spezifischen Eigenschaften Ihrer Erkrankungen bestimmen. |
| Ich habe seit über 10 Wochen beidseits Beinschmerzen, vom Becken abwärts, überwiegend aber im Sprunggelenksbereich bis Mitte Unterschenkel. Diese sind am stärksten nachts und morgens. Hin und wieder zeigen sich auch Ellenbogenschmerzen. Schlechte Allgemeinsymptome wie Abgeschlagenheit, Schlafstörungen, Leistungsschwäche, Hitzeausbrüche... sind vorhanden.  Mein Hausarzt kann keine Ursache feststellen. | Hallo, grundsätzlich kommt bei den von Ihnen aufgelisteten Beschwerden eine Sarkoidose in Betracht. Es kommen eine ganze Reihe von Erkrankungen in Frage, die ausgeschlossen werden sollten. Es klingt so, dass sinnvollerweise ein Internist einmal schauen sollte, was bereits untersucht und ggf. ausgeschlossen wurde. Beste Grüße. | Die Schmerzen könnte eine Beteiligung des Bewegungsapparates, auch Muskuloskelettal, bedeuten. Bei schmerzhaften Schwellungen, Deformitäten oder Funktionseinschränkungen wird ein Arzt sinnvoll sein. Für diese Situationen gibt es spezialisierte Kliniken. Ihr Arzt könnte Ihnen den Weg dorthin zeigen. | Es tut mir leid zu hören, dass Sie solche Beschwerden haben. Ihre Symptome könnten auf eine systemische Erkrankung wie eine rheumatische Erkrankung oder eine Nervenentzündung hinweisen. Ich empfehle dringend, einen Facharzt wie einen Rheumatologen oder Neurologen aufzusuchen, um weiterführende Untersuchungen durchführen zu lassen. Auch eine Blutuntersuchung und bildgebende Verfahren wie MRT könnten hilfreich sein, um die genaue Ursache zu bestimmen. |
| Ich bin auf der Suche nach einem geeigneten Arzt welcher sich mit Sarkoidose auskennt | Hallo, ich kann Ihnen empfehlen, sich an die Patientenselbsthilfe für Sarkoidosebetroffene zu wenden. Sie können Ihnen unter Berücksichtigung Ihres Wohnortes Auskunft geben. Hier ist der Link zu ihrer Website: https://www.sarkoidose.de/. Beste Wünsche. | Sarkoidose ist eine Erkrankung die auf eine infektiöse oder allergische Reaktion zurückzuführen ist. Es kann eine Vielzahl von Organsystemen betroffen sein, was einer Vielzahl von Symptomen folgen kann. Da diese Erkrankung selten ist und eine Vielzahl von unterschiedlichen Krankheiten die gleichen Symptome haben kann, muss sichergestellt werden dass das gesamte Bild des Krankheitsbildes herangezogen werden muss, um eine Diagnose zu stellen. Der Verdacht einer Sarkoidose sollte immer in einem Krankenhaus eruiert werden. Ein Arzt, der sich mit Sarkoidose auskennt, kann im Krankenhaus den Verdacht gestellt haben oder er sollte mit einem anderen Arzt zusammenarbeiten. | Es ist wichtig, einen Facharzt zu finden, der Erfahrung mit Sarkoidose hat, da dies eine komplexe Erkrankung ist. Ein Lungenfacharzt (Pulmologe) oder ein Facharzt für Innere Medizin mit Spezialisierung auf Rheumatologie oder Immunologie könnte Ihnen weiterhelfen. Sie können bei Ihrem Hausarzt nach einer Überweisung fragen oder sich an eine spezialisierte Klinik wenden. Die Deutsche Sarkoidose-Vereinigung kann auch eine wertvolle Ressource sein, um Spezialisten zu finden. |
| Guten Abend. Am Anfang des Jahres stellte man mir die Diagnose Sarkoidose Lunge mit Granulomen, dann noch eine wahrscheinliche Dermo und Neuro Sarkoidose, jetzt bin ich verzweifelt auf der Suche nach Ärzten undnach Tips, wie man Ärzten erklären kann das Antibiotika eben nicht immer das Allerheilmittel ist, vor allem wenn keine bakterielle Infektion vor liegt, sondern nur eine Entzündung, warum auch immer --- ich bedanke mich schon mal fürs lesen, liebe Grüße | Hallo, in Deutschland gibt es ja glücklicherweise eine größere Zahl an Kollegen, die sich gut mit der Sarkoidose auskennen und die mit Ihnen gemeinsam die Therapie strategisch planen und das auch mit den betreuenden Ärzten vor Ort abstimmen werden. Mit den besten Wünschen. | Guten Abend! Es gibt verschiedene Gründe, warum man Antibiotika bei der Sarkoidose nicht immer verwendet. Zum einen können Antibiotika mit einer Impfung interagieren. Zum anderen gibt es Evidenz, dass Antibiotika einen Einfluss auf das Immunsystem haben und daher einen negativen Effekt haben können. Es gibt keine Evidenz, dass Antibiotika eine Verbesserung von Sarkoidose bringen können. Der Zusammenhang zwischen Antibiotika und Sarkoidose ist eine Forschungsaufgabe, die weiterhin geführt wird. | Guten Abend. Es tut mir leid zu hören, dass Sie diese Diagnose erhalten haben und nun Schwierigkeiten haben, geeignete ärztliche Unterstützung zu finden. Bei Sarkoidose handelt es sich um eine entzündliche Erkrankung, die keine bakterielle Infektion ist und daher nicht mit Antibiotika behandelt werden sollte. Es wäre hilfreich, Ihren Ärzten klar zu machen, dass die Behandlung von Sarkoidose typischerweise entzündungshemmende Medikamente wie Kortikosteroide erfordert und keine Antibiotika. Vielleicht könnten Sie darum bitten, Ihre Diagnose und die empfohlene Behandlung von einem Spezialisten für entzündliche Erkrankungen oder einem Pneumologen überprüfen zu lassen. Liebe Grüße. |
| Ich habe Sarkoidose,Lunge,Haut und Psrotid sind betroffen  Soll ich mich impfen lassen, gegen covid19?  Die Impfung könnte die Sarkoidose verschlimmern und dann ? | Hallo, Sarkoidose stellt keine Kontraindikation für eine Impfung dar, es gilt eine allgemeine Impfempfehlung, von der Sarkoidose auch keine Ausnahme darstellt. Es gibt schon eine große Zahl von Erfahrungen mit Impfungen bei Sarkoidosepatienten, und es gibt keine Hinweise darauf, dass es im Vergleich zu anderen Patienten zu vermehrten Komplikationen oder unerwünschten Nebenwirkungen kommt. Beste Grüße. | Es ist bisher nicht bekannt, ob eine Impfung gegen COVID-19 eine Verschlimmerung der Sarkoidose verursacht. Die COVID-19-Impfung wird als sicher erachtet. Eine Impfung ist daher auch bei Patienten mit einer bestehenden Sarkoidose möglich. Wenn Sie eine Sarkoidose haben und eine Impfung gegen COVID-19 haben, ist es wichtig, Ihnen die Entwicklung einer Sarkoidose zu beachten, und Sie sollten sich jederzeit bei Ihrem Arzt mit Ihrer Sarkoidose und der Impfung besprechen können. Wenn Sie eine Sarkoidose und eine Impfung erhalten, ist es wichtig, regelmäßig eine CT-Untersuchung der Lunge oder eine hochauflösende CT-Untersuchung der Lunge durchzuführen. Eine Entzündungsmedikation sollte vor einer Impfung nicht abgesetzt werden, da dies die Impfantwort reduzieren würde. | Ja, es ist ratsam, sich gegen COVID-19 impfen zu lassen, auch wenn Sie Sarkoidose haben. Während es theoretisch möglich ist, dass die Impfung eine Sarkoidose-Aktivität auslösen könnte, ist das Risiko schwerer COVID-19-Komplikationen höher, besonders wenn Ihre Lunge betroffen ist. Sprechen Sie mit Ihrem behandelnden Arzt, um Ihre individuelle Situation zu bewerten und mögliche Vorsichtsmaßnahmen zu besprechen. Die Vorteile der Impfung überwiegen in den meisten Fällen die potenziellen Risiken. |
| In meinem Wohnort Erfurt bin ich durch meine mittlerweile chron. Sarkoidose in Bhd. bei einem Pneunologen. Allerdings habe ich immer den Eindruck, dass ich dort unzureichend behandelt und teilweise nicht ernst genommen werde. Aus diesem Grunde suche in wohnortnah nach einem Arzt, der sich wirklich allen Aspekten meiner Krankheit widmet. Es ist ja schließlich eine Autoimmunerkrankung, die den Menschen in seiner Gesamtheit schwächt und auch dementsprechend betrachtet werden muß. Ich bin mittlerweile sehr enttäuscht darüber, dass sich jeder Facharzt und seinem Gebiet widmet und nicht in der Lage oder nicht Willens ist ganzheitlich zu arbeiten. Das ewige hin und her Überweisen ist nicht nur sehr umständlich und zeitraubend, sondern auch wenig zielführend. Da es keine ursächliche Therapie gibt und Kortison sehr viele gefährliche Nebenwirkungen mit sich bringt, wäre ich über einen alternativmedizin. Bhd.ansatz dankbar. Das bezahlt aber wiederum die Krankenkasse nicht. Kurzum, hier besteht eine Bhd. lücke im Gesundheitssystem, unter der viele Menschen mit ähnlichen Krankheiten leiden. | Hallo, Sie können gut artikulieren, so dass es möglich sein sollte, einen Arzt zu finden, der Ihren Wünschen nahekommt. Wir können sicher nicht zu einer Empfehlung einer alternativmedizinischen Behandlung raten. Wir sind ja ein Netzwerk, das zur wissenschaftlichen Evidenz beiträgt und deshalb natürlich auch dieser verpflichtet ist. Mit den besten Wünschen | Es tut mir leid zu hören, dass Sie mit der medizinischen Behandlung Ihrer chronischen Sarkoidose in Ihrer Heimatstadt nicht zufrieden sind. Es ist jedoch wichtig zu verstehen, dass es bei der medizinischen Behandlung der Sarkoidose kein Patentrezept gibt, da sich die Krankheit bei verschiedenen Personen auf unterschiedliche Weise manifestieren kann und es keine einzige wirksame Behandlung gibt. Es ist wichtig, dass Sie eng mit Ihren Ärzten zusammenarbeiten, um die beste Vorgehensweise für Ihre spezielle Situation zu bestimmen. Haben Sie versucht, mit Ihren Ärzten über Ihre Bedenken zu sprechen und um eine Überweisung an einen Lungenfacharzt zu bitten, der sich auf die Behandlung von Patienten mit Sarkoidose spezialisiert hat? | Es tut mir leid zu hören, dass Sie mit Ihrer aktuellen Behandlung unzufrieden sind. Es ist verständlich, dass Sie sich eine ganzheitlichere und patientenorientierte Betreuung wünschen. Es könnte hilfreich sein, nach einem spezialisierten Zentrum für Autoimmunerkrankungen oder einem Arzt zu suchen, der integrative Medizin praktiziert. Diese Fachleute betrachten oft den ganzen Menschen und bieten auch alternative Therapien an. Es wäre auch sinnvoll, Ihre Bedenken offen mit Ihrem jetzigen Pneumologen zu besprechen, um gemeinsam eine bessere Behandlungsstrategie zu entwickeln. |
| Gibt es einen Spezialisten für Knochensarkoidose, eventuell ebenfalls für Herzsarkoidose? | Hallo, sowohl die Beteiligung der Knochen an der Sarkoidose als auch die des Herzens ist so relativ häufig, dass in jedem Sarkoidose-Zentrum die dort tätigen Spezialisten als Ansprechpartner empfohlen werden können. Wenn sie ein Sarkoidose-Zentrum in Ihrer Region suchen, können Sie sich an die Patientenorganisation unter www.sakoidose.de wenden. Beste Grüße. | In Deutschland gibt es keine ausreichende Expertise für Knochen‑/Herzsarkoidose. Sie können sich in den folgenden Häusern spezialisierte Behandlung finden: 1. Nationales Zentrum für Sarkoidose in Augsburg 2. Zentrum für Sarkoidose in Göttingen. | Ja, es gibt Spezialisten für Sarkoidose, einschließlich Knochensarkoidose und Herzsarkoidose. Diese Spezialisten sind oft Rheumatologen oder Kardiologen mit Erfahrung in der Behandlung von Sarkoidose. Es kann auch hilfreich sein, ein spezialisiertes Sarkoidosezentrum aufzusuchen, wo multidisziplinäre Teams verfügbar sind. Ihr Hausarzt oder ein Facharzt kann Ihnen bei der Überweisung zu einem geeigneten Spezialisten behilflich sein. |
| Kann eine starke Schuppenflechte bei gleichzeitiger Diagnose einer Sarkoidose der Lunge mit Methotrexat (Metex Pen) behandelt werden? Wenn ja, was ist zu beachten? | Hallo, spezielle Untersuchungen zu der Kombination sind mir nicht bekannt. Die meisten Medikamente sind ohnehin nicht in Ihrer Altersgruppe untersucht, aber es sollte durchaus möglich sein, die Schuppenflechte so zu behandeln, sofern die Dosis vorsichtig ausgetestet wurde. Denn im Alter ist die Knochenmarkempfindlichkeit eher größer, so dass besonders vorsichtiges Vorgehen gerechtfertigt ist. Beste Grüße | Wir empfehlen, die Behandlung mit Methotrexat bei Schuppenflechte mit Sarkoidose der Lunge unter engmaschiger klinischer und laborchemischer Beobachtung durchzuführen und eine Chest X-Ray bei 2 bis 6 Monaten nach Beginn der Therapie durchzuführen. | Ja, Methotrexat kann zur Behandlung einer starken Schuppenflechte verwendet werden, selbst wenn gleichzeitig eine Sarkoidose der Lunge diagnostiziert wurde. Es ist jedoch wichtig, dass Ihr behandelnder Arzt die Therapie genau überwacht, da Methotrexat sowohl Auswirkungen auf die Haut als auch auf die Lunge haben kann. Regelmäßige Blutuntersuchungen und Lungenfunktionsprüfungen sind notwendig, um mögliche Nebenwirkungen frühzeitig zu erkennen und zu behandeln. Stellen Sie sicher, dass Sie alle Anweisungen Ihres Arztes genau befolgen und melden Sie sofort ungewöhnliche Symptome. |
| Ich benötige einen Facharzt in Sachsen oder Sachsen-Anhalt für Sarkoidose. Können sie mir dazu eine Empfehlung geben? | Hallo, tatsächlich haben wir in unserem Netzwerk keinen Kollegen aus der von Ihnen angegebenen Region. Bestimmt finden Sie aber auf den Seiten der Selbsthilfegruppen Ansprechpartner in Ihrer Region, so dass Sie vielleicht dort einmal auf die Suche gehen sollten. Mit den besten Grüßen | Wenn Sie einen Arzt für Sarkoidose benötigen, können Sie sich auf eine Facharztkonsultation für Innere Medizin spezialisiert auf Sarkoidose konzentrieren. Da die Behandlung der Sarkoidose multimodal ist, müssen Sie möglicherweise auch einen dazu fachlich kompetenten Facharzt in einem anderen Bereich des Gesundheitswesens aufsuchen, wie einen Rheumatologen oder Pulmologen. Hier sind Fachärzte auf der Seite des Gesundheitsministeriums von Sachsen und Sachsen-Anhalt vermerkt. | Ich empfehle Ihnen, sich an die Universitätskliniken in Leipzig oder Halle zu wenden, da diese über spezialisierte Abteilungen für seltene Erkrankungen wie Sarkoidose verfügen. Sie können dort kompetente Fachärzte und umfassende diagnostische sowie therapeutische Möglichkeiten finden. Sprechen Sie mit Ihrem Hausarzt über eine Überweisung, um den Kontakt herzustellen. |
| Hallo!  Ich habe sehr wahrscheinlich eine Sarkoidose. Die CT Bilder sind seit ca 2 1/2 Jahren gleich. Es hat sich nichts verändert. Die Punkte sind weder größer geworden noch sind es mehr geworden.  Meine Frage ist nun, ob ich zur Risikogruppe für Corona gehöre, auch wenn ich kein Kortison nehme und die Sarkoidose ruht.  Ich muss im Feb. wieder arbeiten und habe eine Stelle im Kindergarten angeboten bekomme. Wenn ich jedoch gefährdet bin und es auf Grund der Sarkoidose zu einem schweren Verlauf bei Corona kommen kann, würde ich die Stelle nicht annehmen.  Sollte ich mir die Pneumokokken Impfung geben lassen?  Viele Grüße | Hallo, typischerweise behandelt man die Sarkoidose der Lunge nicht, wenn keine Einschränkung der Lungenleistung zu erkennen ist. Wenn dies bei Ihnen der Fall ist, geht solch eine Form der Sarkoidose normalerweise nicht mit einem erhöhten Infektionsrisiko einher. Über Corona wissen wir ja viele Dinge noch nicht, aber aus derzeitiger Sicht besteht auch nicht automatisch ein erhöhtes Risiko für einen schweren Verlauf. Die Ständige Impfkommission empfiehlt eine Impfung bei Menschen ab 60 Jahren. Lungenerkrankungen führen nur zu einer Impfempfehlung bei jüngeren Menschen, wenn die Lungenfunktion beeinträchtigt ist. Mit den besten Grüßen. | Wenn Sie eine Sarkoidose haben, dann haben Sie sich auch einen Behandler und eine Medikation geschaffen. Sie sollten mit Ihrem Arzt sprechen. Der Arzt kann mit Ihnen weiteren Risiken und möglichen Auswirkungen der Sarkoidose auf Corona sprechen und die Empfehlungen geben, die Sie als Patientin mit Sarkoidose einhalten sollten. | Es ist gut zu hören, dass Ihre Sarkoidose stabil ist und sich nicht verschlechtert hat. Bei ruhender Sarkoidose und ohne Kortisonbehandlung ist Ihr Risiko für einen schweren Verlauf von COVID-19 möglicherweise nicht signifikant erhöht, jedoch sollten Sie dies mit Ihrem behandelnden Arzt besprechen, da individuelle Risikofaktoren berücksichtigt werden müssen. Die Arbeit im Kindergarten kann ein erhöhtes Infektionsrisiko mit sich bringen, weshalb eine sorgfältige Abwägung wichtig ist. Eine Pneumokokken-Impfung ist generell sinnvoll, besonders bei Menschen mit chronischen Erkrankungen, und könnte Ihnen zusätzlichen Schutz bieten. Sprechen Sie mit Ihrem Arzt über Ihre individuelle Situation und die besten Vorsorgemaßnahmen. |
| Hallo, bei mir wurde vor 5 Wochen Sarkoidose in der Lunge festgestellt und ich befinde mich angeblich im 3ten Stadium. Typische Anzeichen blieben aus und mein Blutbild weißt auch nicht die für die Sarkoidose typischen Werte auf. Ich war bei einem Lungenarzt bei dem ich mich nicht gut aufgehoben fühle. Dieser meinte nun, dass meine Sarkoidose ausgebrannt zu sein scheint, eine Cortisontherapie wieder abgesetzt werden soll weil wohl keine Besserung zu erwarten sei. Lediglich das Aufflammen der S. gilt es zu verhindern. Tipps und Informationen was und ob ich etwas tun kann, blieben aus. Was sagen Sie du der Diagnose und können Sie mir einen Pneumologen empfehlen?  Vielen Dank | Hallo, typischerweise kennen sich spezialisierte Lungenabteilungen an Unikliniken oder in speziellen pneumologischen Zentren besonders gut aus. Wichtig ist sicherlich, dass ein Behandlungsversuch nach den Regeln durchgeführt wird, bevor man davon ausgeht, dass die Entzündung nicht mehr reagiert. Mit den besten Grüßen. | Die Diagnose der Sarkoidose war nicht sicher. Die Sarkoidose ist eine seltene Entzündungserkrankung der Lunge, welche zu einer Lungentransplantation führen kann . Sie kann auch in der Haut und der Bauchspeicheldrüse auftreten. Die Diagnose wird gestellt, wenn die Körperentzündung 2 oder mehr Biopsien zeigt, welche typische Läsionen haben. | Es tut mir leid zu hören, dass Sie sich in dieser Situation befinden. Sarkoidose kann unterschiedlich verlaufen und die Diagnose im Stadium III ist ernst, aber wenn sie "ausgebrannt" ist, bedeutet das, dass die Krankheit nicht mehr aktiv ist. Dennoch ist es wichtig, regelmäßige Kontrollen durchzuführen, um ein Aufflammen frühzeitig zu erkennen. Einen spezialisierten Pneumologen in Ihrer Nähe zu empfehlen hängt von Ihrem Wohnort ab, aber es wäre ratsam, eine zweite Meinung bei einem erfahrenen Lungenfacharzt einzuholen, idealerweise an einer Universitätsklinik oder einem spezialisierten Sarkoidose-Zentrum. Achten Sie darauf, einen Arzt zu finden, dem Sie vertrauen und der Ihre Fragen ausführlich beantwortet. |
| Last 6.5+ years I have mucus build up in my lungs resulting in shortness of breath, wheezing, multiple coughing fits daily, exhaustion. There is no respite. All tests have come back clear or normal (spirometry (82% ratio), mannitol, allergens, xray, bronchoscopy and biopsy). Incruse COPD inhalher recently added reduces coughing fits a little but not overall problem. Sputum sometimes comes back with result (haemophilius I think), other times clear; multiple antibiotic courses have not made impact, including one month, 1g (i think) amoxicillin, 3x daily. Only abnormality is fatty liver with no relevant symptoms (am obese). I have ME/CFS (16 years) so may be other relevant symptoms masked within that. Diagnosis of asthma as child but only issue was exercising shortnesss of breath; never had proper asthma attack, a couple of chest infections over the years. Salbutamol helps open airways to expel mucus. Mucus is off-white, vaguely green/yellow (darker green with chest infection). Respiratory consultant exhausted options so I'm not necessary looking for diagnosis but what other possibilities to test for or where to look as I am exhausted. GP can't investigate further without knowing what else to look for. | Hello, cough and sputum are what we usually see in our outpatient clinics. You describe the diagnostic procedures that have been done but do not mention a CAT Scan of the chest. This is especially relevant to make sure there is no bronchiectasis (BE). It would be necessary to talk to your treating physician to clarify whether this is an option for you. We certainly cannot make a diagnosis from a distance and it seems a good idea to see a pulmonary specialist. Best wishes | Thank you for your detailed description of your symptoms. I would recommend you make an appointment with your local doctor and ask them to run a simple test to see if you have asthma. They will give you a bronchodilator inhaler and ask you to take it at your next appointment. If you respond with a significant improvement in lung function, then you will be diagnosed with asthma. | I understand how exhausting and frustrating this ongoing issue must be for you. Given the extensive evaluations you've undergone and the persistent nature of your symptoms, it might be worth exploring less common conditions such as bronchiectasis, which can sometimes be subtle on initial imaging, or chronic sinusitis with postnasal drip that could contribute to your lung symptoms. Additionally, considering a referral to a specialized center for a second opinion might provide new insights. Your history of ME/CFS could also be complicating the clinical picture, so consulting with a multidisciplinary team that includes both respiratory and ME/CFS specialists might offer a more holistic approach to managing your symptoms. Keep advocating for yourself and seeking support, as ongoing collaboration with your healthcare providers is key. |
| My question is : which treatment or follow up for a fibrotic pulmonary sarcoidosis patient with recent inflammatory activity ( uveitis/pericarditis) and hugh degree fatigue, muscles weakness and dyspnea with dramatic air privation episodes ( spring / summer) obstructive asthma also and immune condition T / B partial deficiency ? | Hello, for the sarcoidosis manifestations, a treatment with Prednisolon and Methotrexate should be taken into consideration, but after a careful immunological evaluation of the T/B partial deficiency in a specialized center. Best regards | The management of patients with sarcoidosis can be challenging due to its variable natural history and lack of a single defining treatment. Patients with fibrotic pulmonary sarcoidosis typically require supportive care, such as oxygen therapy, and medications to reduce inflammation and manage symptoms. This may include steroids and/or other immunomodulators, as well as physical therapy and pulmonary rehabilitation. Additionally, patients with sarcoidosis should be screened for other potential disease manifestations and complications. It is important for patients with sarcoidosis to work closely with a healthcare team to develop an individualized management plan that addresses their specific needs and addresses potential disease manifestations and complications. | For a patient with fibrotic pulmonary sarcoidosis experiencing recent inflammatory activity, severe fatigue, muscle weakness, and dyspnea, it is crucial to have a multidisciplinary approach. Treatment typically includes corticosteroids or other immunosuppressive agents to control inflammation and prevent further organ damage. Regular follow-ups with a pulmonologist, ophthalmologist, and cardiologist are essential to monitor the sarcoidosis and associated conditions like uveitis and pericarditis. Addressing obstructive asthma with inhalers and managing the immune deficiency with appropriate therapies are also important. Lifestyle modifications, including adequate rest and avoiding known triggers, can help manage symptoms and improve quality of life. |
| After 1,5 year immunosupressant treathment, I developed M. Chiamera infection, large 3-6 cm cavities in lungs, severe fibrosis. After 1 year on the Big3, the infection reactivated with additionaly M. Abscessus. Another 2 years with Big3 helped, and 6 cm large cavity closed. During antitub treatment no progression of fibrosis. Without Big3 fibrosis progressed, and new 7 cm cavity developed. What is treatment suggestion? Sputum is clear. | Hello, we would like to have more information about the details of your situation, i.e. other concurrent treatments, how your overall condition changed, and would like to see the images to get an impression of wall thickness of the cavities as well as inflammatory markers. Is there any information about drug resistance? It would be best to have all the information at hand in case one of the specialists wants to know some details. This is generally best possible if the treating physician is available for a case presentation and discussion. Have you thought of discussing with your doctors, whether they would be interested in such a discussion with some colleagues in Europe? Best regards. | Consider switching to an anti-microbial regimen that includes bedaquiline, clofazamine, amikacin, imipenem/meropenem, and linezolid. This is a combination used in the treatment of both MAC and M. abscessus infections. It is important to consult with a specialist in infectious diseases or pulmonology to ensure that the treatment is appropriate for your condition. | Given your history of severe lung infections and fibrosis, it is crucial to consult with a pulmonologist and an infectious disease specialist. They may consider resuming or adjusting your antibiotic regimen, potentially involving the Big3 or other targeted therapies. Additionally, long-term management might include close monitoring with regular imaging, lung function tests, and possibly adjunctive therapies to manage fibrosis and prevent further progression. It is essential to continue sputum cultures to ensure the infection remains controlled. Your medical team will tailor the treatment plan to your specific needs, balancing infection control with managing fibrosis. |
| Is bronchietcstasis considered a rare disease? | Hi, Bronchiectasis (BE) can be the consequence of different causes. This is why we differentiate CF-related BE and non-CF BE, both are "rare diseases". Best regards | Bronchiectasis is considered a rare disease in general. However, it is more common in certain groups such as people with certain genetic conditions or a history of recurrent respiratory infections. It can be diagnosed at any age, but it is usually diagnosed in adulthood. | Bronchiectasis is not considered a rare disease; it is a chronic condition where the bronchial tubes in your lungs are permanently widened, leading to excess mucus build-up and recurrent infections. While it is less common than conditions like asthma or COPD, it is still relatively prevalent, especially among older adults or those with underlying health conditions. Proper management with medication, physiotherapy, and sometimes surgery can help control symptoms and improve quality of life. |
| Hi there, My mum has IPF and her lung function tests are deteriorating therefore we believe at her next consultant appointment she may be offered anti fibrotic drugs. My mum is concerned about side effects and quality of life on this medication. Can I ask by what percent do the anti fibrotic drugs slow the progression of disease ie will the side effects be worth it for the benefit of drugs?  Thank you | Hi, I think your mum, you, and your treating physician have to weigh between a detoriating irreversible and progressive lung disease which has a significant impact on quality of life on one side and and treatments which have been shown to halt disease progression on the other side. Yes, these drugs may have side effects, but in the process of developing the German guidelines for IPF treatment the patient representative urged us physicians to recommend all symptomatic patients be treated and ishould of course als be informed about effects and side-effects. Taken together as in a severe disease such as IPF the benefits of these drugs available outweigh their side effects. Best wishes. | It is difficult to predict exactly how effective antifibrotic drugs will be for a specific patient, as the rate of progression can vary widely among individuals with IPF. In general, antifibrotic drugs have been shown to slow the decline in lung function and delay the need for lung transplant in IPF patients, but they are not a cure and the progression of IPF is often not stopped completely. | I'm sorry to hear about your mum's condition. Anti-fibrotic drugs, like pirfenidone and nintedanib, have been shown to slow the progression of idiopathic pulmonary fibrosis (IPF) by about 50% in terms of lung function decline. While side effects can occur, such as gastrointestinal issues or skin reactions, many patients find the benefits of slowing disease progression outweigh the potential side effects. It's essential to discuss these concerns with her consultant, who can tailor advice and monitoring to her specific situation. |
| Could you please advise your opinion whether it is not harmful to vaccinate against COVID-19 a person with sarcoidosis of lungs.  The patient is a man, 29 years old. He was diagnosed with sarcoidosis of lungs & lymph nodes back in 2017, stage II. Sarcoidosis was discovered during annual check up, no complaints on health issues. Since then in 2019 and 2020 he had positive dynamics based on results of tomography of lungs and nodes and the local pulmonologist concluded that there are improvements. No treatment was applied since Sept 2018.  Nowadays he would like to get vaccination and his family has doubts whether vaccine against COVID-19 can worsen sarcoidosis dynamics.  Thank you very much. | Hi, it seems that your condition of Sarcoidosis has been favorable and you have not required any treatment. It is generally recommended to receive all necessary vaccinations, including the SARS-CoV-2 vaccine. There is no evidence to suggest that SARS-CoV-2 vaccination poses an increased risk of complications in Sarcoidosis patients. In fact, most experts strongly recommend vaccination for these patients. If your attending physician would like more information, you can share the following paper with them: Manansala M, Chopra A, Baughman RP, et al. COVID-19 and Sarcoidosis, Readiness for Vaccination: Challenges and Opportunities. Front Med (Lausanne). 2021. Best regards. | In patients with sarcoidosis, it is possible that vaccination can lead to the exacerbation of the condition. Therefore, it is recommended for people with sarcoidosis to consult with their healthcare provider before vaccination, and the vaccine should only be administered under the supervision of a healthcare provider with any underlying health conditions. It is also recommended to receive the vaccine at least 2 weeks before starting a new medication or treatment. | Vaccination against COVID-19 is generally recommended for individuals with sarcoidosis, especially if they are stable and not on immunosuppressive therapy, as your family member appears to be. The potential benefits of vaccination, such as protection against severe COVID-19, usually outweigh the risks, especially given his stable condition and positive health dynamics since 2018. However, it's important for him to discuss this with his pulmonologist to ensure personalized advice based on his specific health situation. |
| I have seen a patient with Yellow Nail Syndrome who had been complaining about a continuous dry cough for many years. His Bronchiectasis problem was not the cause of this permanent coghing. All treatments including systemic corticoisteroids etc. failed to improve the coughing until we decided to try Mepolizumab since the Eosinophils were in the range of 6 to 9%. The cough has stopped for almost 6 months now. Do you have any similar experience?  Best regards, | Hi, this sounds like a very specific and very very rare combination of two rare diseases: YNS plus eosinophil Asthma. Best regards. | I am sorry to hear about the long lasting cough of your patient. I assume he has tried the available treatments. I see that Eosinophils have been identified as the cause of the cough. Mepolizumab can help to alleviate the cough. But I also think that Omalizumab might be better than Mepolizumab. Omalizumab is more specific to Eosinophils than Mepolizumab. | Yes, I have encountered cases where Mepolizumab, an anti-IL-5 monoclonal antibody, has effectively treated patients with eosinophilic-driven conditions, including those with persistent cough linked to eosinophilic inflammation. It's encouraging to hear that your patient's cough resolved with Mepolizumab, highlighting its potential benefit in managing eosinophilic-related symptoms. This aligns with the growing recognition of Mepolizumab's efficacy in treating various eosinophilic disorders beyond its primary indications. Continued monitoring and follow-up will be essential to ensure sustained benefits and manage any potential side effects. |
| Hello,  Our son, born 2018 prematurely 29+1 weighing 700 g, has developed very severe BPD. He is now almost 18 months old and his condition has not improved a slightest bit. He is on mechanical ventilation in NICU with high settings on ventilator and FiO2 100%. He has never been off mechanical ventilation and his oxygen requirement have since 3rd day of life always been very high (70-100%). He has been previously on high frequency ocillator and NO. He has had very many courses of steroids: dexamethasone, methylpredinosolone, and is currently on hydrocortisone. He is sedated with dormicum and dexdor for almost 1 year now. His other main daily medications are: atrovent, pulmicort, ACC, Sildenafil, Bosentan, Verospiron. His feeding is partly through nasogastric tube, but most part is parenteral with Numetah.  Our doctors in Estonia have no further ideas how to improve his condition. We would very much appreciate any advice or guidelines how to proceed and help him or recommendations about options where to turn to for help.  We thank you in advance fro any help you can give.  Best regards, | I am sorry that I also have few ideas about how to help your son Albert really. In my opinion, the colleagues have done all options in this severe case of BPD. The medication leaves hardly any effective changes open. I don't know if your son is still intubated or has a tracheostoma. This may help to make ventilation easier. Of course, I can't say more without X-rays. Best regards | I am sorry to hear about the condition of your son. It is certainly not uncommon that patients with BPD require prolonged mechanical ventilation and high dosages of oxygen. Although there is no consensus on a specific definition for “severe BPD”, infants with BPD typically have pulmonary symptoms, including abnormal breath sounds or prolonged oxygen need. Prolonged ventilation is often associated with the development of persistent airway collapse or bronchomalacia . Additionally, chronic oxygen use can lead to long-term pulmonary impairment, such as abnormal lung volumes, diffusing capacity or airway function . The treatment for severe BPD is complex and requires a multidisciplinary approach, including respiratory support, nutritional and pharmacological therapies, as well as physical rehabilitation. Some patients with BPD require prolonged hospitalization and high levels of oxygen. It is important to consider that this group of patients often develops a more severe course of BPD. It is also important to consider the risk of lung damage due to mechanical ventilation. In some cases, a tracheostomy may be performed to help reduce the duration of mechanical ventilation and the need for prolonged hospitalization. One option is to refer the patient to a specialist center for treatment. These centers often have specialized equipment and expertise to treat complex respiratory conditions. Some specialized centers may be located at children's hospitals or have pediatric intensive care units. Another option is to seek a second opinion from a specialist or a different doctor. It is also important to discuss the possible risks and benefits of each option and to make an informed decision based on the patient's needs. | I'm truly sorry to hear about your son's severe bronchopulmonary dysplasia (BPD) and the difficult journey he's been on. Given the complexity and severity of his condition, it would be beneficial to seek a second opinion from a specialized pediatric pulmonary center, particularly those with extensive experience in managing severe BPD. Centers like Boston Children's Hospital in the USA or Great Ormond Street Hospital in the UK are renowned for their expertise in such complex cases. Additionally, it might be worthwhile to explore advanced therapies, including potential novel treatments or participation in clinical trials. Please discuss these options with your current medical team, who can help facilitate referrals and provide the necessary medical records for further evaluation. |
| Ich bin eine Sarkoidose Patientin, meine werte sind erhöht ACE (Angiotensin-converting enzyme) 200.  mein Pomologe sagt mir dass ich keine Lungen Beteiligung habe Stadium 0  Kann sind andere Organen breit von Sarkoidose befallen?  welche Arzt oder wie kann man ich andere Organen Schecken lassen?  Danke sehr im Voraus | Ich gehe davon aus, dass Ihre Sarkoidose durch eine Biopsie (Probe Entnahme) gesichert worden ist. Wenn nicht, sollte man am besten tun. Die ACE Werte können auch bei Sarkoidose komplett im Normbereich liegen. Die Organe, die außerhalb der Lunge von der Sarkoidose am häufigsten beteiligt werden, sind Lymphknoten, Augen, und Haut, selten Herz und das Zentrale Nervensystem. Ihr behandelnder Pneumologe kann Sie zur Zweitmeinung bei einem Sarkoidose Referenzzentrum vorstellen. Wenn Sie eine Anlaufstelle suchen, sollten Sie sich vielleicht mit der Selbsthilfeorganisation in Verbindung setzen. Beste Grüße. | Ich danke Ihnen. Es sieht so aus, als würden Sie fragen, ob auch andere Organe von Sarkoidose betroffen sein können. Wenn der Pneumologe Ihnen gesagt hat, dass die Lunge nicht betroffen ist, dann ist es unwahrscheinlich, dass Sie Sarkoidose haben. Wenn Sie jedoch nicht gründlich untersucht worden sind, ist es möglich, dass Sie Sarkoidose haben. In diesem Fall sollten Sie einen Facharzt für Innere Medizin aufsuchen, der Ihre anderen Organe untersuchen und feststellen kann, ob Sie an Sarkoidose leiden. | Ja, Sarkoidose kann auch andere Organe betreffen, obwohl bei Ihnen keine Lungenbeteiligung vorliegt. Häufig sind Haut, Augen, Herz und Nervensystem betroffen. Um dies zu überprüfen, sollten Sie einen Rheumatologen oder einen spezialisierten Internisten aufsuchen, der eine umfassende Untersuchung und gegebenenfalls spezifische Tests wie MRT oder CT durchführen kann. Ihr behandelnder Pneumologe kann Ihnen auch Empfehlungen geben, an welche Fachärzte Sie sich wenden sollten. Mit freundlichen Grüßen. |
| My niece is 26 years old, since childhood has been diagnosed with asthma and some allergies which are increasing in number with time. She´s been taking so much medication (corticosteroids) that she´s been now diagnosed with osteoporosis. Despite so much corticosteroids the asthma is not controlled. Recently she´s being sick since February, now in hospital again for a week, with light temperature and heavy chest pain, not even able to walk, getting short of breath. During the tests at hospital she´s been in anaphylactic shock twice due to the reaction to the treatment. We DESPERATELY NEED HELP from a great specialist, we do not believe anymore in the doctors that see her since they all "deal" with the matter increasing the doses of corticosteroids, which are obviously harming her and not helping at all. Can you please let me know the name of a specialist near Madrid, Spain, who would be keen to help? Many thanks. | If this girl lives in Madrid I think ""Hospital 12 de Octubre"" would be an excellent option. The respirology department has a specific unit for ""difficult to treat and severe asthma"". The patient could ask to her General Practitioner in Madrid for a derivation to this unit. Hope this helps the patient! | I am sorry to hear that your niece has had such a difficult time with her illness. It sounds like she is experiencing a severe flare-up of her asthma and other allergies, and she has also developed other complications such as osteoporosis as a result of the long-term use of corticosteroids. I understand your frustration with the treatment she has received so far, but it is important to seek a second opinion and potentially try a different approach to managing her condition. As for finding a specialist in Madrid, Spain, I suggest you contact your niece's primary care physician or a local asthma and allergy clinic and ask for a referral to a specialist. They may be able to provide a recommendation for a highly skilled specialist in the area. Additionally, you can search online for allergists or pulmonologists in the Madrid area and look for one that has experience working with difficult-to-treat cases. Please keep me updated on how this is going. | I'm truly sorry to hear about your niece's situation. She urgently needs the attention of a highly specialized allergist and immunologist, preferably one with experience in severe asthma and complex allergic reactions. I recommend seeking help at Hospital Universitario La Paz in Madrid, which has a renowned allergy and immunology department. Dr. Julio Delgado, a leading specialist there, might be a valuable contact. Please ensure her current doctors are informed and work collaboratively to transition her care safely. |
| Ich quäle mich seit Jahren mit wiederkehrenden NNH Entzündungen im Wechsel mit einer immer wieder aufflammenden chronischen Bronchitis. Jetzt ist ein Pleuraerguss dazu gekommen, der nun nach erfolgreicher Punktion vor 4 Wochen erneut und stärker hervortrat. Gelbe Fingernägel habe ich zudem seit 3 Jahren, und gelegentlich fault mal einer ab, wie jetzt zur Zeit. Ödeme im Vorfuß runden das Bild ab. Mein Lungenarzt tippt auf YNS, ist aber im großen Ganzen auch ratlos. Alle anderen Ärzte staunen und sind ohne Strategie.  Gibt es irgendwo Spezialisten, die mein Siechtum koordiniert interdisziplinär angehen, damit die Odyssee zwischen HNO, Pneumo Thorax Chirurgie aufhört? | Ihr Problem ist leider nicht ganz ungewöhnlich, nicht nur bei solch extrem seltenen Erkrankungen wie dem ""Yellow-Nail-Syndrome (YNS)"". Das liegt einerseits daran, dass es kaum Spezialisten gibt, zum anderen aber auch daran, dass selbst die Spezialisten nicht viel anzubieten haben: Wir können beim YNS immer nur reagieren und versuchen, die bestmöglichen Therapieelemente von anderen Erkrankungen für eine symptomatische Therapie zu nutzen. Ich selbst betreue beispielsweise drei Patienten mit YNS - teilweise schon seit sehr vielen Jahren - aber die Probleme sind eben auch sehr unterschiedlich. Wenn Sie eine Email an unser Netzwerk schicken mit Ihrem Standort, kann ich vielleicht einmal schauen, wer in Ihrer Nähe Erfahrung im Management von YNS hat. Beste Grüße. | Ich bedauere, dass Ihre Situation sehr schwer ist. Einige dieser Symptome sind ungewöhnlich und deutlich über dem Niveau eines Allgemeinsyndroms. Entsprechend muss man in der Regel eine Vielzahl an Diagnostiken durchführen, um den zugrunde liegenden Gesundheitsschädigung näher zu charakterisieren. Gerade mit Hautveränderungen kann man nicht sicher ausschließen, dass es sich um einen Hautbefund handelt. Daher sollten Sie mit Ihrem Allgemeinarzt einen Termin vereinbaren, um weiterführende Diagnostiken durchführen zu lassen. Im Fall von Lymphödeme ist es möglich, dass es sich um eine primäre Lymphödemie handelt. Die Diagnose wird nach dem Vorliegen von Genesischen, die durch eine molekularbiologische Untersuchung ermittelt werden können, gestellt. Wenn die Lymphödemie nicht eine primäre Lymphödemie ist, kann die Lymphödemie auf die Nierenfunktion hinweisend sein. Daher sollte ein Nephrologe einen Termin vereinbaren, um die Nierenfunktion genauer zu beurteilen. | Es tut mir leid zu hören, dass Sie mit solch belastenden und komplexen gesundheitlichen Problemen zu kämpfen haben. Der Yellow-Nail-Syndrom (YNS) ist selten und erfordert oft ein interdisziplinäres Vorgehen. Es wäre ratsam, sich an ein Universitätsklinikum oder ein spezialisiertes Zentrum für seltene Erkrankungen zu wenden, wo Fachärzte aus verschiedenen Disziplinen zusammenarbeiten können, um eine umfassende Diagnose und Behandlungsstrategie zu entwickeln. Ein solches Zentrum kann die Koordination zwischen den verschiedenen Fachbereichen übernehmen und sicherstellen, dass Sie eine ganzheitliche Betreuung erhalten. |
| I understand PPFE is a rare ILD. Is there any reason PPFE patients should not be included in other studies for PF/ILD research? Is there a huge difference as to the fibrosis of other PF and PPFE? | Indeed, PPFE differs much from idiopathic pulmonary fibrosis (IPF) and other types of pulmonary fibrosis. There are huge differences in regards to the cellular composition of the fibrosis. There is no evidence that any approved antifibrotic treatment works for PPFE and several case reports suggest that there is probably no benefit of approved antifibrotic treatment. Therefore, it is not reasonable to include PPFE patients in clinical trials testing new compounds for IPF. Unfortunately there is no evidence so far that any treatment works in PPFE. Avoidance of any infections and weight loss is recommended. | Patients with PPFE are often excluded from studies on pulmonary fibrosis because of the overlap in clinical features with other interstitial lung diseases and because they are difficult to diagnose. 1 However, they are a unique disease entity with distinct pathological and radiological features, and recent studies have shown that patients with PPFE have specific molecular genetic abnormalities and respond to targeted therapies differently than patients with other types of pulmonary fibrosis. 2 PPFE is characterized by diffuse thinning of the pleura with subpleural lung cysts, and is often associated with other interstitial lung diseases, such as idiopathic pulmonary fibrosis or sarcoidosis, which can make diagnosis challenging. It is important for researchers to consider including patients with PPFE in studies on pulmonary fibrosis to better understand the disease and develop effective treatments. | Pleuroparenchymal fibroelastosis (PPFE) is indeed a rare form of interstitial lung disease (ILD) that differs in its specific pattern of fibrosis, primarily affecting the pleura and upper lobes with an elastotic component. While its unique characteristics distinguish it from other forms of pulmonary fibrosis, including PPFE patients in broader PF/ILD studies could still provide valuable insights, given the overlapping features and potential shared mechanisms of fibrosis. However, it's crucial to consider the distinct clinical course and treatment responses of PPFE, which may necessitate tailored research approaches. |
| Sehr geehrte Damen und Herren, welche Erfahrungen gibt es bei dem Medikament Inflximab bei einer bestehenden Sarkoidose. Gibt es Erfahrungen und wie sehen die aus? | Infliximab gehört sicher zu den wichtigen Kandidaten, wenn es darum geht, in Zukunft die Therapiemöglichkeiten bei Sarkoidose zu erweitern. Derzeit gibt es noch keine Leitlinienempfehlung einer Behandlung mit dieser Substanz, so dass eine Therapie damit als off-label-use nur in Frage kommt, wenn die Behandlungen mit Cortocosteroiden, Methotrexat und/oder Azathioprin nicht erfolgreich ist oder zu unzumutbaren Nebenwirkungen führt. Es gibt meines Wissens noch keine publizierte kontrollierte Studie zu dem Thema, wohl aber positive Fallberichte. Hierbei ist aber auch zu bedenken, dass die Ergebnisse von Behandlungserfolgen bei der Neuro-Sarkoidose nicht automatisch auf die Behandlung der Lungen-Sarkoidose zu übertragen sind. Sie sollten sich unbedingt in einem Sarkoidose-Zentrum vorstellen, wo Erfahrung mit allen Formen der Sarkoidose und allen Formen der Therapie vorliegen. Mit den besten Grüßen. | Infliximab ist ein Medikament, das häufig zur Behandlung von entzündlichen Erkrankungen wie rheumatoider Arthritis, Colitis ulcerosa, Psoriasis, psoriatischer Arthritis und Morbus Crohn eingesetzt wird. Es gibt nur wenige Studien über den Einsatz von Infliximab bei der Behandlung von Sarkoidose. Die wichtigste Studie, die ich gefunden habe, stammt aus dem Jahr 2009 und umfasste 14 Patienten mit refraktärer Sarkoidose. Diese Studie zeigte, dass Infliximab bei der Behandlung von Sarkoidose wirksam war, wobei bei 12 der 14 Patienten eine deutliche Verbesserung der Symptome zu verzeichnen war. Allerdings kam es auch zu einem Anstieg der Eosinophilenzahl im Blut dieser Patienten. Dieser Anstieg des Eosinophilenspiegels ist ein potenzielles Risiko bei der Verwendung von Infliximab zur Behandlung von Sarkoidose, da Eosinophilie eine ernsthafte Nebenwirkung sein kann. Darüber hinaus bestand das Risiko einer Hepatotoxizität und einer Lymphopenie. | Infliximab wird gelegentlich bei schwerer, therapieresistenter Sarkoidose eingesetzt, insbesondere wenn andere Behandlungen nicht erfolgreich waren. Es gibt Berichte über positive Erfahrungen, insbesondere bei Sarkoidose mit pulmonalen und extrapulmonalen Manifestationen. Patienten zeigen oft eine Reduktion der Entzündungszeichen und eine Verbesserung der Symptomatik. Dennoch ist es wichtig, die Therapie sorgfältig abzuwägen und mögliche Nebenwirkungen zu überwachen, da Infliximab das Immunsystem beeinflusst. Sprechen Sie mit Ihrem behandelnden Arzt, um die beste Behandlungsstrategie für Ihren individuellen Fall zu bestimmen. |
| I have been a sarcoidosis patient since December 2017 and since last year the symptoms (shortness of breath, extreme fatigue, heart problems, muscle pains) have increased. All organs have been tested and except for the lungs, these have not been affected. According to the studies, the immune system should overcome the disease after 18 months, but this is apparently not the case for me. The prednisone treatment has also not helped and has actually done more damage to my body (weight gain, muscle breakdown).  Is there an alternative treatment method that could improve my quality of life? | It is true that in the vast majority of patients with sarcoidosis, the findings improve over time or they disappear completely, but unfortunately, not in some patients. Corticoid therapy generally is the first choice, but this often is accompanied be untoward effects. There are a number of therapeutic approaches both to save corticosteroids or to replace it in the case of intolerance, some of which are not as established as cortisone therapy itself. Therefore, such therapy supplements or alternatives are preferably prescribed in sarcoidosis centers. If you do not know where to find such a center, you should go fat the national sarcoidosis patient organization or you are welcome to be back here. All the best. | It sounds like a very difficult situation you are in. I'm sorry to hear that the prednisone treatment has caused more harm than good. It is difficult to give general advice without access to your specific medical file, but in general, steroid therapy is usually tapered down and stopped after 12-18 months, so it sounds like it's past that timeline. The first thing I would recommend is to contact your doctor to see if she would be willing to work with you to find an alternative treatment. It is important to note that steroid withdrawal can also have serious side effects, including a potentially dangerous adrenal insufficiency, so this process should be carefully monitored by your doctor. Other possible treatments for sarcoidosis include immunosuppressants like methotrexate and mycophenolate mofetil, biologics like infliximab and abatacept, and even radiation therapy. However, these treatments may have their own side effects and may not be appropriate for you given your specific situation. Therefore, it is important to work closely with your doctor to find the best treatment plan for you. | I'm sorry to hear about the challenges you're facing with sarcoidosis. Since prednisone has not been effective and has caused side effects, other treatments such as methotrexate, azathioprine, or infliximab might be considered. Additionally, pulmonary rehabilitation can improve lung function and overall quality of life. It's important to work closely with a specialist who can tailor a treatment plan to your specific condition. Please feel free to share your medical file with your healthcare provider for a more detailed review and personalized advice. |
| Gibt es einen Zusammenhang zwischen sarkoidose und Lymphstau? | Ja, die Sarkoidose kann auch zu Lymphstau in den Beinen, Armen oder im Bauchraum oder Rippenfellspalt führen. Es handelt sich um eine Systemerkrankung, die praktisch alle Organe betreffen kann. | Lymph stasis (auch Lymphödem) kann mit Sarkoidose assoziiert sein. Die Lymphgefäße können im Rahmen einer Sarkoidose verändert sein und die Lymphflüssigkeit kann dadurch gestaut sein. Dies kann zu einem schleuderartigen Erguss und zu einer Schwelling der betroffenen Gegend ist. | Ja, es gibt einen Zusammenhang zwischen Sarkoidose und Lymphstau. Sarkoidose kann eine Entzündung der Lymphknoten verursachen, was zu einer Blockade und einem Rückstau von Lymphflüssigkeit führt. Dies kann Schwellungen und andere Symptome verursachen, die mit einem Lymphstau verbunden sind. Es ist wichtig, diese Symptome mit Ihrem Arzt zu besprechen, um eine angemessene Behandlung zu erhalten. |
| Welche Medikamente kann man nehmen außer MTX und Cortison? Ich nehme seit 3 Monaten MTX und ständig kommen die Schübe wo ich dann zusätzlich Cortison nehmen muß. In regelmäßigen Abständen muss ich dann sogar stationär aufgenommen werden da mein Kehlkopf so doll anschwillt und ich sehr schlecht Luft bekomme. Meine Frage gibt es vielleicht andere Medikamente die wirklich helfen? Vielleicht auch die in Deutschland nicht zugelassen sind. | Ja, es gibt weitere Immunsuppressiva, die als Alternative zu Cortison und MTX genommen werden können, wie zum Beispiel Azathioprin, Leflunomide, Mycophenolat Mofetil und in Drittline die Biologika. Da die Entscheidung Einzelfall-basiert ist, wäre es sinnvoll, das Sie sich an ein Referenzzentrum für die Behandlung der Sarkoidose wenden. Studien mit neuen Substanzen können in Referenzzentren angeboten werden.  Auf der Suche nach einem Referenzzentrum"in Ihrer Nähe können Sie sich an die Selbsthilfe wenden, dort finden Sie alle hilfreichen Informationen. Beste Grüße | In Deutschland sind zur Behandlung der RA vorrangig Biologika wie Anti-TNF-Alpha-Antikörper, IL-6-Rezeptor-Antagonisten, Anti-CD20-Antikörper sowie JAK-Inhibitoren verfügbar. Zudem gibt es einen TPMT-Test zur Optimierung der Therapie mit MTX. In der Therapieplanung sollte der Schmerz als guter Surrogatparameter eine entscheidende Rolle einnehmen. Eine rezente Cochrane-Analyse hat ein neues Interventionsansatzes in die RA-Therapie eingeführt, das „treat-to-target“-Modell. In einer systematischen Literaturanalyse konnte gezeigt werden, dass ähnliche Behandlungsstrategien zur Senkung der Krankheitsaktivität wirken und die Behandlungsansätze, die im „treat-to-target“-Modell genutzt werden, auch in den Biologika- und JAK-Inhibitoren-Behandlungsversuchen erfolgreich eingesetzt wurden. Im Behandlungsverlauf sind regelmäßige Reevaluationen unabdingbar. | Es tut mir leid zu hören, dass Sie solche Schwierigkeiten haben. Es gibt mehrere Alternativen zu MTX und Cortison, wie Biologika (z.B. TNF-Hemmer, IL-6-Hemmer) oder JAK-Inhibitoren, die oft bei rheumatischen Erkrankungen eingesetzt werden. Diese Medikamente können in Deutschland zugelassen sein, aber Ihr behandelnder Arzt kann Ihnen am besten helfen, die passende Therapie für Sie zu finden. Es ist wichtig, dass Sie Ihre Symptome und Behandlungsmöglichkeiten ausführlich mit Ihrem Arzt besprechen, um eine maßgeschneiderte und effektive Lösung zu finden. |
| Guten Tag  gibt es zur Kortisonbehandlung alternative Therapiemöglichkeiten, auch andere Medikamente, bei chronischer Sarkoidose und gleichzeitiger Diabetes Typ II, da die Einnahme von Kortison die Zuckerwerte kaum kontrollierbar macht?  Vielen Dank | Ja, man kann versuchen, die durch Kortison erreichten Therapie-Effekte auch mit anderen Medikamenten zu erreichen. Als ersten Schritt muss man aber mit dem betreuenden Sarkoidosespezialisten prüfen, ob überhaupt eine Indikation für eine dauerhafte Therapie erforderlich ist. Auch muss man prüfen, ob eine Kortison-Dosis, die den Zucker durcheinander bringt, angemessen ist. Oft wird zwar der Insulinbedarf durch Corticoide erhöht, aber dass der Zucker dann unberechenbar wird, ist eher ungewöhnlich, so dass man auch nach anderen Gründen für ein Durcheinander suchen muss. Steht eine Behandlungsnotwendigkeit außer Frage, kann ein Versuch mit Azathioprin gerechtfertigt sein, das zumindest bezüglich der Zuckereinstellung unproblematisch ist. Beste Grüße. | Ja, natürlich gibt es alternative Therapiemöglichkeiten bei Kortison bei chronischer Sarkoidose mit Diabetes Typ II. Wir haben zahlreiche Behandlungsoptionen für Kortison in der Literatur und in Studien, die wir verwenden können, wenn Kortison eine nicht gewünschte Nebenwirkung zeigt. Daher ist die Therapie individuell festzulegen und abhängig von der Schwere der Krankheit. | Ja, es gibt alternative Behandlungsmöglichkeiten für chronische Sarkoidose, besonders bei gleichzeitiger Diabetes Typ II. Methotrexat und Azathioprin sind zwei nicht-steroidale Immunsuppressiva, die oft als Alternativen zu Kortison verwendet werden. Auch Antimalariamittel wie Hydroxychloroquin können in bestimmten Fällen hilfreich sein. Besprechen Sie diese Optionen ausführlich mit Ihrem behandelnden Arzt, um die beste Therapie für Ihre spezifischen Bedürfnisse zu finden. |
| Gibt es in Deutschland Spezialisten, die sich mit der Sarkoidose des Herzens auskennen?  Wie wahrscheinlich ist es, dass bei einem Schub, das bislang betroffene Organ (Herz) erneut betroffen ist? | Ja, natürlich gibt es auch solche Spezialisten in Deutschland. Ob es Sinn macht, Sie bei einem solchen speziell an der Sarkoidose des Herzens interessierten Kollegen vorzustellen, müssten Sie mit Ihrem betreuenden Pneumologen besprechen. Dieser kann sich gerne an uns wenden. Zu Ihrer zweiten Frage kann man allgemein sagen, dass bei einer Reaktivierung oder einem Rezidiv der Sarkoidose, das Muster der Organbeteiligung meist dem der vorausgegangenen Episode entspricht, manchmal kommen Organe hinzu, dass betroffene Organe ausgespart belieben, ist eher ungewöhnlich. Mit den besten Wünschen | Es gibt in Deutschland Spezialisten, die sich mit der Sarkoidose des Herzens auskennen. Die Wahrscheinlichkeit eines erneuten Schubs, das betroffene Organ zu erneut betroffen zu sein, ist je nach Krankheitsverlauf und Krankheitsaktivität unterschiedlich. Es sollte immer medizinisch kontrolliert werden. Wie lange sie eine Therapie erhalten müssen und ob eine Rezidivprophylaxe empfohlen wird, kann von dem betroffenen Patienten mit seiner individuellen Krankheitsaktivität und Krankheitsverlauf abhängen. Es wird empfohlen, mit einem Fachspezialisten zusammenzuarbeiten. | Ja, in Deutschland gibt es Spezialisten für Herzsarkoidose, meist in größeren Universitätskliniken oder spezialisierten Zentren für Lungenerkrankungen. Bei einem Schub der Sarkoidose ist es durchaus möglich, dass das bereits betroffene Organ, in diesem Fall das Herz, erneut betroffen ist. Eine regelmäßige Überwachung und frühzeitige Behandlung sind wichtig, um Komplikationen zu vermeiden. Es wäre ratsam, sich an eine spezialisierte Klinik oder einen erfahrenen Kardiologen zu wenden. |
| Wenn bei den diversen durchgeführten Untersuchungen inc. Lungenbiopsie, Lumbalpunktion, CT, MRT, etc. keine Granulome beim 69-jährigen Patienten gefunden wurden, aber die Ärzte trotzdem eine (Neuro)- Sarkoidose vermuten, wie kann man diese zu 100% eindeutig diagnostizieren? Im Raum steht eine längerfristige Cortison Therapie. Wenn es am Ende doch keine Sarkoidose ist und die Nebenwirkungen von Cortison nicht unerheblich sind, möchte der Patient VOR Beginn der Therapie 100% sichergehen, dass die Diagnose Sarkoidose auch wirklich korrekt ist. Welche Möglichkeiten gibt es, diese seltene Krankheit mit 100%iger Sicherheit festzustellen bzw. welche andere Alternativen kämen in Frage? | Lieber Patient, relativ häufig kann man eine Neurosarkoidose nicht zu 100% sichern. Die Biopsie ist sehr schwierig, die Liquor Analyse kann helfen (zeigt Lymphozytose und erhöhte ACE Wert) aber ohne Biopsie bleibt die Sarkoidose nur „wahrscheinlich"" . Da die häufige alternative Diagnose Multiple Sklerose ist, kann eine Cortisontherapie nicht schaden. Wenn die Symptome ausgeprägt sind, würde ich seitens des Patienten nicht zögern. Die Neurosarkoidose ist gefährlich und kann zu permanentem Schaden führen, genauso wie die MS. Anbindung an ein Sarkoidose-Zentrum wird empfohlen. | Zunächst einmal muss zwischen Sarkoidose und anderen granulomatösen Erkrankungen unterschieden werden. Dies kann anhand des klinischen Kontextes, des Vorhandenseins extrapulmonaler Manifestationen, des Fehlens infektiöser Risikofaktoren, des Vorhandenseins ophthalmischer Manifestationen und des HLA-Klasse-II-Antigens sowie des Fehlens einer Lymphom-Familienanamnese erfolgen. Der diagnostische Goldstandard ist nach wie vor die histopathologische Untersuchung von Biopsieproben. Es ist wichtig, Proben von verschiedenen Stellen zu entnehmen, darunter Lymphknoten, Hautläsionen, Augen-, Lungen- und Herzgewebe, um zwischen den verschiedenen Arten granulomatöser Erkrankungen unterscheiden zu können. Weitere Tests, die zur Unterstützung der Sarkoidose-Diagnose eingesetzt werden können, sind der Serumspiegel des Angiotensin-konvertierenden Enzyms, die CT-Bildgebung des Brustkorbs und die Gallium-Szintigraphie. Es ist wichtig zu beachten, dass es keinen einzelnen diagnostischen Test gibt, mit dem die Sarkoidose mit absoluter Sicherheit diagnostiziert werden kann, und dass die Diagnose oft eine Kombination von Tests erfordert. Neben der Sarkoidose sollten auch andere granulomatöse Erkrankungen wie Tuberkulose und mykobakterielle Infektionen in Betracht gezogen werden. | Eine 100%ige Sicherheit bei der Diagnose von Sarkoidose ist schwierig zu erreichen, da sie oft eine Ausschlussdiagnose ist. Eine Kombination von klinischen Symptomen, Bildgebung und histologischen Befunden wird normalerweise verwendet. Wenn keine Granulome gefunden wurden, könnten spezifische Bluttests wie das Angiotensin-Converting-Enzym (ACE) oder die Lysozym-Werte hilfreich sein. Weitere Spezialuntersuchungen wie eine Positronen-Emissions-Tomographie (PET-Scan) oder Gallium-Szintigraphie können ebenfalls Aufschluss geben. Es wäre ratsam, eine zweite Meinung von einem Spezialisten für seltene Krankheiten oder eine erneute Biopsie in Erwägung zu ziehen, bevor eine längerfristige Cortisontherapie begonnen wird. |
| Every now and then in our patient group people report that they or their child had more cough and felt bad but when the doctor listened at the chest he said: "It cannot be so bad, I cannot hear anything." So we in the group have the impression that in PCD it is often a bad sign if the doctor does not hear anything. It is better if he/she hears a "washing-machine-sound". Is this also your experience and do you have an explanation for this phenomenon?  (Should patients and treating physicians be informed about this?) | Most likely, this question relates to pulmonary exacerbations. The PCD Core Network is currently working on a definition of 'pulmonary exacerbation'. Obviously, increased cough frequency, shortness of breath, impaired general condition, increased sputum production, and/or fever can indicate pulmonary exacerbation and antibiotics can be started regardless whether auscultation is normal or not. In asthma patients we consider the phenomenon of a 'silent chest', a condition when neither a normal breathing sound nor wheezes can be heard, as an indicator of very severe asthma. This among doctors is a well known phenomenon and warning sign, which can be explained by the fact that in severe asthma the air flow in the obstructed airways is reduced due to exhaustion of the breathing muscles and thus mucus isn't vibrating anymore. It is important though, to understand that most doctors who say "I cannot hear anything" when doing an auscultation indicate that they do not hear any pathological crackles or wheezes although they hear the normal breathing sound. | This is an interesting point to consider, and it is true that a physician with expertise in PCD would be more attentive to subtle cues that are suggestive of PCD. However, a lack of finding air-trapping or crackles does not rule out the possibility of PCD. There are other characteristics in PCD such as increased end-inspiratory lung volumes and airway collapse at end-expiration that can be indicative of the disease. It is important for patients and caregivers to understand that it is difficult for a physician who may not be well-versed in PCD to definitively diagnose or exclude PCD based solely on chest auscultation. Therefore, it is important to have a strong suspicion of PCD and to seek a referral to a physician with expertise in PCD. | In Primary Ciliary Dyskinesia (PCD), lung sounds can sometimes be deceptive. A lack of audible abnormal sounds might indicate mucus buildup that is not moving, which can be a sign of poor clearance and potential infection. The "washing-machine sound" typically indicates movement of secretions, which, while abnormal, can be easier to manage and treat. It is crucial for both patients and physicians to be aware that the absence of sound doesn't necessarily mean there is no problem, and regular, thorough evaluations are important for proper management. |
| Ich bin weiblich 24 Jahre alt und hab ungefähr seit 2 Jahren geschwollene Lymphknoten die sich immer mehr häufen an den verschiedensten Stellen (Hals, Achsel, Bauch, Unterarm, Leistenbereich,..) hinzu kommt das ich des öftern Atemprobleme habe und meine Schleimhäute (Nase und Mund) sehr schnell extrem austrocknen. Mein Vater und mein Bruder hatten beide einen akuten Verlauf von Sarkoidose. Daher stellt sich mir die Fragen ob ich evtl. einen chronischen Verlauf habe. Und ja ich war schon beim HNO und Hausarzt, diese konnten aber nicht wirklich eine Diagnose stellen. | Natürlich haben Sie völlig richtig überlegt, wenn Sie sich fragen, ob Lymphknotenschwellungen nicht etwas mit einer Sarkoidose zu tun haben können. Allerdings sind solche Vergrößerungen der Lymphknoten nicht ausschließlich bei Sarkoidose zu finden – und die Verteilung ist nicht so typisch. Ob die gelegentliche Luftnot nun etwas damit zu tun hat, kann ich aus der Ferne natürlich auch nicht sagen. Für die Sarkoidosefrage macht man ja typischerweise mal ein Rö-Bild. Das hilft dann schon in den meisten Fällen weiter. Fragen Sie noch einmal bei Ihrem Hausarzt nach, denn es gibt eine Menge Dinge zu beachten wie Größe, Schmerzhaftigkeit, Verschieblichkeit der Lymphknoten, aber auch Blutwerte können helfen. Mit den besten Grüßen. | Es hört sich an, als ob Sie Sarkoidose haben könnten. Sarkoidose kann geschwollene Lymphknoten verursachen, insbesondere im Nacken, in den Achselhöhlen und in der Brust, sowie Atemprobleme wie Kurzatmigkeit und trockenen Husten. Sie kann auch andere Organe wie die Augen, das Herz, die Haut und die Knochen betreffen. Wurden Sie schon auf Sarkoidose getestet? | Es ist verständlich, dass Sie sich Sorgen machen, besonders mit Ihrer Familiengeschichte. Die Symptome, die Sie beschreiben, können tatsächlich mit Sarkoidose zusammenhängen, aber auch auf andere Erkrankungen hinweisen. Da sowohl Ihr Vater als auch Ihr Bruder an Sarkoidose erkrankt sind, wäre es ratsam, einen Spezialisten aufzusuchen, wie einen Pneumologen oder einen Rheumatologen, um eine gründlichere Untersuchung und spezifische Tests wie eine Biopsie oder bildgebende Verfahren durchzuführen. Ihre anhaltenden Symptome und Familienanamnese rechtfertigen definitiv eine weiterführende Abklärung. |
| Welche nächsten Schritte empfohlen? Gibt es Referenzfaelle?  1. Perikarderguss vor 6 Monaten als Zufallsbefund diagnostiziert, seitdem stabil (15-20 mm circumferent)  2. Herz selbst in MRT ohne Entzündung / Vernarbung  3. Auf Lunge in 2005 bereits Verkalkung entdeckt, damals als verheilte TBC diagnostiziert  4. Allerdings aktueller Quantiferontest negativ und in Thorax CT deutliche Verkalkungen der Lymphknoten -> Befund Sarkoidose?  5. Blutbild Nefrologie und Rheumatologie ohne Befund  6. Warum ist Perikarderguss zurückgeblieben und wird nicht resorbiert (zumindest seit 6 Monaten stabil)? Bzw was verursacht ihn weiterhin?  7. Ärzte schlagen Thorakoskopie der gesunden Lymphknoten und/oder Punktieren des Perikarderguss als nächsten Diagnoseschritt vor. Was ist am vielversprechendsten?  Vielen Dank! | Natürlich können wir auf diesem Wege keinen individuellen Diagnostik-Plan aufstellen oder raten, was im Einzelfall zu tun ist. Das kann nur ein Arzt, der den Patienten kennt. Typischerweise würde man versuchen, mit dem am wenigsten belastenden Verfahren die Lymphknoten zu biopsieren und zu untersuchen, damit man die Sarkoidose sichern kann. Ein Perikarderguss bei Sarkoidose ist bekannt und sollte therapiert werden. Eine erneute Punktion ist nicht notwendig, falls eine Histologie-Sicherung besteht. Beste Grüße. | Am besten ist die Thorakoskopie der gesunden Lymphknoten zu empfehlen, da sie nicht nur das Vorkommen eines Sarkoidosisbefalls belegen kann, sondern auch eine histologische Untersuchung erlaubt, um andere Diagnosen, wie bspw. ein Adenokarzinom zu ausschließen. Die Thorakoskopie ist auch als Punktionsverfahren in der Diagnose der Perikarditis hilfreich, wenn die Perikarditis nicht sicher von einer Perikarditis-verursachenden Infektion unterschieden werden kann. | In Ihrem Fall könnte eine Thorakoskopie der verkalkten Lymphknoten und/oder eine Punktion des Perikardergusses wertvolle diagnostische Informationen liefern. Der Perikarderguss könnte durch die zugrunde liegende Sarkoidose verursacht werden, auch wenn andere Ursachen wie Infektionen oder autoimmune Prozesse weiterhin in Betracht gezogen werden müssen. Ein stabiler Perikarderguss über sechs Monate ist ungewöhnlich und rechtfertigt eine genauere Untersuchung. Die Thorakoskopie könnte helfen, die Natur der Verkalkungen und die genaue Ursache des Perikardergusses zu bestimmen. Es ist wichtig, die potenziellen Risiken und Nutzen dieser invasiven Verfahren mit Ihren behandelnden Ärzten zu besprechen, um die bestmögliche Entscheidung zu treffen. |
| Mein Mann hatte ein 7cm langes Granulom in der Tränendrüse und Orbita Re.Auge.Dadurch eine hängendes Oberlid.Ursache sei Sarkoidose.  Granulom würde entfernt.Nach OP am 31.01.2019 ständig Bindehautentzündung, Doppelbilder,Lichtempfindlichkeit Kratzen im Auge und Schmerz. BEKOMMT Bepanthen und Hyularongel...nun seien viele Löcher in der Hornhaut und starke Schmerzen.Augenärzte in der AMB.Uni Mannheim sagen es sieht gut aus..und das Auge ist stark gerötet mittlerweile mit Eiterbildung. Kein Arzt fühlt sich zuständig Oder scheint ...nicht kompetent für Sarkoidose.  Wir suchen nun verzweifelt einen Kompetenten Arzt für Augensarkoidose.  Können sie uns helfen...bitte.  Mit freundlichem Gruß | Ophthalmologen mit Interesse an Sarkoidose kenne ich keine. Sind noch andere Organe betroffen? Falls nicht, ist die Diagnose zu hinterfragen. Ein Therapieversuch mit systemischen Corticosteroiden (Prednisolon) scheint, eventuell unter Antibiotikaschutz, angemessen. „Monoorgansarkoidosen“ außerhalb des Thorax haben häufig ungewöhnliche Manifestationsformen und sind von der allgemein akzeptierten Definition nicht als Sarkoidose erfasst. Mit den besten Grüßen. | Es tut mir leid zu hören, dass Sie mit der Augenerkrankung Ihres Mannes eine komplexe und schwierige Situation zu bewältigen haben. Sarkoidose am Auge ist eine seltene und schwierig zu behandelnde Erkrankung, und es kann frustrierend sein, wenn man das Gefühl hat, dass man nicht die Hilfe bekommt, die man braucht. Hier sind einige Vorschläge, die Ihnen helfen könnten, die nötige Unterstützung zu erhalten: Zunächst sollten Sie in Erwägung ziehen, eine zweite Meinung von einem Spezialisten einzuholen, der sich auf die Behandlung der okulären Sarkoidose spezialisiert hat. Dies könnte Ihnen helfen, die Erkrankung Ihres Mannes und die verfügbaren Behandlungsmöglichkeiten besser zu verstehen.  Zweitens sollten Sie versuchen, einer Selbsthilfegruppe beizutreten oder sich an Patientenorganisationen zu wenden. Diese Gruppen können Ihnen emotionale Unterstützung bieten und Ihnen helfen, sich mit anderen auszutauschen, die ähnliche Herausforderungen erlebt haben. Sie können auch eine wertvolle Quelle für Informationen und Hinweise zum Umgang mit der Erkrankung Ihres Mannes sein. Falls Sie dies noch nicht getan haben, würde ich Ihnen empfehlen, den Arzt Ihres Mannes zu konsultieren und ihn zu bitten, Sie an einen Spezialisten für Sarkoidose zu überweisen. Er kann Ihnen dabei helfen, die beste Vorgehensweise zu bestimmen und Sie mit den entsprechenden Ressourcen in Verbindung bringen, damit Sie die benötigte Hilfe erhalten. | Es tut mir leid zu hören, dass Ihr Mann solche Schwierigkeiten hat. Es ist wichtig, einen spezialisierten Augenarzt oder eine Klinik zu finden, die Erfahrung mit Augensarkoidose hat. In Deutschland gibt es einige spezialisierte Zentren, wie zum Beispiel die Augenkliniken in großen Universitätskliniken (z.B. in Freiburg, München oder Berlin). Sie können auch eine Überweisung zu einem Rheumatologen in Erwägung ziehen, da Sarkoidose systemisch behandelt werden muss. Ich empfehle dringend, sich an diese spezialisierten Zentren zu wenden, um eine umfassende und spezialisierte Behandlung zu erhalten. Gute Besserung für Ihren Mann! |
| Können sie mir Spezialisten nennen für das Spezialgebiet Sarkoidose die im Raum Niedersachsen / Hannover praktizieren? | Sie können sich an die Sarkoidose-Selbsthilfe wenden, wo Sie bestimmt gute Tipps erhalten werden. https://www.sarkoidose.de/. Ich wünsche alles Gute. | Ja, es gibt 11 Sarkoidose Spezialisten in Niedersachsen. Sie sind an 2 Krankenhäusern tätig, nämlich in Hannover und Braunschweig. Außerdem gibt es 5 Sarkoidose Spezialisten in der niederländischen Grenzregion rund um die Großstädte Osnabrück, Oldenburg und Diebsen. Sie sind alle an Krankenhäusern in der Region Niedersachsen tätig. Sie können auch telemedizinisch kontaktiert werden. Sie haben insgesamt 16 Kontaktpersonen, das sind 5 Kontaktpersonen je Spezialist. | Es gibt mehrere Spezialisten für Sarkoidose im Raum Niedersachsen/Hannover. Ein renommierter Ansprechpartner ist die Medizinische Hochschule Hannover (MHH), insbesondere die Abteilung für Pneumologie. Dort arbeiten Experten, die auf Lungenerkrankungen und Sarkoidose spezialisiert sind. Es wäre sinnvoll, sich direkt an die MHH zu wenden, um einen Termin zu vereinbaren und genauere Informationen zu erhalten. |
| Hallo,  Bei mir wurde letztes Jahr im Juni Sarkoidose diagnostiziert. Ich hatte ca. 6 Monate lang einen leicht schmerzhaften Knoten am linken Schienbein, der seine Position von anfänglich der Mitte bis zum Schluss am Gelenkansatz verändert hat. Ein Röntgen der Lunge hat keine Auffälligkeiten ergeben. Nun habe ich seit 1 Woche wieder einen Knoten in der Mitte des linken Schienbeins. Ist ein erneutes Röntgen der Lunge ratsam oder sollte hiermit noch gewartet werden?  Vielen Dank bereits im Vorraus. | Solche Veränderungen stehen zwar häufig aber nicht ausschließlich mit einer bestimmten Erkrankung im Zusammenhang. Immer ist eine medizinische Abklärung sinnvoll - möglicherweise auch eine weitergehende Untersuchung (Röntgen oder CT der Lunge) usw. Aus der Ferne kann man aber weder die Entscheidung für oder gegen eine Röntgenaufnahme treffen. Beste Grüße. | Wenn sich der Schmerz nicht rasch verbessert, sollte man sich dem nächsten Arzt vorstellen und weitere Untersuchungen verlangen. Da der Knoten im Gelenkansatz liegt, ist es möglich, dass er nicht mit Sarkoidose assoziiert ist. Eine Untersuchung könnte jedoch zu weiteren Hinweisen führen. Es könnte sich gut eignen, eine Biopsie vorzunehmen, wenn dies nicht riskant wäre. | Hallo, bei Sarkoidose können Knoten an verschiedenen Körperstellen auftreten. Da Ihr letzter Röntgenbefund unauffällig war und Sie nun erneut einen Knoten bemerken, wäre es sinnvoll, dies ärztlich abklären zu lassen. Ein erneutes Röntgen der Lunge könnte helfen, um sicherzustellen, dass keine neuen Auffälligkeiten aufgetreten sind. Es ist wichtig, Ihre Symptome regelmäßig zu überwachen und Rücksprache mit Ihrem behandelnden Arzt zu halten, um eine angemessene Behandlung zu gewährleisten. |
| Mein Freund hat vor zweieinhalb Jahren die Diagnose Sarkoidose erhalten. Ich beobachte zu den Zeiten der vermuteten Schübe, denn zum Arzt gehen vermeidet er, extreme Stimmungsschwankungen, bzw. vor allem ein enormes Aggressionspotential. Ich erkenne ihn dann kaum wieder.  Ist es möglich, dass diese Stimmungsschwankungen mit der Erkrankung zusammenhängen? Und vielleicht haben Sie eine Adresse für mich, an die ich mich für Unterstützung und Beratung wenden kann? | Stimmungsschwankungen, Konzentrationsstörungen, reaktiv-depressive Zustände bis zum aggressiven Verhalten sind bei bis 70% der Patienten mit Sarkoidose beschrieben (Voortman M, et al. The Burden of Sarcoidosis Symptoms from a Patient Perspective. Lung. 2019). Eine neurologische Beteiligung muss unbedingt ausgeschlossen werden. Verhaltenstherapie sowie Verschreibung von spezifischen Medikamenten kann sehr hilfreich sein, um solche Krankheitsmanifestationen zu beherrschen. Bitte wenden Sie sich für die Beratung und weitere Behandlung an ein Sarkoidose Referenzzentrum. Mit den besten Wünschen | Ich habe nicht die Möglichkeit, mich mit Ihnen direkt zu vernetzen, aber ich kann Ihnen bei Bedarf Kontaktdaten von Experten zur Verfügung stellen. Die Sarkoidose ist eine seltene Autoimmunerkrankung, welche verschiedene Organe befallen kann. Der Zusammenhang zwischen der Sarkoidose und dem Aggressionsverhalten ist nicht bekannt. Es kann aber eine psychische Komorbidität wie Depression oder Angstzustände vorliegen. Wenn die Diagnose gesichert ist, müsste der Psychiater die auslösenden Faktoren und die Behandlung der Stimmungsschwankungen feststellen. Es kann eine Kombination aus psychologischer Behandlung und Medikamentenversuch, wie beispielsweise Antidepressiva und Benzodiazepine, erforderlich sein. | Ja, es ist möglich, dass die Stimmungsschwankungen und das Aggressionspotential mit der Sarkoidose zusammenhängen, da entzündliche Erkrankungen das zentrale Nervensystem beeinflussen können und solche Symptome verursachen. Es wäre wichtig, dass Ihr Freund ärztliche Hilfe in Anspruch nimmt, um diese Symptome besser zu managen. Für Unterstützung und Beratung können Sie sich an die Deutsche Sarkoidose-Vereinigung e.V. wenden. Sie bietet Informationen, Beratungen und den Austausch mit anderen Betroffenen. |
| Guten Tag.  Bei mir wurde vor 3 Jahren Sarkoidose 2 der Lunge diagnostiziert, bin regelmäßig zur Kontrolle bei meinem Lungenarzt und nehme seither Prednisolon. Im Moment 1,5 mg täglich. Nun befinde mich seit ein paar Monaten im Ausland und habe vor 3 Wochen leichtes Fieber bekommen. Das Fieber ist seit ein paar Tagen konstant hoch (um die 39 Grad). Ich war bereits im Krankenhaus (in Kolumbien) und dort wurde nach einer Blutentnahme, röntgen der Lunge und Urinuntersuchung nichts festgestellt. Die Werte sind normal. Nun versuche ich herauszufinden, ob das erhöhte Fieber mit der Sarkoidose in Verbindung stehen könnte. Ich habe keine weiteren Symptome, außer leichten, gelegentlichem Husten. Mein Lungenarzt in Deutschland ist im Urlaub und auch dessen Vertretungen sind nicht erreichbar (meine Mutter hat von Deutschland aus dort angerufen).  Vielleicht können Sie mir weiterhelfen und beantworten, ob es möglich ist, dass das Fieber mit der Sarkoidose zu tun haben könnte.  Vielen lieben Dank und herzliche Grüße. | Tatsächlich kann erhöhte Temperatur auch einmal alleiniges Zeichen für eine Aktivität der Sarkoidose sein, 39° ist dafür allerdings eher ungewöhnlich hoch. Wenn aber insbesondere Medikamenten-bedingtes Fieber und eine Infektion ausgeschlossen sind, muss man daran denken. Ohne zu wissen, was im Einzelnen schon untersucht wurde, kann man von hier aus natürlich nicht sagen, ob man die Sarkoidose wirklich als wahrscheinlich annehmen kann. Ich hoffe, es geht Ihnen weiter gut und Sie konnten inzwischen auch Ihren betreuenden Arzt erreichen. Mit den besten Wünschen. | Ja, es ist möglich. Sarkoidose ist eine seltene Erkrankung. Es gibt eine gute Therapie. Ein hohes Fieber mit einem Normalbefund in einer Blutuntersuchung ist nicht ganz ungewöhnlich. Wenn Sie die Sarkoidose im Krankenhaus nicht erinnern konnten, ist die Ursache vielleicht eine Reaktion auf die Medikation oder eine andere Infektion. Die Diagnose kann aber nur über eine Biopsie gesichert werden. | Guten Tag, es ist möglich, dass das Fieber mit Ihrer Sarkoidose in Verbindung steht, insbesondere da diese eine systemische Erkrankung ist, die verschiedene Organe betreffen kann. Allerdings könnte das anhaltend hohe Fieber auch auf eine andere Infektion oder Entzündung hinweisen, die möglicherweise nicht sofort durch die Untersuchungen im Krankenhaus erkennbar war. Da Fieber ein ernstzunehmendes Symptom ist, empfehle ich dringend, erneut ärztliche Hilfe aufzusuchen, um weitere Ursachen auszuschließen und sicherzustellen, dass keine behandlungsbedürftige Infektion vorliegt. Halten Sie Ihre regelmäßigen Medikamente ein und überwachen Sie Ihre Symptome genau. Gute Besserung und herzliche Grüße. |
| According to various studies, the risk of osteoporosis appears to be increased in patients with bronchiectasis, especially if there is a chronic infection and possibly even more if oxygen decreases during exercise.  What are your recommendations for elderly patients (over 60, male or female) with bronchiectasis and pseudomonas infection (dependent or not on oxygen) regarding measurement of bone density and osteoporosis prophylaxis? | Thank you. Patients with bronchiectasis are at increased risk of osteoporosis. Measurement of bone density is recommended for patients aged >65 years with bronchiectasis, or those with risk factors such as underweight or prior steroid therapy. Patients with reduced bone mineral density or who have risk factors for fracture including the use of long term corticosteroids or frequent courses of corticosteroids should receive prophylaxis. | In the case of patients with bronchiectasis, it is recommended to measure the bone density of patients over the age of 60, regardless of gender, due to the increased risk of osteoporosis. Osteoporosis prophylaxis should be considered in patients with a high risk of osteoporosis. This may include the use of medications such as calcium and vitamin D supplements and bisphosphonates, as well as the avoidance of activities that increase the risk of falls. It is also important for patients with bronchiectasis to have regular follow-ups with their doctor to monitor any changes in their condition that may affect their bone health. | For elderly patients with bronchiectasis and pseudomonas infection, regular bone density measurements, such as a DEXA scan, are essential to monitor for osteoporosis, particularly if they have chronic infections or require oxygen. Prophylactic measures should include adequate calcium and vitamin D intake, weight-bearing exercises, and potentially bisphosphonates or other osteoporosis medications as advised by their healthcare provider. Close monitoring and management of their respiratory condition can also indirectly help in reducing the risk of osteoporosis. It is crucial to have a personalized treatment plan developed in consultation with both pulmonology and endocrinology specialists. |
| How to manage a patient with chronic left ventricular failure (EF 40%) due to coronary artery disease who suffered intermediate risk PE and now reports significantly decreased exercise capacity? | The question does not really relate to a rare disease, but it may correspond to CTEPH at the end of a diagnostic process... Last, the clinical information is too limited to provide an accurate reply. Under these circumstances the answer is as follows: - Decreased exercise capacity in such a patient is more likely to correspond to a common cause outside the spectrum of diseases typically managed in specialized networks (chronic left heart disease, deconditioning...) or less common causes (CTEPH) that fall within specialized care missions. Both are non mutually exclusive... - Cohort studies have revealed that persisting or deteriorating dyspnoea and poor physical performance are frequently present 6 months to 3 years after an acute PE episode. The proportion of patients claiming that their health status is worse at 6-month follow-up than it was at the time of PE diagnosis varies widely, ranging between 20% and 75%. The following baseline parameters and findings could be identified as predictors of exertional dyspnoea at long-term follow-up after PE: Advanced age, cardiac or pulmonary comorbidity, as well as higher body mass index and a smoking history - In cases of decreased exercise capacity after acute PE, this may correspond to chronic thromboembolic disease with or without pulmonary hypertension (CTEPH). Is there any clinical evidence of right heart failure and/or PH? The best screening tool for CTEPH is ventilation/perfusion lung scintigraphy. Doppler echocardiography will allow evolution of the right and left side of the heart and estimate sPAP. If V/Q lung scan shows mismatched perfusion defects, a computed tomography pulmonary angiography will allow an assessment of possible CTEPH. Right heart catheterization and pulmonary angiography performed in an expert center with a multidisciplinary team will allow complete hemodynamic assessment and imaging (and discuss management). Best wishes. | The management of chronic left ventricular failure due to coronary artery disease is a multidisciplinary approach that involves a team of specialists, including cardiologists, heart failure specialists, and other healthcare providers. The main goals of management are to reduce heart failure-related symptoms, improve quality of life, and reduce hospitalizations. | Managing a patient with chronic left ventricular failure (EF 40%) and a history of intermediate-risk pulmonary embolism involves optimizing heart failure treatment, ensuring anticoagulation to prevent further embolic events, and addressing decreased exercise capacity through a tailored cardiac rehabilitation program. Medications like ACE inhibitors, beta-blockers, and diuretics should be optimized for heart failure, while anticoagulation therapy should be continued as per guidelines for PE. Regular follow-up with a cardiologist is essential to monitor and adjust treatment, and supervised exercise programs can help improve functional capacity and quality of life. |
| It seems that children are less often infected by the new Coronavirus and especially that the course of the disease is on average less severe in children than in adults.  Most children are said to be infected by adults, e.g. their parents.  Now gradually the schools will open again and many fathers or mothers with PCD are afraid that their children might bring the infection home from school.  However there are experts that say this is very unlikely as until now there are no or hardly any cases known where children did infect their parents.  Please let us know what you as expert think about the risk for parents to be infected by their children. | The raised question is currently a research focus and not yet answered. Indeed, infected children show on average a more benign course of COVID-19. Children younger than 10 years of age are supposed to become less likely infected and in addition, once infected, might less likely infect adults, because their cough is much weaker compared to the coughing of adults. However, other studies have demonstrated that young children probably can infect other individuals. Due to living within the same household, infected children might therefore infect their parents. | The risk for parents of children who attend school to contract COVID-19 is believed to be low, especially if proper safety measures and guidelines are followed. However, it is important to note that the current situation with the COVID-19 pandemic is constantly evolving, and it is difficult to predict how the risk may change. Therefore, parents should consider the potential risks and benefits of having their children attend school, and should follow the guidelines and recommendations of health authorities. | It's true that children generally have milder cases of COVID-19 and are less often the source of infection compared to adults. However, the risk is not zero, and children can still transmit the virus, particularly in households. With schools reopening, it's essential to follow preventive measures like hand hygiene, wearing masks, and physical distancing as much as possible. For parents with underlying conditions such as PCD, maintaining these precautions and staying informed about the local infection rates is crucial. Consulting with a healthcare provider for personalized advice can also help in managing the specific risks for your family. |
| Following to several studies there seems to be an elevated risk of osteoporosis in patients with Non-CF-bronchiectasis, especially if there is a chronic infection and also if they are desaturisers during exercise.  What are the consequences of this for elderly PCD-patients concerning prophylaxis of osteoporosis and need of measurement of bone mineral densitiy? | This is a good question. However, so far we do not have any evidence based answer to this question because it has never been studied. We would need a study to clarify this issue. Best wishes. | Patients with noncystic fibrosis bronchiectasis (nCFBE) have a higher risk of osteoporosis, especially in the setting of chronic infection and desaturation during exercise. 24 As PCD patients are at an increased risk of osteoporosis, 25 it is important to screen for this condition in those with a history of chronic infection and/or desaturation during exercise. The optimal approach to screening and management of osteoporosis in PCD patients has not yet been determined, but recommendations from the National Institute for Health and Care Excellence suggest screening with dual-energy x-ray absorptiometry (DEXA) for women age 50 years or older and dual-energy x-ray absorptiometry (DEXA) for men age 70 years or older. 26 Patients with PCD and a low bone mineral density (BMD) should be monitored regularly, with a yearly repeat DEXA scan recommended. | For elderly patients with primary ciliary dyskinesia (PCD), who often suffer from chronic infections and may experience desaturation during exercise, the elevated risk of osteoporosis warrants proactive measures. Prophylaxis should include adequate calcium and vitamin D intake, regular weight-bearing exercise, and possibly medications to strengthen bones. It's also important to regularly measure bone mineral density to monitor bone health and adjust treatment plans as necessary to prevent fractures and other complications associated with osteoporosis. |
| Are you aware of any closed-loop systems that titrate oxygen delivery based on O2 sats? I have friends who have diabetes and they have insulin pumps that deliver just enough insulin to keep them in the safe zone at all times. Shouldn't we have that type of machine for oxygen delivery? | To our knowledge, there is no closed-loop system that titrates oxygen delivery based on O2 sats for mobile oxygen supply systems available. Such a system is available to test the need for oxygen within the pulmonary function test lab, but not yet implemented in oxygen care systems. Best regards | The function of oxygen-delivery devices and insulin pumps are fundamentally different. Insulin pumps infuse insulin in order to manage blood glucose levels. Oxygen-delivery devices deliver oxygen in order to deliver oxygen. This difference in function necessitates the use of different types of sensors and control algorithms to ensure patient safety. | Yes, closed-loop systems for oxygen delivery do exist and are being developed to automatically titrate oxygen based on a patient's oxygen saturation levels, similar to insulin pumps for diabetes. These systems use continuous monitoring of O2 sats and adjust oxygen flow to maintain optimal levels, improving patient safety and comfort. Such technology is particularly useful for patients with chronic respiratory conditions, aiming to reduce the risk of hypoxemia and hyperoxemia. However, these systems are still being refined and may not be widely available yet. |
| Kann aus Sarkoidose Lymphom entstehen ,bzw wie häufig CT Kontrolle bei Sarkoidose. Betroffen sind Lymphsystem, Milz Lunge Niere? | Über die Frage, ob ein erhöhtes Risiko für die Entstehung eines Lymphoms bei bekannter Sarkoidose besteht, wird seit Jahren debattiert. Eine regelmäßige CT Kontrolle als Screening für Lymphom ist deswegen nicht gerechtfertigt. | CT Kontrolle bei Sarkoidose kann helfen Lymphom zu identifizieren, jedoch ist es nicht obligat. Bei häufigem Auftreten von Lymphomen bei Sarkoidose-Patienten und schlechter Prognose ist eine Kontrolle empfohlen. | Sarkoidose und Lymphome sind unterschiedliche Erkrankungen, und obwohl selten, kann Sarkoidose in seltenen Fällen mit der Entwicklung von Lymphomen assoziiert sein. Die Häufigkeit von CT-Kontrollen bei Sarkoidose hängt vom individuellen Krankheitsverlauf und den betroffenen Organen ab, aber in der Regel werden Kontrollen alle 6-12 Monate empfohlen, um die Krankheitsaktivität zu überwachen. Sprechen Sie mit Ihrem behandelnden Arzt über einen spezifischen Überwachungsplan, der auf Ihre individuellen Bedürfnisse zugeschnitten ist. |
| Guten Tag ,  ich habe zur Zeit ein aktive und teilweise inaktive Sakoidose , heißt wir behandeln mit Cortison so lange bis die Werte OK sind ,schleichen das Cortison aus und kontrollieren ob die Trigger in Normbereich bleiben , sind jetzt 3 Jahre dabei aber leider kommt die Sakoidose immer nach kurzer Zeit wieder ,nachdem ich das Cortison abgesetzt habe,  Wenn ich das Cortison absetzte verliere ich immer Geschmack und Geruch ,kann das von der Sakoidose kommen????? ( Nebenrkrankunge ist Asthma ) | Wie lange sind denn jeweils Ihre Behandlungsphasen? Haben Sie schon einmal eine andere Therapie als Cortison versucht (Methotrexat oder Azathioprin)? Man sagt ja, die Sarkoidosetherapie braucht eine ruhige Hand und manchmal ist eine längerfristige sehr niedrig dosierte Therapie besser als die ständigen Reaktivierungen beim Rückzug der Cortisondosis. Aber das kann man aus der Ferne natürlich nicht gut beurteilen.  Der Verlust des Geruchssinnes ist nicht erkrankungsspezifisch, er zeigt eher eine gewisse entzündliche Aktivität im Bereich der Nasenschleimhäute an, weshalb es ja wohl auch bei Corona vorkommt, aber eben auch bei Asthma. Bei Sarkoidose kann ja nun wirklich alles vorkommen und deshalb eben auch das. Wesentlich öfter ist es mir bei Patienten mit Asthma begegnet, aber das ist eben auch ein häufigeres Krankheitsbild. Beste Grüße. | 3 Jahre Sarkoidose und Kortikosteroidtherapie sind eine lange Zeit, und es ist verständlich, dass Sie die Kortikosteroidtherapie beenden möchten. In Fällen, in denen die Kortikosteroidtherapie abgesetzt werden sollte, wird häufig zunächst versucht, die Dosis zu reduzieren. Ein „Taper“ ist eine langsame Verringerung der Dosis über einen bestimmten Zeitraum hinweg. Das „Taper“ sollte unter regelmäßiger Kontrolle Ihrer Blutwerte erfolgen. Ein „Taper“ muss sorgfältig durchgeführt werden, und das Ziel ist es, die Kortikosteroidtherapie zu beenden, ohne einen Sarkoidose-Schub auszulösen. Es scheint, dass die Sarkoidose in Ihrem Fall sehr empfindlich auf den Entzug von Kortikosteroiden reagiert, was nicht ungewöhnlich ist. Es wäre sehr wichtig, darauf zu achten, dass die gesamte Kortikosteroidtherapie ausschleichend und nicht abrupt abgesetzt wird. Wenn die Kortikosteroidtherapie zu abrupt abgesetzt wird, kann die Sarkoidose zurückkehren. Sie können versuchen, die Kortikosteroidtherapie abzusetzen, und wenn die Sarkoidose wieder auftritt, können Sie erneut einen „Taper“ versuchen. | Guten Tag, es ist möglich, dass der Verlust von Geschmack und Geruch sowohl mit der Sarkoidose als auch mit der Cortisonbehandlung zusammenhängt. Sarkoidose kann Entzündungen in verschiedenen Körperteilen verursachen, einschließlich der Nerven, die für Geruch und Geschmack verantwortlich sind. Cortison kann ebenfalls Nebenwirkungen haben, die diese Sinne beeinträchtigen. Es wäre ratsam, dies mit Ihrem behandelnden Arzt zu besprechen, um eine genaue Ursache festzustellen und alternative Behandlungsstrategien zu erwägen. |
| Gibt es Empfehlung hinsichtlich Vorsichtsmaßnahmen bezüglich Corona? Ich habe einen 13 jährigen Sohn mit PCD und wir sind unsicher welche Vorsichtsmaßnahmen angebracht sind.  Vielen Dank | Wir können leider auch nicht viel mehr sagen als das, was die öffentlichen Stellen dazu sagen: Abstand halten und auf Hygiene achten. Das ist natürlich Eulen nach Athen tragen, wenn man das Eltern von einem Kind mit PCD sagt, aber mehr gibt es wirklich nicht.  Ich wünsche Ihnen Alles Gute. Beste Grüße. | Wenn ein Kind an der PCD leidet, sollte man sich davor halten, an der PCD zu erkranken. Daher ist es wichtig, dass Kinder mit PCD immer nach den aktuellen Vorsichtsmaßnahmen leben, um das Risiko für schwerwiegende oder tödliche Krankheitsverläufe zu reduzieren. | Bei einem 13-jährigen Sohn mit Primärer Ciliärer Dyskinesie (PCD) sind besondere Vorsichtsmaßnahmen gegen Corona wichtig. Achten Sie auf gründliches und regelmäßiges Händewaschen, das Tragen von Masken in öffentlichen Innenräumen, die Vermeidung von Menschenmengen und die Einhaltung sozialer Distanz. Es ist ebenfalls ratsam, sicherzustellen, dass er vollständig geimpft ist, falls er dafür in Frage kommt, und regelmäßige medizinische Kontrollen wahrzunehmen. Sprechen Sie auch mit seinem behandelnden Arzt, um individuell zugeschnittene Empfehlungen zu erhalten. |
| Hi,  My uncle went to the hospital 3 weeks ago, because he couldn't breath properly. He was immediately hospitalized, after 6 days the doctors did a lung scan and determined that he had pulmunary fibrosis. The same day he was transfered to another hospital and he was intubated. From that moment, from 2 weeks ago, the doctors tried to understand the cause, they told me that they cannot do a biopsy, that the lungs transplant is impossible to do because of his age, They told me that they did another body scan 4 days ago and the result is that during these two weeks this illness completely destroied both his lungs and now only half of the left lung is without fibrosis. My question is the following: is it possible that this illness goes so fast that in two weeks both lungs are completely destroied? do you know if there is any experimental therapy that can we try as last hope? thank you | Without having the possibility to evaluate the CTs of the lungs and without knowledge of further clinical information it is almost impossible to give a good advice. With some probability an acute exacerbation of an underlying pulmonary fibrosis may have occurred or an ARDS may be present. Both would fit with the clinical development you describe. Therapy should be according to current guidelines. I would like to add, that your narrative sounds like your uncle has been transferred to a specialist centre and we know quite a few of these centers in Italy, which means that such centers have access to all treatment options available in Europe. I wish you and especially your uncle all the best. | I'm sorry to hear about your uncle. It sounds like he is suffering from a very serious condition. The destruction of both lungs in just two weeks is indeed very fast. Is it possible that the lung fibrosis had been present for a longer time, but remained undetected until the X-ray or CT scan? | I'm very sorry to hear about your uncle's condition. Pulmonary fibrosis can sometimes progress rapidly, especially in cases of acute exacerbation, where inflammation and fibrosis accelerate quickly, leading to severe lung damage in a short time. While it is indeed possible for the disease to progress so fast, it is rare and typically indicates a severe underlying cause or an aggressive form of the disease. Experimental therapies, such as antifibrotic drugs, stem cell therapy, or participation in clinical trials, might be options, but they are typically more effective in slowing progression rather than reversing damage. It would be best to discuss these possibilities with his healthcare team to understand the most suitable and feasible options for his specific situation. |
| Dear Colleagues, Could you please kindly suggest what bone protection would be suitable for an older female patient with pulmonary sarcoidosis who is on long term steroids . She has confirmed osteoporosis and fragility fractures, but could not tolerate oral bisphosphonates ( dyspepsia). Would Prolia be a suitable alternative? Or is Zoledronic acid a better choice? I have not been able to find guidelines on bone protection for patients with sarcoidosis. Many thanks in advance, | Yes, Prolia is an excellent alternative to bisphosphonates and there are no contraindications in sarcoidosis. At yet, there are no official guidelines on bone protection for patients with sarcoidosis. Best wishes | Good afternoon! Regarding your question about bone protection for patients with pulmonary sarcoidosis taking long-term steroids, the first-line option is a bisphosphonate such as alendronate or risedronate. If the patient is unable to take these due to gastrointestinal intolerance, you may consider Denosumab, a monoclonal antibody against RANKL, as the second-line option. However, it is worth noting that there is limited data on Denosumab in patients with sarcoidosis. In terms of the risk of skeletal events, both bisphosphonates and Denosumab have shown to be effective in preventing fractures and decreasing vertebral deformities. It is important to note that Denosumab is not recommended for patients at high risk of infection (such as those with pulmonary sarcoidosis) due to the risk of increased infection. If the patient is at high risk of infection, alendronate is recommended. | For an older female patient with pulmonary sarcoidosis on long-term steroids, who has confirmed osteoporosis and fragility fractures but cannot tolerate oral bisphosphonates, Prolia (denosumab) could be a suitable alternative. Prolia is often used in patients with similar profiles due to its efficacy and subcutaneous administration, which can be better tolerated. Zoledronic acid is also an effective option, given intravenously, and might be considered if there are no contraindications and the patient can tolerate it. Consulting with a specialist in endocrinology or rheumatology may provide further tailored guidance. |
| What would be your recommendation for treatment od ACIF (airway-centered interstitial fibrosis)? | You are certainly aware that the term: airway-centered interstitial fibrosis is a hitopathological finding and this is found in a number of clinical conditions like hypersensitivity pneumonitis or gastroesophageal reflux syndrome. The heterogeneity makes it impossible to give a clear recommendation for treatment. If there is a lot of inflammation (pneumonitis) steroids would be a treatment option, but this will certainly depend on individual findings. Best regards. | The treatment of patients with airway-centered interstitial fibrosis (ACIF) often includes a combination of interventions aimed at improving lung function and preventing or managing respiratory-related symptoms. Some possible treatment options that may be considered for ACIF include bronchodilators, glucocorticoids, antioxidants, and oxygen therapy. Additionally, lung-volume reduction surgery and lung transplant may be considered in patients with severe ACIF who do not respond to other treatments. The choice of treatment approach will likely depend on the specific severity and extent of the patient's lung involvement, as well as their overall health status. | Airway-centered interstitial fibrosis (ACIF) treatment typically focuses on managing symptoms and preventing further lung damage. My recommendation would be to start with corticosteroids to reduce inflammation, and in some cases, immunosuppressive drugs may be considered. It's essential to avoid any environmental exposures that could worsen the condition, and pulmonary rehabilitation can help improve lung function and quality of life. Consulting with a pulmonologist for a personalized treatment plan is crucial. |
| Dislike taking prednisone for chronic hypersensitivity premonititis. What alternatives are there? | You are certainly aware that there are numerous causes of Hypersensitivity pneumonitis (HP) and they are not easily diagnosed. We usually take the steroid treatment responsiveness as an important diagnostic finding. This is one reason why we use corticosteroids like prednisolone for treatment. There are other immunosuppressant drugs like Azathioprine, Methotrexate, etc. but the benefit-risk-ratio is very good for Corticosteroids. And, we have lots of experience steroids from other diseases and only a limited number of clinical trials with those other drugs. Some reluctance to the use of glucocorticoids is easy to understand, but there are patients where we do not see a good alternative. Best regards | Prednisone is a common treatment for autoimmune conditions, including chronic hypersensitivity premonititis. However, if you dislike taking prednisone for the condition, there are several alternative treatments available, such as other corticosteroids, corticosteroid-sparing agents, or immunomodulatory agents. It is important to consult with a healthcare professional, such as a rheumatologist or dermatologist, for personalized recommendations for the treatment of your condition. They can help you manage your symptoms and choose the best treatment options for your situation. | For chronic hypersensitivity pneumonitis, alternatives to prednisone may include other immunosuppressive medications like azathioprine or mycophenolate mofetil. In some cases, inhaled corticosteroids or antifibrotic agents like pirfenidone or nintedanib might be considered. It’s important to discuss these options with your doctor to tailor the treatment to your specific condition and tolerance. |
| -Einflüsse der Ernährung auf den Verlauf einer Sarkoidose?  -Gibt es Einflussfaktoren einer Diabetes Typ II auf den Verlauf einer Sarkoidose?  - Überlagerung einer Sarkoidose (Diagnose 1/2 Jahr durch Zufallsbefund) mit einem seit ca. 20 Jahren festgestellten Asthma, gibt es hierzu Literteratur / Studien/Publikationen? | Zu Ihren Fragen gibt es keine durch Evidenz hoher Qualität gesicherten Erkenntnisse, die Eingang in die Leitlinien gefunden hätten. Diabetes ist nicht selten in der betroffenen Altersgruppe, auch ist natürlich das Auftreten eines Diabetes unter der Cortisontherapie zu berücksichtigen. Auch ein gehäuftes Auftreten von Asthma und Sarkoidose ist nicht gesichert, obwohl es natürlich ein Zusammentreffen beider Erkrankungen geben kann. Beste Grüße | Es liegen aktuell keine Daten zur Rolle der Ernährung bei der Sarkoidose vor. Für eine Diabetes Typ-II-Therapie sind, bei der Therapie von Sarkoidose eine Ernährungsintervention und eine Glykämie-Kontrolle empfohlen. Das Vorliegen von Asthma bei der Sarkoidose wurde bisher nur in wenigen Fallberichten und einer kleinen Studie beschrieben . Die Datenlage ist daher zu gering, um eine konkrete Empfehlung zu geben. | Die Ernährung kann bei Sarkoidose den allgemeinen Gesundheitszustand beeinflussen, jedoch gibt es keine spezifischen Lebensmittel, die den Verlauf der Krankheit direkt verändern. Diabetes Typ II kann den Verlauf der Sarkoidose beeinflussen, indem er das Risiko für Infektionen erhöht und die Entzündungsreaktion im Körper verstärken kann. Die gleichzeitige Diagnose von Sarkoidose und Asthma ist in der medizinischen Literatur beschrieben, und es gibt Studien, die sich mit der Koexistenz und den möglichen Wechselwirkungen dieser beiden Erkrankungen beschäftigen. Es wäre hilfreich, mit Ihrem Arzt über spezifische Studien und Behandlungsmöglichkeiten zu sprechen, die auf Ihre individuelle Situation zugeschnitten sind. |
| Hautärztin meint ich habe Haut Sarkoidose. Habe Hautveränderungen am Bein, kleine Knochenveränderungen am Bein, erhebliche Leistenschmerzen-Gelenkschmerzen, tlw. Kurzatmigkeit, keine Leistungsfähigkeit mehr. War zum Lungenröntgen, Ergebnis gibt es noch, weiterer Vorstellungstermin im April. Ist mir leider nicht exakt bzw. zu oberflächlich. Bei welchem Arzt könnte ich mich vorstellen? | Zur Klärung der Frage, ob eine systemische Beteiligung an einer Sarkoidose vorliegt, kann am besten der Pneumologe (Lungenfacharzt) beitragen. Einen guten Einstieg und viele Informationen zu dem Thema finden Sie bei der Deutschen Sarkoidosevereinigung. Beste Grüße. | Ich würde Ihnen empfehlen, für Ihre Hautsymptome einen Facharzt für Dermatologie aufzusuchen. Für Ihre Lungensymptome sollten Sie einen Lungenfacharzt aufsuchen. | Bei Hautsarkoidose und den von Ihnen beschriebenen Symptomen sollten Sie sich an einen Lungenfacharzt (Pulmologen) wenden, da Sarkoidose oft die Lungen betrifft. Zudem könnte ein Rheumatologe für Ihre Gelenkschmerzen und Leistenschmerzen hilfreich sein, und ein Internist oder Allgemeinmediziner kann eine umfassende Koordination Ihrer Behandlung übernehmen. Es ist wichtig, dass Sie Ihre Symptome genau schildern und alle relevanten Untersuchungsergebnisse mitbringen. |
| Bei Osteoporose ist Vit D entsprechend dem Spiegel zu supplementieren, bei Sarkoidose soll kein Vit D zugeführt werden.  Spiegelbestimmung des aktiven Vit D ist auch für entsprechende Fachärzte schwierig. Die Interpretation des Spiegels konnte dann vom Facharzt auch nicht aussagekräftig vorgenommen werden. | Bei Patienten mit Sarkoidose sollte man beide Vitamin D-Werte (aktiv UND inaktiv) im Serum messen lassen. Nur wenn beide Werte niedrig sind, kann eine Substitutionstherapie mit DEKRISTOL eingeleitet werden. Dass die Spiegelbestimmung schwierig sei, kann ich nicht bestätigen. Bei Sarkoidose muss keine Calcium-Zufuhr zusätzlich zu Vitamin D erfolgen, das ist eher nachteilig, aber Vitamin D allein (wenn beide Werte im Serum niedrig sind, siehe oben) ist kein Problem. | In diesem Fall wäre eine 6‑wöchige Kortisontherapie mit einer Gesammdosis von 20 mg/Tag die beste Behandlungsmethode. Eine 12-wöchige Behandlung wäre nicht indiziert, da kein Vorteil durch eine längere Therapiezeit erreicht werden würde. | Die Therapie der Larynxsarkoidose mit Glucocorticoiden kann oft langwierig sein, insbesondere bei wiederkehrenden Symptomen. Es ist wichtig, die Prednison-Dosis so gering wie möglich zu halten, um Nebenwirkungen zu minimieren. Da Sie eine Schwangerschaft planen, sollten Alternativen wie Methotrexat oder Azathioprin in Erwägung gezogen werden, aber diese müssen vor einer Schwangerschaft abgesetzt werden. Sprechen Sie ausführlich mit Ihrem behandelnden Arzt, um einen individuellen Plan zu erstellen, der sowohl Ihre Gesundheit als auch Ihre Familienplanung berücksichtigt. |
| Patient männlich, 60J.  Sarkoidose seit 15J bekannt, aber laut Röntgen und LuFu in Remission.  Patient berichtet über morgentliche Atemgemgeräusche (vorzugsweise beim Einatmen), die an zerplatzenden Badeschaum erinnern und er demonstriert auch eine Aufioaufnahme am Handy. Der Patient hat sichtlich trockene Gesichtshaut, trockene Schleimhaut im HNO-.Bereich ist bekannt, Augentropfen werden verwendet. Der Patient meint, die Intensität des sauren Geschmacks von Schleim würde meist mit langdauernden Müdigkeitsphasen korrelieren, die morgentlichen Atemgeräusche würden mit noch mehr Müdigkeit korrelieren.  Ist eine Abklärung notwendig und falls ja, was ist die empfohlene Vorgangsweise (Differentialdiagnosen?)? | Der Patient sollte am besten eine komplette Lungenfunktionsdiagnostik bekommen. Eine obstruktive Ventilationsstörung kann sich nach Remission der Sarkoidose entwickeln und persistent sein. Eine adäquate Therapie mit Bronchodilatoren sollte rechtzeitig eingeleitet werden. Eine Abklärung wegen Sjögren Syndrom beim Rheumatologe sollte ebenfalls erfolgen. Beste Grüße. | In der Regel können wir annehmen, dass Lymphopenie die Aktivität des Sarkoidoses im Blut reflektiert. Es ist bekannt, dass Patienten mit Lymphopenie bei Sarkoidose ein erhöhtes Risiko für eine schwere Krankheitsverlaufsform haben . Es ist auch bekannt, dass Patienten mit Sarkoidose und COVID-19 eine erhöhte Mortalität haben . Daher könnte der Mangel an Lymphozyten im Blut ein Indikator dafür sein, dass COVID-19 mit Sarkoidose kombiniert ist und ein schwerer Verlauf nimmt. Es ist aktuell noch unbekannt, ob Sarkoidose ein Risikofaktor für den schweren Krankheitsverlauf bei COVID-19 ist und ob ein Therapieansatz auf den Sarkoidose-Abschnitt wirkt oder nicht . | Die Therapie der Neurosarkoidose mit Azathioprin und Cortison ist ein üblicher Ansatz, bei dem Azathioprin als Immunsuppressivum wirkt und Cortison Entzündungen reduziert. Eine schrittweise Reduktion der Medikamente ist möglich, sollte aber unter engmaschiger ärztlicher Überwachung erfolgen. Der IL-2-Rezeptor kann als Marker zur Überwachung der Krankheitsaktivität hilfreich sein, jedoch sind auch andere Laborwerte wie ACE, sIL-2R und Lysozym relevant. Für spezialisierte Zentren und Ansprechpartner in Sachsen empfehle ich, Kontakt mit der Universitätsklinik Leipzig oder Dresden aufzunehmen, da diese oft spezialisierte Abteilungen für seltene Krankheiten haben. |
| Hallo,  bin 55, weiblich, Nichtraucherin. Bei mir ist es fast sicher (warte auf den entscheidenden Termin zur endgültigen Diagnosestellung beim Pneumologen – am 1.7.!), dass ich an Sarkoidose der Lunge, Lymphknoten und wirklich massiver Knochenbeteiligung (vor allem Becken aber auch LWS und Femur) festgestellt worden. Maligne Erkrankungen wurden ausgeschlossen. Folgende Diagnostik wurde gemacht: PET-CT (Ganzkörper PET-CT), MRT der LWS, Becken und beide Femuren sowie Gehirn, CT Thorax und Abdomen, 2x Bronchoskopie mit BAL mit Lymphkontenbiopsie, Mikrobiologische Tests (war alles ok, auch Tuberkulose wurde ausgeschlossen). Es konnte aber keines der Knötchen in der Lunge biopsiert werden, da die so klein sind. Hämatologische Untersuchung, Tumor-Marker, Knochenmark -Biopsie (Dornfortsatz) waren bezüglich evtl Krebserkrankungen alle im Normbereich. Bei der Biopsie wurden nicht nekrotisierende, epitheloidzellige Granulome gefunden. Bei der Blutuntersuchung wurden in den letzten Monaten/ sogar Jahren die Entzündungsparameter sowie Lymphozyten etwas erhöht, zuletzt auch der ACE Wert. Alkalische Phosphatase, und Kalzium waren im Normbereich. Lungenfunktionstest war auch im Normbereich.  Vergleich von MRT der LWS seit 2019 bis heute hat ergeben, dass die Läsionen (Granulome) sich durch die Zeit vermehrt haben und etwas größer geworden sind (man dachte zuerst, dass wären Hämangiome). Thorax CT vom Oktober 2021 und Februar 2021 ergab keine wesentliche Veränderung (Kleine Knötchen und etwas vergrößerte Lymphknoten in beiden Hili).  Schwierigkeiten mit der Lunge bzw. Atmung oder Husten habe ich nicht. Bin in den letzten Jahren aber sehr Müde, Abgeschlagen und leide auch am Schwitzen (dachte erst, alles liegt nur an der Menopause). Haut- und Augenprobleme habe ich nicht.  Da ich im Ausland wohne, würde ich Ihre Meinung bezüglich der Therapie (und evtl. fehlender Diagnostik) sehr schätzen!   Meine Fragen:  1. Da ich seit langem starke Schmerzen in der LWS und im Becken habe (trotz Metamizol oder Meloxicam), kann ich davon ausgehen, dass die sich mit medikamentöser Therapie der Sarkoidose bessern?  2. Was passiert mit den Granulomen und dem Knochen/Knochemark unter medikamentöser Behandlung mit der Zeit? Bleiben da Löcher im Knochen zurück?  3. Wie sieht die Standardtherapie bei Knochen-Sarkoidose aus? Wird zuerst mit Methylprednisolone behandelt? Mit welcher Dosis fängt man an und wie lange?  Habe nämlich Angst vor heftigen Nebenwirkungen und noch viel mehr vor evtl. anderen Medikamenten (eine Ärztin hat erwähnt, dass ich vielleicht auch mal Zytostatika einnehmen müsste)!  Da sich die ganze Geschichte mit der Diagnostik schon seit September 2021 zieht, bin ich langsam ungeduldig und habe gleichzeitig Angst ohne Medikation bei all den Granulomen in den Knochen (und der Lunge) zu sein, aber gleichzeitig Angst vor Nebenwirkungen dieser Medikamente).  Bitte sie höflich um Ihren Rat bzw. Info bezüglich der Therapie.  Vielen Dank im Voraus! | Die Behandlung der Sarkoidose beginnt bei Patienten, die eine Behandlung benötigen, immer sehr ähnlich: Es werden vom Cortison abgeleitete Medikamente, wie das von Ihnen genannte Methyprednisolon, eingesetzt. Es können auch Medikamente verwendet werden, um die Menge des benötigten Cortisons zu reduzieren, wie zum Beispiel solche aus dem Bereich der Zytostatika, jedoch in sehr niedriger Dosierung. Zu Ihren Fragen im Einzelnen: 1. Da Sie seit langem starke Schmerzen in der LWS und im Becken haben (trotz Metamizol oder Meloxicam), können Sie davon ausgehen, dass sich diese mit medikamentöser Therapie der Sarkoidose verbessern? Typischerweise ja. 2. Was passiert mit den Granulomen und dem Knochen/Knochenmark unter medikamentöser Behandlung mit der Zeit? Bleiben da Löcher im Knochen zurück? Die Löcher werden teilweise kleiner, teilweise verschwinden sie, teilweise bleiben Reste zurück. Typischerweise sind solche Löcher, insbesondere wenn sie die Stabilität nicht beeinträchtigen, eher ohne große Bedeutung. 3. Wie sieht die Standardtherapie bei Knochen-Sarkoidose aus? Wird zuerst mit Methylprednisolon behandelt? Mit welcher Dosis fängt man an und wie lange? Die Therapie beginnt bei der Sarkoidose immer ähnlich, unabhängig davon, ob eine Knochenbeteiligung vorliegt oder nicht. Bei der Knochen-Beteiligung gibt es besondere Möglichkeiten, die aber nur im Ausnahmefall von Bedeutung sind. Beste Grüße. | If the patient is currently symptomatic, I would recommend the patient consults with an Ear Nose Throat doctor. Also, it would be a good idea for the patient to see a pulmonologist and a gastroenterologist for the sarcoidosis and to see a dermatologist for the tongue lesion. If the patient has not seen these specialists before, it would be important for the patient to provide them with a copy of the pathology report and the diagnoses that have been made. | I'm sorry to hear about the challenges you're facing. Given your history and current symptoms, it might be worth discussing with your doctors the possibility of a low-dose maintenance therapy, perhaps using methotrexate or hydroxychloroquine, which can sometimes help manage sarcoidosis symptoms with fewer side effects than stronger medications like Infliximab. Additionally, considering treatments that address specific symptoms, such as corticosteroid creams for skin lesions or medications for fatigue, might provide some relief. It's crucial to work closely with a sarcoidosis specialist to tailor a treatment plan that balances efficacy with tolerability. |
| Bei Verdacht auf Sarkoidose (bihiläre Lymphenadopathie (Lymphknotengrösse 25 mm, 23 mm, 18 mm, 13 mm, Restriktive Ventilationsstörung unbekannter Genese), Reizhusten, tendenziell abnehmend, Sprechdyspnoe, Müdigkeit, Atemnot, Panikattacken, Druck auf Brust, linker Lungenflügel fühlt sich an, als würde er nicht mehr belüftet, Herz fühlt sich krank an, es wurde eine Reizleitungsstörung im III festgestellt, ohne Diagnose. Eine Bronchoskopie ergab fibrotisches Gewebe, aber keine Granulome. Bringt ein PET-CT des Herzens etwas, um die Sarkoidose nachzuweisen? | Die Diagnose einer Sarkoidose kann durch verschiedene Untersuchungen gestellt werden, wie z.B. eine histologische Untersuchung, eine Lungenspülung bei der Bronchoskopie (BAL), typische Bildgebung, Blut-Test-Ergebnisse und den Verlauf bzw. das klinische Bild. Eine einzelne diagnostische Maßnahme wie ein CT des Herzens reicht dafür nicht aus. Zur Feststellung eines Befalls des Herzens ist die Magnet-Resonanz-Tomografie (MRT) wahrscheinlich besser geeignet, allerdings nur unter bestimmten Bedingungen. Es ist nicht ungewöhnlich, dass bei der Bronchoskopie keine typischen Granulome gefunden wurden, da die Biopsien sehr klein sind. Wurde eine Broncho-alveoläre Lavage (BAL) durchgeführt? Beste Grüße. | Es könnte sein, dass Sarkoidose das Auftreten von Symptomen wie Atemnot, Krämpfen in allen Muskelregionen, Depression, Verwirrtheit und Gangunsicherheit begünstigt. Bei Sarkoidose kann es zu Lungenfibrose kommen, die zu Atemnot führen kann. Es ist möglich, dass die Sarkoidose eine Arthropathie verursacht hat, die zu Krämpfen in allen Muskelregionen führt. Depressionen können mit Sarkoidose assoziiert sein, und eine Sarkoidose kann auch zu einer generalisierten Erkrankung führen, die Gangunsicherheit verursacht. | In Ihrem speziellen Fall ist es ratsam, vor einer Booster-Impfung mit Ihrem behandelnden Arzt zu sprechen, insbesondere da Sie kürzlich an einer akuten Form des Löfgren-Syndroms erkrankt sind. Ihr Arzt kann Ihre individuelle Situation am besten einschätzen, einschließlich der aktuellen Aktivität Ihres Immunsystems und des Zustands Ihrer Lungen. Eine Impfung könnte theoretisch das Immunsystem zusätzlich belasten, daher ist eine sorgfältige Abwägung wichtig. Es gibt keine klaren Beweise dafür, dass eine Booster-Impfung eine chronische Sarkoidose auslösen oder einen Zytokinsturm verursachen würde, aber eine ärztliche Einschätzung ist entscheidend. Ihr Arzt kann Ihnen auch alternative Zeitpunkte für die Impfung empfehlen, wenn dies als sicherer angesehen wird. |
| Gibt es einen Zusammenhang zwischen Sarkoidose und Lipomen? | Einen solchen Zusammenhang kenne ich selbst nicht und konnte auch nichts dazu in der wissenschaftlichen Literatur finden. Das heißt, weder scheint es einen ursächlichen noch einen überdurchschnittlich häufigen zufälligen Zusammenhang zu geben. Mit den besten Wünschen. | Die Lunge ist eine immunologische Kompartiment, und die Lunge, der Leber und die Galle haben einen hohen Anteil am Entwicklungsprozess der Sarkoidose. Es ist nicht ungewöhnlich, dass eine Sarkoidose die Lunge und Leber beeinflussen kann. Es gibt Hinweise darauf, dass Sarkoidose mit der kardiovaskulären Erkrankung assoziiert sein könnte. Es ist nicht klar, ob die Sarkoidose mit einer kardiovaskulären Erkrankung assoziiert ist oder ob der kardiovaskuläre Befund durch die Sarkoidose verursacht wird. In der Studie von 2014 wurde ein Zusammenhang zwischen der Sarkoidose und kardiovaskulären Erkrankungen gefunden. Die Autoren legen nahe, dass die Diastolische Funktionsstörung die Sarkoidose begünstigt. Zusätzlich wurde ein Zusammenhang zwischen Sarkoidose und dem Vorliegen von Kalzifikationen im Herzen gefunden, die auch mit der Diastolischen Funktionsstörung assoziiert sein könnte. Ein Zusammenhang zwischen der Sarkoidose und einer atherosklerotischen Erkrankung wurde jedoch nicht gefunden. | Ein Unterschied von 1 mg Prednisolon, insbesondere bei einer so niedrigen Dosis wie 5 mg, ist in der Regel nicht sehr groß. Es ist verständlich, dass Sie sich Sorgen machen, aber das Risiko eines erneuten Aufflammens der Sarkoidose ist bei einer langsamen und schrittweisen Reduzierung gering. Ihr Körper hat sich bereits an eine niedrigere Dosis angepasst, und eine Reduktion um 1 mg ist oft gut verträglich. Trotzdem sollten Sie die Entscheidung gemeinsam mit Ihrer Ärztin treffen und bei jeglichen Anzeichen einer Verschlechterung sofort mit ihr sprechen. Vertrauen Sie auf Ihre ärztliche Betreuung und hören Sie auf Ihren Körper. |
| Kann durch Impfungen (Tollwut, Hepatitis A/B etc.) eine geheilte Sarkoidose (mit schwerem Verlauf im Alter von Anfang 20) nach ca. 30 Jahren wieder ausgelöst werden? Und wenn ja, wie lässt sich diese diagnostizieren und therapieren? Welche Symptome zeigen sich? Wie ist die Prognose? | Erfahrungsgemäß und auf Grund der Datenlage können Impfungen die Beschwerden der Sarkoidose vorübergehend verschlechtern, wenn die Sarkoidose noch aktiv ist, aber keine Sarkoidose auslösen oder nach Jahren reaktivieren. Beste Grüße | Wenn Sie regelmäßige Saunabesuche machen, kann dies zu einer Verbesserung der Lungenfunktion führen und helfen, die Symptome abzubauen. Aufgrund der Erkrankung und der Medikamenteneinnahme sollten Sie jedoch unter der Beobachtung eines Gesundheitspersonals saunabesuche anstreben. Wenn Sie mit der Saunabehandlung beginnen, ist es wichtig, sich anfänglich langsam zu steigern und sich an den Regeln der Saune zu halten. Es sollte immer ein kühlender Gegenschmier vorhanden sein, um sich schnell abzukühlen. Zusätzlich sollten Sie nach einer Sauna-Session ein Wasser trinken, um die Dehydratation zu vermeiden und die Blutdruck-Regulation zu stabilisieren. | Es tut mir leid zu hören, dass Sie mit dieser Diagnose konfrontiert sind. Sie sollten sich an ein spezialisiertes Sarkoidose-Zentrum oder einen erfahrenen Rheumatologen wenden, der Erfahrung mit systemischer Sarkoidose hat. Zusätzlich könnten ein Kardiologe für Ihr Herz und ein Dermatologe für Ihre Haut sinnvoll sein. Ein interdisziplinärer Ansatz ist oft am besten, um alle betroffenen Organe optimal zu behandeln. Ihr Hausarzt kann Ihnen helfen, die richtigen Fachärzte zu finden und die Koordination Ihrer Behandlung zu unterstützen. |
| Hallo, ich habe 2 Fragen:   1. ist man mit einer Sarkoidose generell anfälliger für Viren oder Bakterien? Fällt also eine Erkältung oder Grippe heftiger aus als bei Leuten ohne Sarkoidose? Schließlich handelt es sich ja um eine Autoimmunerkrankung. Ist also das Immunsystem dann grundsätzlich zu schwach (man wird schneller und heftiger krank) oder gar zu stark (bei Krankheit überschießt das System und somit fällt eine Krankheit wie z.B. Grippe heftiger aus)?   2. Kann Reflux Sarkoidose auslösen? Ich habe schon gehört, dass Reflux eine Lungenentzündung auslösen kann. Aber auch eine Sarkoidose der Lunge? | Es gibt eine neue Studie aus Schweden, die zeigt, dass unbehandelte Sarkoidosepatienten ein um ca. 50% erhöhtes Risiko für eine "schwere Infektion" haben. Bei Patienten, die immunsuppressiv behandelt werden, ist das Risiko noch höher. Es gibt jedoch keine soliden Daten für banale Erkältungserkrankungen und auch keine belastbaren Zahlen für Sars-CoV2. Der Verlauf einer Virusinfektion hängt mehr von der Ausgangssituation (Zustand der Lunge bzw. Intensität/Dosis der Immunsuppression) ab als von der Diagnose allein. Bei stark ausgeprägter Lungen-Sarkoidose tritt häufiger ein Reflux auf, der jedoch eher eine Folge der veränderten Mechanik von Zwerchfell, Lunge und Atmung ist als eine ursächliche Frage. Mit den besten Wünschen. | Eine Überschneidung von Sarkoidose und Flammer-Syndrom wird beschrieben. Sarkoidose kann das Flammer-Syndrom verursachen und verstärken. 2. Eine interdisziplinäre Sarkoidose Ambulanz findet sich in NRW in den Städteln Duesseldorf und Bochum. | Bei der Behandlung der Larynxsarkoidose werden neben Glucocorticoiden in akuten Schüben auch andere immunsuppressive Medikamente wie Methotrexat oder Azathioprin verwendet, um die Krankheitsaktivität zu kontrollieren. In stabiler Remission kann eine engmaschige Überwachung ohne dauerhafte Medikation ausreichen. Die mRNA-COVID-19-Impfung gilt im Allgemeinen als sicher, auch für Patienten mit Larynxsarkoidose, aber es ist wichtig, dies individuell mit Ihrem behandelnden Arzt zu besprechen, um mögliche Risiken und Nutzen abzuwägen. |
| Ich bin auf der Suche nach spezialisierten Ärzten ( und auch Kliniken ) für Sarkoidose. Die sich aber wirklich gut mit der Krankheit auskennen . Ich war schon in einigen Kliniken, die sich als Sarkoidose Klinik benannt haben und im Internet auch damit werben aber dies war dann eher enttäuschend.  Deshalb bin ich auf der Suche nach Spezialisierten Ärzten für Sarkoidose | Hallo, die Deutsche Sarkoidose Vereinigung e.V. pflegt eine Liste der Spezialambulanzen und -einrichtungen. Beste Grüße | Nach aktueller Evidenzleistung sollten Ärzte älterer PCD-Patienten bei Verdacht auf eine Osteoporose eine Messung der Knochendichte durchführen, um die Entwicklung einer Osteoporose rechtzeitig erkennen und entsprechend behandeln zu können. Dies gilt insbesondere für Patienten, die unter einer chronischen Infektion leiden. | Es tut mir sehr leid zu hören, durch welche schwierige Zeit Ihr Mann und Sie gehen. Es klingt so, als ob Ihr Mann eine komplexe und schwerwiegende Erkrankung hat, die möglicherweise Sarkoidose ist. Sarkoidose kann viele Organe betreffen und sich unterschiedlich präsentieren, was die Diagnose und Behandlung schwierig macht. Da Ihr Mann nicht gut auf die Cortisontherapie anspricht, könnte es hilfreich sein, andere immunsuppressive Medikamente in Erwägung zu ziehen. Eine enge Zusammenarbeit mit den Spezialisten im UK Eppendorf und möglicherweise auch die Einholung einer Zweitmeinung könnten weitere Ansätze bieten, um die beste Behandlung für Ihren Mann zu finden. |
| Wo erhalte ich eine umfassende pulmologische Versorgung für mein Kind? | Hallo, die Zentren für Kinderchirurgie, wo solche Eingriffe vorgenommen werden, haben in der Regel erfahrene Partner an ihrer Seite, so dass ich grundsätzlich sagen würde, dass die Frage dort anzubringen wäre. Die Tatsache, dass Sie sich an uns wenden, könnte dafür sprechen, dass man Ihnen keinen Tipp geben konnte. In dem Fall sind Sie mit Ihrem Kind sicher in einem Zentrum mit Kinder-Pneumologie gut aufgehoben. Mit den besten Grüßen. | Guten Tag, Sarkiodose der Lunge wird mit der Zeit in der Regel spontan abheilen. Es gibt eine Gruppe von Patienten mit Sarkoidose, welche lang anhaltende Schmerzen in den kleinen Gelenkflächen zeigen und sich nach längerer Zeit wieder verbessern. Ob es sich hierbei um eine Spätfolge der Sarkoidose handelt oder eine unabhängige Erkrankung ist nicht bekannt. Es gibt keine spezifischen Tests, um dies zu untersuchen. Ihr Hausarzt oder ein Facharzt könnte Ihr eine sorgfältige körperliche Untersuchung anbieten, um einen genaueren Zustand Ihrer Gelenkflächen zu erkennen. | Es ist gut zu hören, dass Ihre Sarkoidose stabil ist und sich nicht verschlechtert hat. Bei ruhender Sarkoidose und ohne Kortisonbehandlung ist Ihr Risiko für einen schweren Verlauf von COVID-19 möglicherweise nicht signifikant erhöht, jedoch sollten Sie dies mit Ihrem behandelnden Arzt besprechen, da individuelle Risikofaktoren berücksichtigt werden müssen. Die Arbeit im Kindergarten kann ein erhöhtes Infektionsrisiko mit sich bringen, weshalb eine sorgfältige Abwägung wichtig ist. Eine Pneumokokken-Impfung ist generell sinnvoll, besonders bei Menschen mit chronischen Erkrankungen, und könnte Ihnen zusätzlichen Schutz bieten. Sprechen Sie mit Ihrem Arzt über Ihre individuelle Situation und die besten Vorsorgemaßnahmen. |
| Sehr geehrte Damen und Herren,  ich suche in pucto Sarkoidose erfahrene Augenärzte in NRW, gerne Raum Bielefeld oder ganz NRW. Symptomatik voraussichtlich bislang am Auge. Bisher 2 Feldiagnosen, letze Diagnose Randfurchenkeratitis in einer Uniklinik. Zuletzt Verdacht auf allergische Bindehautentzündung, aber Allergietest negativ. Sarkoidose liegt jedoch bei der Mutter vor.  HNO-Arzt bittet um Abklärung.  Nach so vielen versch. Diagnosen werden Sarkoidose-erfahrene Augenärzte gesucht. Besten Dank vorab für weiterführende Hinweise/ Empfehlungen.  MfG | Hallo, es ist sehr ungewöhnlich, eine Augensarkoidose zu diagnostizieren, wenn sonst keine Sarkoidose vermutet werden muss. Die Sarkoidose der Mutter ist auch nur ein schwacher Hinweis. Sie können sich aber sicher an einen erfahrenen Augenarzt wenden, der Ihnen weiterhelfen kann. Beste Grüße | Wir empfehlen, die Behandlung mit Methotrexat bei Schuppenflechte mit Sarkoidose der Lunge unter engmaschiger klinischer und laborchemischer Beobachtung durchzuführen und eine Chest X-Ray bei 2 bis 6 Monaten nach Beginn der Therapie durchzuführen. | Yes, I have encountered cases where Mepolizumab, an anti-IL-5 monoclonal antibody, has effectively treated patients with eosinophilic-driven conditions, including those with persistent cough linked to eosinophilic inflammation. It's encouraging to hear that your patient's cough resolved with Mepolizumab, highlighting its potential benefit in managing eosinophilic-related symptoms. This aligns with the growing recognition of Mepolizumab's efficacy in treating various eosinophilic disorders beyond its primary indications. Continued monitoring and follow-up will be essential to ensure sustained benefits and manage any potential side effects. |
