# Supplementary material for: MedBot vs RealDoc: efficacy of large language modeling in physician-patient communication for rare diseases
Source: J Am Med Inform Assoc. 2025 Feb 25;32(5):775–83. doi: 10.1093/jamia/ocaf034 (PMC12012358; doi:10.1093/jamia/ocaf034)
Supplement: ocaf034_Supplementary_Data [file ocaf034_supplementary_data.zip › ocaf034_Supplementary_Data/Supplementary_Material_B.docx]

The *Q*-Values, *Z*-Values and *p*-Values for the Dunn’s tests computed for the four different categories, correctness, comprehensibility, relevance and empathy.

|  |  | **GPT-4 vs Physicians** | **GPT-4 vs BioMistral 7B** | **Physicians vs BioMistral 7B** |
| --- | --- | --- | --- | --- |
| CORRECTNESS | Q-Value | -190.272 | -326.056 | 135.784 |
|  | Z-Value | -8.026 | -13.754 | 5.728 |
|  | p-Value | <0.001 | <0.001 | <0.001 |
| COMPREHENSIBILITY | Q-Value | -131.337 | -262.136 | 130.799 |
|  | Z-Value | -5.616 | -11.209 | 5.593 |
|  | p-Value | <0.001 | <0.001 | <0.001 |
| RELEVANCE | Q-Value | -181.025 | -281.604 | 100.579 |
|  | Z-Value | -7.670 | -11.932 | 4.262 |
|  | p-Value | <0.001 | <0.001 | <0.001 |
| EMPATHY | Q-Value | -209.058 | -264.661 | 55.603 |
|  | Z-Value | -8.790 | -11.128 | 2.338 |
|  | p-Value | <0.001 | <0.001 | 0.058 |

The *H*-values and *p*-Values for the Kruskal-Wallis tests computed for the four different categories, correctness, comprehensibility, relevance and empathy.

| CORRECTNESS | *H*-value | 190.939 |
| --- | --- | --- |
|  | *p*-value | <0.001 |
| COMPREHENSIBILITY | *H*-value | 125.642 |
|  | *p*-value | <0.001 |
| RELEVANCE | *H*-value | 146.250 |
|  | *p*-value | <0.001 |
| EMPATHY | *H*-value | 137.702 |
|  | *p*-value | <0.001 |
