# Supplementary material for: MedBot vs RealDoc: efficacy of large language modeling in physician-patient communication for rare diseases
Source: J Am Med Inform Assoc. 2025 Feb 25;32(5):775–83. doi: 10.1093/jamia/ocaf034 (PMC12012358; doi:10.1093/jamia/ocaf034)
Supplement: ocaf034_Supplementary_Data [file ocaf034_supplementary_data.zip › ocaf034_Supplementary_Data/Supplementary_Material_C.docx]

*English Translation:*

**Question 1: To what extent could chatbot responses help to reduce the workload of doctors? Would you consider such a response as a first draft?**

1. To question 1: Reduction of working time through literature research, summarising the results of literature research, summarising examination results, recording and processing anamnesis interviews, final summary of anamnesis, ward round entries, examination results and therapy orders into a medical report. Formulation of recommendations based on current guidelines. Question 2: Yes.
2. I see this as a potential relief for doctors in the near future and not a bad thing at all
3. Chatbot answers could reduce the workload of doctors. Yes, such an answer could be considered as a first draft to provide basic information efficiently.
4. As supportive measures, I think such models make a lot of sense.

**Question 2: What potential risks do you see with the use of chatbots, especially in terms of misinformation or overlooking critical information?**

1. It would have to be ensured that literature and guideline citations actually exist and are relevant. Answers could be formally correct but not logical and accurate in terms of content.
2. May vary by patient and have consequences
3. Misinformation due to limited comprehension skills and the risk of overlooking critical information that requires personal judgement.
4. In any case, a medical review must be carried out. If this is done, I believe the risk of overlooking information and misinformation is low.

**Question 3: What considerations would you make before you consider using a chatbot in your practice?**

- - - 1. Are the statements really correct? How high is the risk of ‘invented’ texts? Who checks the answers? Which sources are used? How is data security guaranteed? How high are the costs? How reliable is the operation?
      2. Multiple test phase and evaluation
      3. Data security, accuracy of responses, integration into existing systems, ease of use for patients and legal/regulatory requirements.
      4. The purchase of such an AI solution depends on the frequency of enquiries and the costs of use.

**Question 4: How do you assess the ethical aspects of using a chatbot, particularly with regard to patient confidentiality?**

1. That depends on where confidentiality could be breached.
2. Can already be critical
3. The ethical aspects of chatbot use relate in particular to patient confidentiality, which must ensure that personal health data is protected and treated confidentially.
4. I assume that the necessary data protection standards will be complied with. I don't have any ethical concerns, as such models do bring benefits for patients and it won't be a stand-alone solution.

**Question 5: Are there any retrospective characteristics or indications in the answers that lead you to assume whether they come from a doctor or a chatbot? If so, which ones?**

1. Formally correct answers that nevertheless do not lead to a logical result. Answers that are difficult to understand in terms of content, sentence breaks.
2. *no answer given*
3. A doctor could probably explain more complex medical concepts in more detail and give more personalised treatment recommendations. A chatbot tends to give predefined answers that may be more general and less flexible.
4. It is unusual for studies to be cited directly in such a short answer. I don't think that's appropriate and possibly comes from an AI solution. There were also a few answers that completely missed the point of the question. The more colloquial answers presumably did not originate from AI.

**Question 6: Did you notice any major differences in the answers? If so, what differences did you notice?**

1. see previous answer
2. Sometimes very neat and very correct answers and sometimes very incorrect
3. Chatbot responses tended to be more general and less flexible, with predefined information that may not always cover all aspects of an individual situation.
4. The conscientiousness of the answers was very variable. In terms of content, most answers were good average.

**Question 7: What legal challenges do you see in the implementation of an AI-based chatbot in patient communication?**

1. The high hurdles of European and especially German data protection.
2. Major challenge in terms of data protection
3. Data protection and security, liability for medical errors, compliance with laws and regulations, clear responsibilities and patient consent.
4. As a pure support system, I don't see any problems here at all. ECG or lung function software have had initial AI findings for many years, but of course these were often incorrect. There should be no legal problems if a doctor's findings/cross-check is carried out.

*Original:*

**Frage 1: Inwiefern könnten Chatbot-Antworten dazu beitragen, die Arbeitslast von Ärzten zu reduzieren? Würden Sie eine solche Antwort als ersten Entwurf in Betracht ziehen?**

1. Zu Frage 1: Arbeitszeitreduktion durch Literaturrecherche, Zusammenfassung der Ergebnisse der Literaturrecherche, Zusammenfassung von Untersuchungsergebnissen, Erfassung und Verarbeitung von Anamnesegesprächen, abschließende Zusammenfassung von Anamnese, Visiteneinträgen, Untersuchungsergebnisssen und Therapieanordnungen zu einem Arztbericht. Formulierung von Empfehlungen auf Grundlage aktueller Leitlinien. Zu Frage 2: Ja.
2. Finde ich in nahen Zukunft als mögliche Entlastung für Ärzte und gar nicht so schlecht
3. Chatbot-Antworten könnten die Arbeitslast von Ärzten reduzieren. Ja, eine solche Antwort wäre als ersten Entwurf in Betracht zu ziehen, um grundlegende Informationen effizient bereitzustellen.
4. Als unterstützende Maßnahmen halte ich solche Modelle für sehr sinnvoll.

**Frage 2: Welche potentiellen Risiken sehen Sie bei der Verwendung von Chatbots, insbesondere in Bezug auf Fehlinformationen oder das Übersehen von kritischen Informationen?**

1. Es müsste sichergestellt sein, dass Literatur- und Leitlinienzitate tatsächlich existieren und relevant sind. Antworten könnten formal korrekt aber inhaltlich nicht logisch und zutreffend sein.
2. Kann je nach Patient variieren und mit sich Konsequenzen ziehen
3. Fehlinformationen aufgrund begrenzter Verständnisfähigkeiten und das Risiko, kritische Informationen zu übersehen, die eine persönliche Einschätzung erfordern.
4. Es muss auf jeden Fall ein ärztliches Gegenlesen erfolgen. Wird dies durchgeführt, halte ich das Risiko für das Übersehen von Informationen und Fehlinformationen für gering.

**Frage 3: Welche Überlegungen würden Sie anstellen, bevor Sie die Nutzung eines Chatbots in der Praxis in Erwägung ziehen?**

- - - 1. Sind die Aussagen wirklich korrekt? Wie hoch das Risiko „erfundener“ Texte? Wer überprüft die Antworten? Welche Quellen werden verwendet? Wie wird Datensicherheit gewährleistet? Wie hoch sind die Kosten? Wie verlässlich ist der Betrieb?
      2. Mehrfache Testphase und Auswertung
      3. Datensicherheit, Genauigkeit der Antworten, Integration in bestehende Systeme, Benutzerfreundlichkeit für Patienten und rechtliche/regulatorische Anforderungen.
      4. Die Anschaffung einer solchen KI-Lösung hängt von der Frequenz der Anfragen und den Nutzungskosten ab.

**Frage 4: Wie bewerten Sie die ethischen Aspekte der Verwendung eines Chatbots, insbesondere hinsichtlich der Patientenvertraulichkeit?**

- - - 1. Das hängt davon ab, an welchen Stellen Vertraulichkeit relevant verletzt werden könnte.
      2. Kann schon kritisch sein
      3. Die ethischen Aspekte der Chatbot-Nutzung betreffen insbesondere die Patientenvertraulichkeit, die sicherstellen muss, dass persönliche Gesundheitsdaten geschützt und vertraulich behandelt werden.
      4. Ich gehe davon aus, dass die notwendigen Datenschutzstandards eingehlaten werden. Ethische Bedenken habe ich nicht, da solche Modelle ja durchaus Patientenvorteile bringen und es keine "stand alone"-Lösung wird.

**Frage 5: Gibt es retrospektiv Merkmale oder Hinweise in den Antworten, die Sie vermuten lassen, ob sie von einem Arzt oder einem Chatbot stammen? Wenn ja, welche?**

- - - 1. formal korrekte Antworten, die trotzdem zu keinem logischen Ergebnis führen. Inhaltlich schwer verständliche Antworten, Satzabbrüche.
      2. *keine Antwort*
      3. Ein Arzt könnte wahrscheinlich komplexere medizinische Konzepte detaillierter erklären und individuellere Behandlungsempfehlungen geben. Ein Chatbot neigt dazu, vordefinierte Antworten zu geben, die möglicherweise allgemeiner und weniger flexibel sind.
      4. Es ist ungewöhnlich dass direkt in so einer kurzen Antwort Studien zitiert werden. Das halte ich nicht für geeignet und entstammt möglicherweise einer KI-Lösung. Auch gab es wenige Antworten, die an der Frage komplett vorbei gingen. Die etwas umgangssprachlicheren Antworten entstammen mutmaßlich nicht der KI.

**Frage 6: Haben Sie gravierende Unterschiede in den Antworten bemerkt? Wenn ja, welche Unterschiede sind Ihnen aufgefallen?**

- - - 1. siehe vorherige Antwort
      2. Manchmal sehr ordentlich und sehr korrekte Antworten und manchmal sehr fehlerhaft
      3. Chatbot-Antworten neigen dazu, allgemeiner und weniger flexibel zu sein, mit vordefinierten Informationen, die möglicherweise nicht immer alle Aspekte einer individuellen Situation abdecken.
      4. Die Gewissenhaftigkeit der Antworten war sehr variabel. Inhaltlich waren die meisten Antworten gutes Mittelmaß.

**Frage 7: Welche rechtlichen Herausforderungen sehen Sie bei der Implementierung eines KI-basierten Chatbots in der Patientenkommunikation?**

- - - 1. Die hohen Hürden des europäischen und insbesondere deutschen Datenschutzes.
      2. Große Herausforderung was die Datenschutz angeht
      3. Datenschutz und -sicherheit, Haftung bei medizinischen Fehlern, Einhaltung von Gesetzen und Vorschriften, klare Verantwortlichkeiten und Patienteneinwilligung.
      4. Als reines Unterstützungssystem sehe ich hier überhaupt keine Probleme. EKG- oder Lungenfunktionssoftware haben ja seit vielen Jahren erste KI-Befunde, die aber natürlich oft auch falsch waren. Wenn ein ärztlicher Befund/ein ärtzliches Gegenlesen erfolgt, sollte es keine rechtlichen Probleme geben.
